# Preparation of cyclic imides from alkene-tethered amides: application of homogeneous Cu(ii) catalytic systems†

**DOI:** 10.1039/c9ra10422d

**Published:** 2020-02-21

**Authors:** Zhenghui Liu, Peng Wang, Hualin Ou, Zhenzhong Yan, Suqing Chen, Xingxing Tan, Dongkun Yu, Xinhui Zhao, Tiancheng Mu

**Affiliations:** School of Pharmaceutical and Materials Engineering, Taizhou University Taizhou 318000 Zhejiang China liuzhenghui@iccas.ac.cn; Beijing National Laboratory for Molecular Sciences, CAS Research/Education Center for Excellence in Molecular Sciences, Institute of Chemistry, Chinese Academy of Sciences Beijing 100190 China; Key Laboratory of Green Chemical Media and Reactions, Ministry of Education, School of Chemistry and Chemical Engineering, Henan Normal University Xinxiang 453007 Henan China; Department of Chemistry, Renmin University of China Beijing 100872 China tcmu@ruc.edu.cn

## Abstract

A Cu-based homogeneous catalytic system was proposed for the preparation of imides from alkene-tethered amides. Here, O_2_ acted as a terminal oxidant and a cheap and easily available oxygen source. The cleavage of C=C bonds and the formation of C–N bonds were catalyzed by Cu(ii) salts with proper nitrogen-containing ligands under 100 °C. The synthesis approach has potential applications in pharmaceutical syntheses. Moreover, scaled-up experiments confirmed the practical applicability.

## Abstract 2

A catalytic system comprising Cu(ii) and a nitrogen-based ligand for the oxygenation and cyclization of alkene-tethered amides.

## Graphical abstract

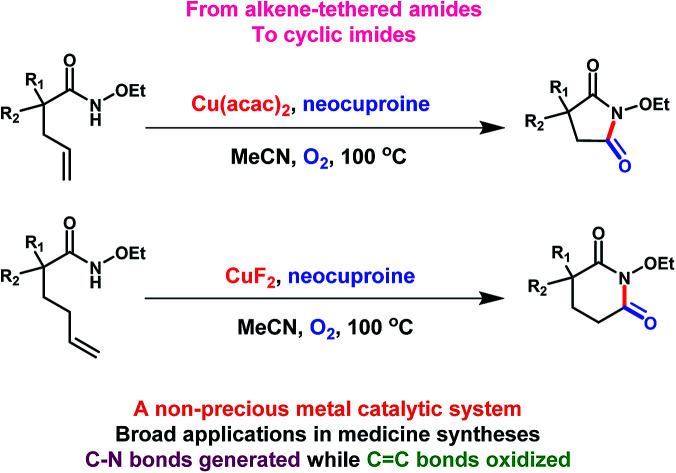

## 1. Introduction

**Scheme 1:**
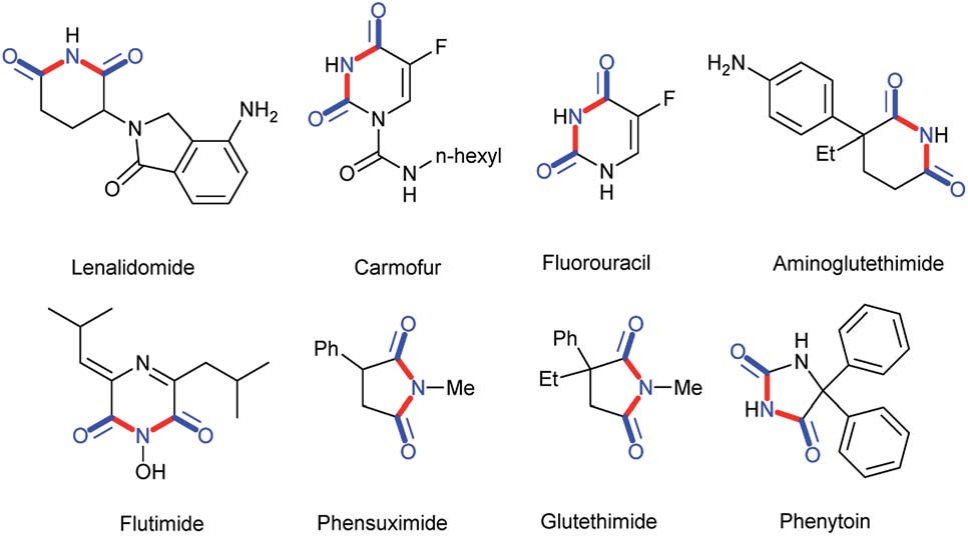
Pharmaceutical molecules with cyclic imide structures.

**Scheme 2:**
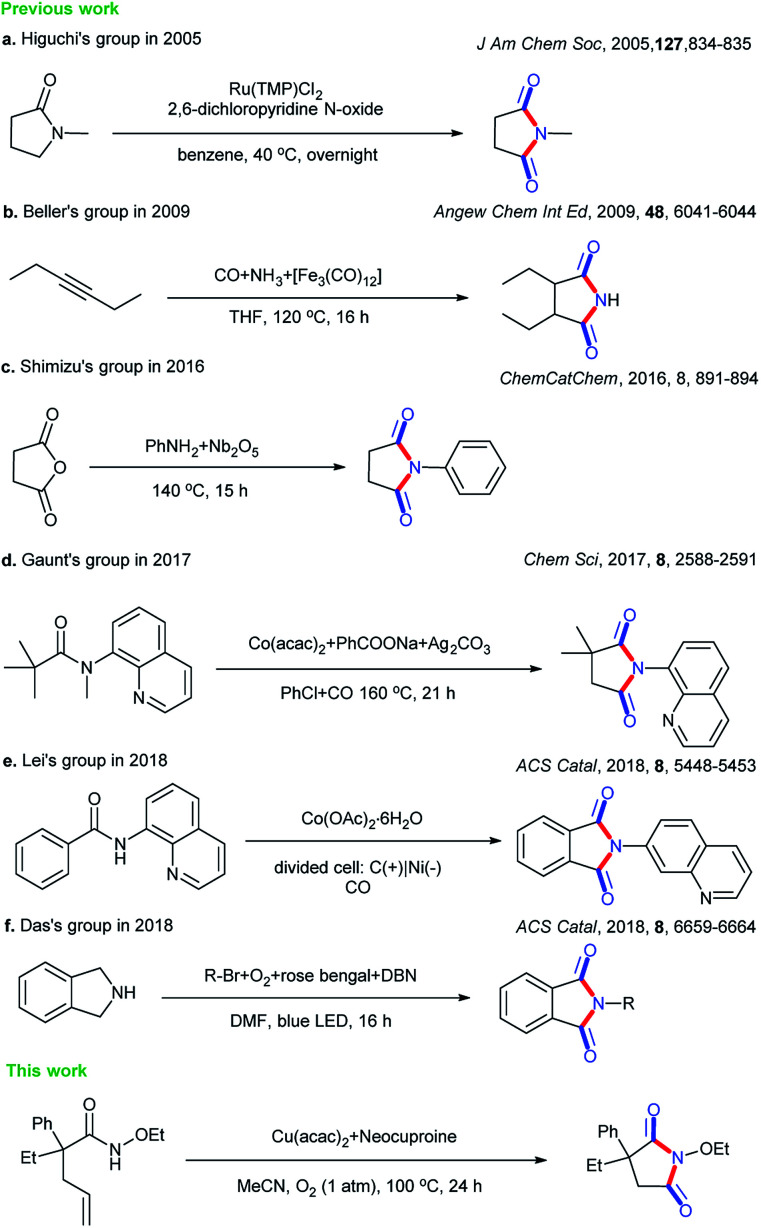
Different strategies for the syntheses of cyclic imides.

Cyclic imides are becoming increasingly popular in the pharmaceutical field.^1–5^ Some drug molecules bearing cyclic imide structures are shown in Scheme 1. Among them, lenalidomide, carmofur, fluorouracil, and aminoglutethimide are antineoplastic drugs; flutamide is an antiflu drug; and phensuximide, phenytoin, and glutethimide are antiepileptic, antiarrhythmic, and sedative-hypnotic drugs, respectively. The preparations of imides and cyclic imides have been paid much attention over the years.^6–9^ As shown in Scheme 2, certain methods were gradually proposed. In 2005, Higuchi *et al.* proposed an oxidation method of amides with 2,6-dichloropyridine *N*-oxide catalyzed by ruthenium porphyrin in benzene at a low temperature of 40 °C overnight (Scheme 2a).^10^ After that, more nonnoble metals were employed in this type of transformation. In 2009, Beller *et al.* translated hex-3-yne into cyclic imides with CO and NH_3_ catalyzed by [Fe_3_(CO)_12_] in THF at 120 °C for 16 h, which was the first report on the iron-catalyzed synthesis of succinimides *via* the carbonylation of diverse internal and terminal alkynes with amines or ammonia to achieve good selectivity and high activity (Scheme 2b).^11^ Later, metallic oxides were also found to be able to achieve such preparations. In 2016, Shimizu *et al.* completed the direct synthesis of cyclic imides by using carboxylic anhydrides and amines using Nb_2_O_5_ as a water-tolerant Lewis acid catalyst without solvents (Scheme 2c).^12^ In 2017, Gaunt *et al.* set up a Co-catalyzed carbonylative cyclization procedure of unactivated, aliphatic C–H bonds with a combination of Co(acac)_2_, PhCOONa, Ag_2_CO_3_, PhCl, and CO (Scheme 2d).^13^ In addition, other methods such as electrocatalysis and photocatalysis could also be used in this transformation (Scheme 2e and f).^14,15^

The valid and selective oxidation of organic compounds offers the opportunity for the streamlined conversion of simple precursors into value-added products.^16^ Oxidation reactions, together with polymerizations and carbonylations, constitute the largest industrial applications in the field of homogeneous catalysis, and substantial value-added bulk and fine chemicals can be fabricated through this technology.^17^ With the superiority of environmental compatibility, low cost, and high efficiency, O_2_ is becoming a frequently used oxidant in both experimental and industrial scenarios.^18,19^ With regard to green and sustainable chemistry, oxidants such as inorganic salts, IBX, oxone, BQ TBHP, DDQ, or PhI(OAc)_2_ suffer from many problems such as waste disposal, high cost, and poor atom economy.

Encouraged by the recently published articles and our interest in nonnoble-metal-catalyzed oxidization and cyclization reactions, herein, we developed an efficient, copper-based catalytic system comprising commercially available Cu salts and a bidentate nitrogen ligand for the preparation of cyclic imides from alkene-tethered amides in acetonitrile (MeCN) under atmospheric O_2_ at 100 °C.^20–22^

## 2. Results and discussion

### 2.1 Screening of metal salts and ligands

Metal salts coordinated with specific ligands exhibit excellent catalytic abilities.^23–29^ In the initial set of experiments, a series of frequently used metal salts coordinated with ligands containing N, P, or other elements were tested, and the results are shown in Table 1. Here, 10 types of transition metal salts (Fe, Co, Ni, Cu, Zn, Mn, Pd, Ru, Rh, and Ir salts) with 22 types of ligands (for detailed information, see ESI, Table S1†) were employed in the oxidation of alkene-tethered amide 1a into cyclic imide 2a in MeCN at 100 °C in an O_2_ environment under atmospheric pressure. Cu(acac)_2_ with ligand A (neocuproine) exhibited outstanding catalytic performance with the highest 2a yield of 85%, while the others behaved moderately or even badly under otherwise identical conditions. Phosphine ligands were not expected to be efficient in an O_2_ atmosphere due to inactivation caused by oxidation.^18^ Further, they indeed provided no yields (Table 1; for detailed yields, see ESI, Tables S2–S11†). In addition, all the tested Cu salts satisfactorily performed with yields over 75% (*e.g.*, Cu(BF_4_)_2_·2H_2_O: 82%; CuF_2_: 81%; CuCl_2_·2H_2_O: 80%), among which Cu(acac)_2_ gave the highest yield of 2a (Table S12†).

**Table 1:** Metal-catalyzed oxygenation of alkene-tethered amides to cyclic imides^a^

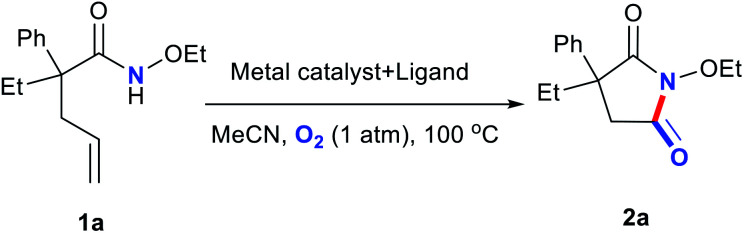			
Entry	Metal salt	Ligand	Yield^b^/%
1	FeCl_3_·6H_2_O	A–V	<1%^c^
2	Co(BF_4_)_2_·6H_2_O	A–V	<5%^c^
3	Ni(acac)_2_	A–V	<1%^c^
4	Cu(acac)_2_	A–V	Up to 85%^c^
5	ZnCl_2_	A–V	<1%^c^
6	MnCl_2_·4H_2_O	A–V	<1%^c^
7	PdCl_2_	A–V	<6%^c^
8	RuCl_3_	A–V	<5%^c^
9	RhCl_3_·3H_2_O	A–V	<6%^c^
10	IrCl_3_	A–V	<4%^c^
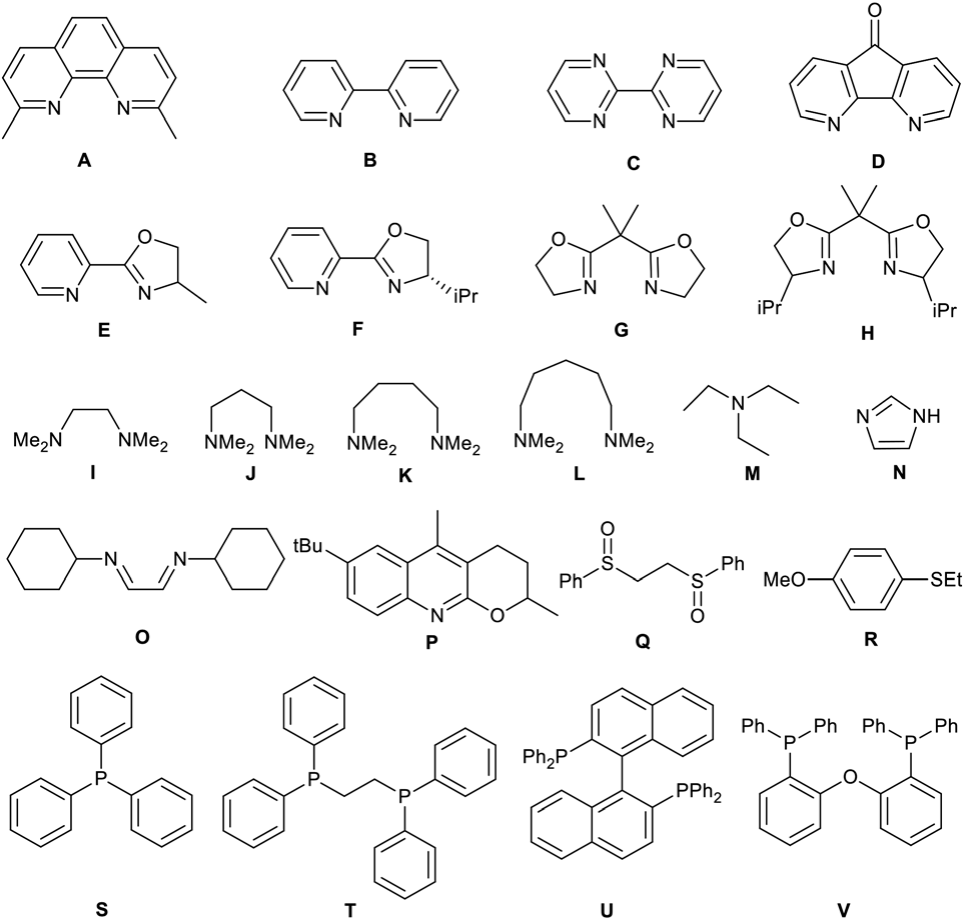			

a Reaction conditions: 1a, 0.1 mmol; metal salt, 0.01 mmol (10 mol%); ligand, 0.015 mmol (15 mol%); MeCN, 1.5 mL; O_2_ (1 atm); 100 °C; 24 h.

b Determined by ^1^H NMR analysis using 1,1,2,2-tetrachloroethane as the internal standard.

c For detailed yields, see ESI, Tables S2–S11.

### 2.2 Optimized dosage of Cu(acac)_2_ and neocuproine

**Fig. 1:**
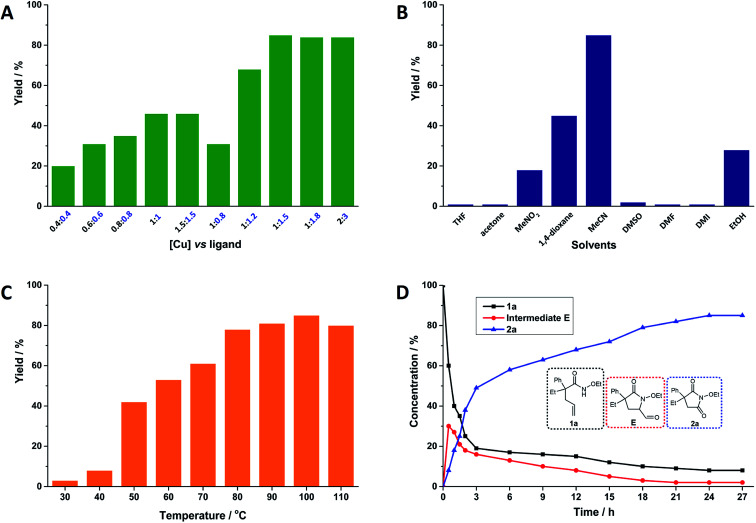
Oxygenation of alkene-tethered amides to cyclic imides using O_2_ catalyzed by Cu(acac)_2_ and neocuproine ligand system. Reaction conditions: 1a, 0.1 mmol; Cu(acac)_2_, 0.01 mmol (10 mol%); neocuproine, 0.015 mmol (15 mol%); MeCN, 1.5 mL; O_2_ (1 atm); 100 °C; 24 h; unless otherwise stated. Yields were determined by ^1^H NMR analysis using 1,1,2,2-tetrachloroethane as the internal standard (A) effects of dosage of Cu(acac)_2_ and neocuproine ligand (numbers denote the loading values of Cu(acac)_2_ and neocuproine in equiv. relative to substrate 1a); (B) effects of solvents; (C) effects of temperature; (D) kinetic analysis.

After confirming the suitable combination of Cu(acac)_2_ and neocuproine, the optimized ratio of Cu(acac)_2_ and neocuproine ligand was explored, as shown in Fig. 1A. Originally, the relative molar ratio of [Cu] *vs.* ligand was set at 1 *vs.* 1. As expected, the yields were higher when the loading values were higher (from 20% at 0.4 equiv. : 0.4 equiv. to 46% at 1 equiv. : 1 equiv.). However, after each loading was above 1 equiv., the yields stopped increasing (maintaining 46% at 1.5 equiv.). Then, the loading of neocuproine was adjusted further as the dosage of Cu(acac)_2_ was fixed at 1 equiv. As shown in Fig. 1A, when the loadings of neocuproine were 0.8, 1.0, 1.2, and 1.5 equiv., the yields were 31%, 46%, 68%, and 85%, respectively. This implied the strong dependency of neocuproine dosage in the system. However, a further increase in the neocuproine dosage to 1.8 equiv. did not help in increasing the yield. Finally, the loading was doubled as 2 equiv. : 3 equiv., and the yield was still unimproved. Therefore, the optimized dosage of Cu(acac)_2_ and neocuproine was 1 equiv. *vs.* 1.5 equiv.

### 2.3 Effects of solvents

Solvents acted as the reaction media and intensively influenced the catalytic reactions.^30–34^ The effects of nine representative solvents on the catalytic reaction were tested, and the results were summarized, as shown in Fig. 1B. Five nonpolar solvents were tested. Tetrahydrofuran (THF) and acetone showed nearly no activity in the system. Nitromethane (MeNO_2_) and 1,4-dioxane started to show some reactive abilities. Surprisingly, when the solvent was switched to MeCN, the highest yield was 85%. In addition, three high-boiling-point polar aprotic solvents, namely, dimethyl sulfoxide (DMSO), dimethyl formamide (DMF), and 1,3-dimethyl-2-imidazolidinone (DMI), were tested, but they yielded nearly no products. Finally, we tried ethanol (EtOH) as a protic solvent, and it showed a medium yield. Without doubt, MeCN was considered to be the most suitable solvent for this system.

### 2.4 Effects of temperature

To gain more specific information regarding the catalytic system, the reaction temperatures were set from 30 to 110 °C (shown in Fig. 1C). Here, 30 and 40 °C were too low to provide sufficient energy for the reaction. Temperatures ranging from 50 to 100 °C started to give satisfactory yields: a higher temperature was associated with a higher yield. However, when the temperature was further improved to 110 °C, the yield was slightly reduced perhaps due to the side reactions induced by the higher temperature. Therefore, 100 °C was considered to be the optimal temperature for this system.

### 2.5 Kinetic study

We conducted kinetic observations; the amounts of 1a and 2a were recorded and an aldehyde intermediate (1-ethoxy-4-ethyl-5-oxo-4-phenylpyrrolidine-2-carbaldehyde) was detected and recorded (shown in Fig. 1D). A clear initial increase in the aldehyde concentration was observed, and consumption subsequently occurred. In addition, the amount of 1a sharply decreased in the initial phase (for detailed information of the quantitative data, see ESI, Table S15†).

### 2.6 Effects of additives

In order to further increase the yield and efficiency of the reaction, 10 types of additives were tested (shown in Table 2). Unfortunately, none of them yielded better results. However, the incorporation of H_2_O did not reduce the yield, which implied that the catalytic system was robust against water and therefore could be applied to more industrial scenes.

**Table 2:** Cu(acac)_2_-catalyzed oxygenation of alkene-tethered amides to cyclic imidesa

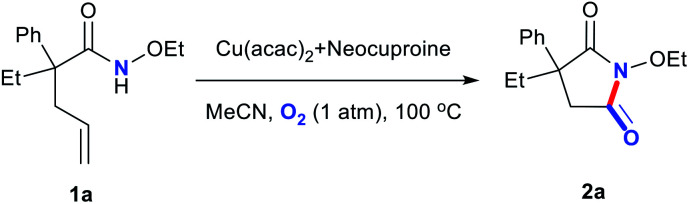			
Entry	Additive	Dosage^b^	Yield^c^/%
1	K_2_CO_3_	1 equiv.	18
2	KOAc	1 equiv.	22
3	DBU	1 equiv.	58
4	BMImAc	1.5 equiv.	62
5	BMImBr	1.5 equiv.	59
6	*n*-Bu_4_NF	1.2 equiv.	51
7	PhCOOH	1 equiv.	45
8	Methylsulfonic acid	1 equiv.	52
9	Acetic anhydride	1 equiv.	48
10	H_2_O	5 equiv.	83

a Reaction conditions: 1a, 0.1 mmol; Cu(acac)_2_, 0.01 mmol (10 mol%); ligand neocuproine, 0.015 mmol (15 mol%); MeCN, 1.5 mL; O_2_ (1 atm); 100 °C; 24 h.

b Dosage of additives was relative to 1a.

c Determined by ^1^H NMR analysis using 1,1,2,2-tetrachloroethane as the internal standard; DBU = 1,8-diazabicyclo[5.4.0]undec-7-ene; BMImBr = 1-butyl-3-methylimidazolium bromide; BMImAc = 1-butyl-3-methylimidazolium acetate.

### 2.7 Authentication of component necessity in the catalytic system

A series of control experiments were conducted with the lack of Cu salt, ligand, or O_2_ to confirm the indispensability of each component (Table S14†). Further, the results showed that without the Cu salt, ligand, or O_2_, the reaction could not proceed at all.

### 2.8 Scaled-up experiment

**Scheme 3:**
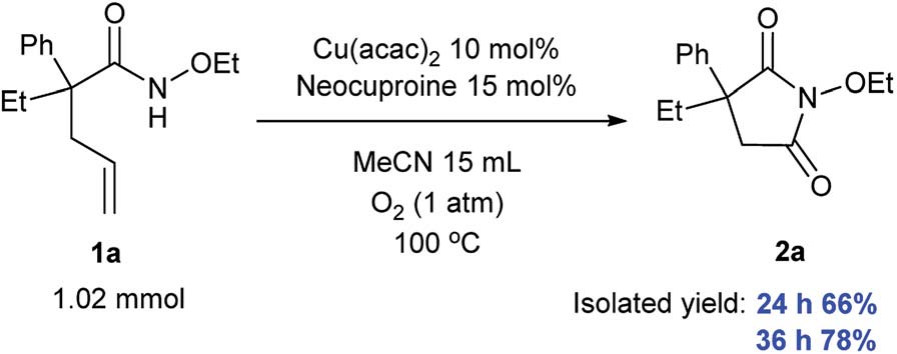
Scaled-up experiment.

Importantly, we conducted a large-scale experiment (larger by 10 times) and obtained a yield of 66% over 24 h and 78% over 36 h, indicating that our system can find valuable applications in industrial production, as shown in Scheme 3.

### 2.9 Substrate scope

**Scheme 4:**
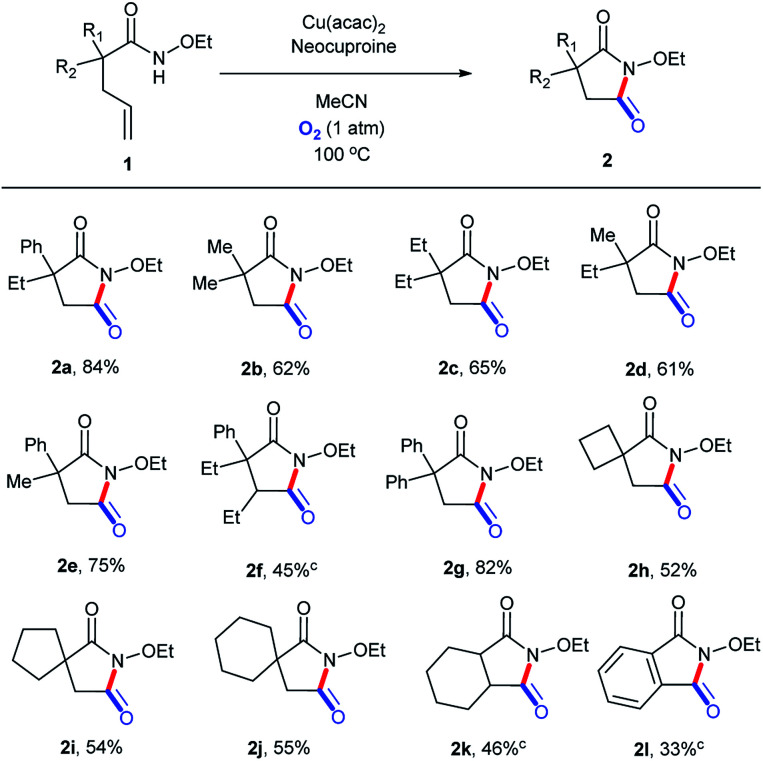
Substrate scope (for succinimide derivatives). ^*a*^Reaction conditions: substrate, 0.1 mmol; Cu(acac)_2_, 0.01 mmol (10 mol%); neocuproine, 0.015 mmol (15 mol%); MeCN, 1.5 mL; O_2_ (1 atm); 100 °C; 24 h. ^*b*^Isolated yields. ^*c*^48 h.

On the basis of the optimized reaction conditions, the scope of this transformation was explored, as shown in Scheme 4. To our delight, ethoxy could be well preserved during the entire process. Moderate to good yields were obtained, with the highest isolated yield of 84% (2a). A phenyl group in the molecular chain seemed to be in favor of the transformation (2b, 2c, and 2d*vs.*2e and 2g) perhaps due to the stabilization effect of the aromatic ring in the reaction course.^35^ Substitution beta to the amide led to a substantial decrease in the reaction yield even after longer reaction times (2f), presumably due to steric hindrance. Substrates with 4-, 5-, and 6-membered spirocycles in the alpha position could be tolerated, but with relatively lower yields (2h, 2i, 2j, and 2k). The 1l-containing conjugated alkene structure could be translated into 2l, but with a low yield (48 h) maybe because of the unanticipated oxidation process in the O_2_ atmosphere at 100 °C.

**Scheme 5:**
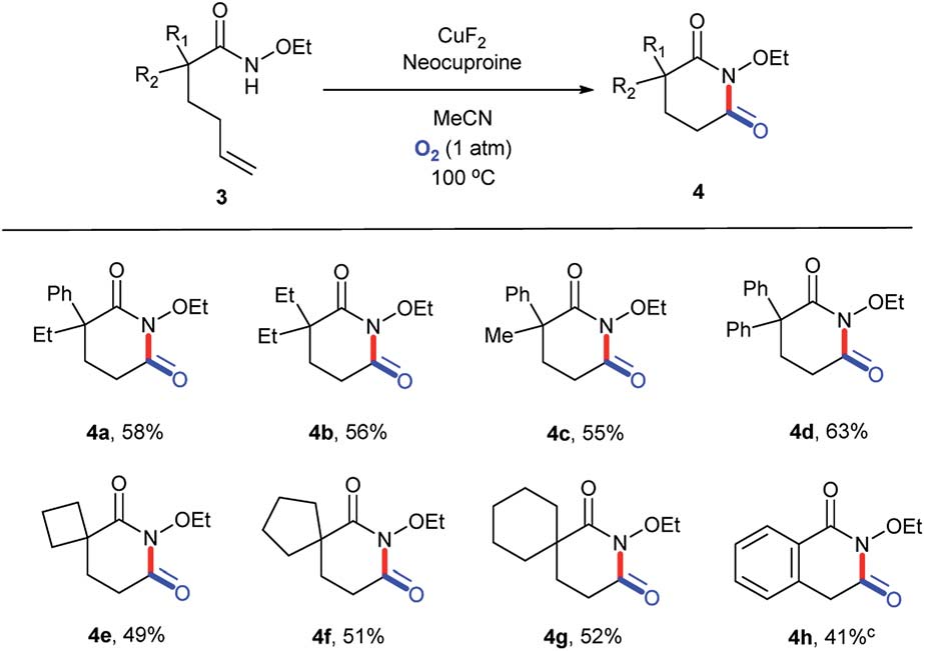
Substrate scope (for glutarimide derivatives). ^*a*^Reaction conditions: substrate, 0.1 mmol; CuF_2_, 0.01 mmol (10 mol%); neocuproine, 0.015 mmol (15 mol%); MeCN, 1.5 mL; O_2_ (1 atm); 100 °C; 24 h. ^*b*^Isolated yields. ^*c*^48 h.

To explore the versatility of this catalytic system, it was used to prepare glutarimide derivatives. Before that, we retested the copper salts and found that the best one was CuF_2_ (Table S13, ESI†). Further, a new round of substrate scope was demonstrated in Scheme 3. Products with a 6-membered ring exhibited lower yields (4a–4g, 49–63%) owing to the lower stability than the 5-membered ones.^36^ Similarly, 3h bearing a conjugated alkene structure still performed poorly (Scheme 5).

### 2.10 Reaction mechanism

**Scheme 6:**
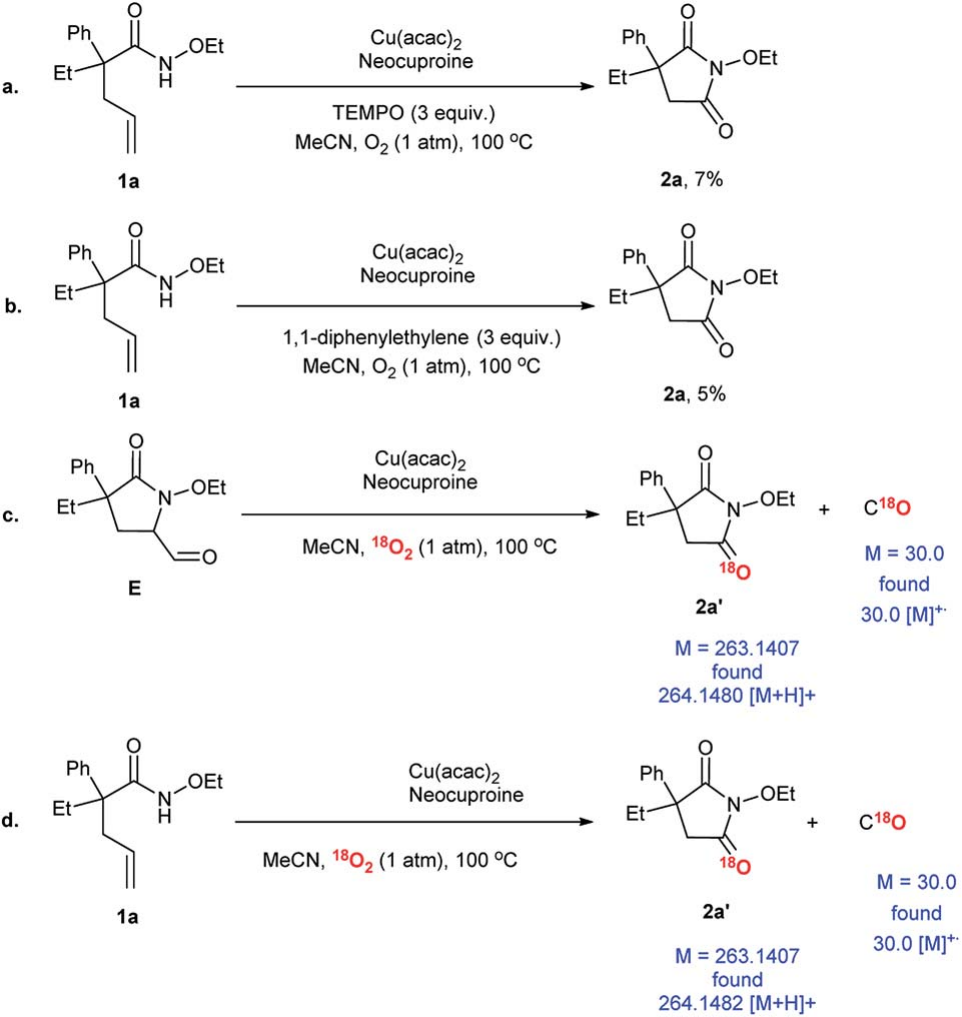
Mechanism research. ^*a*^Reaction conditions: substrate, 0.1 mmol; Cu(acac)_2_, 0.01 mmol (10 mol%); neocuproine, 0.015 mmol (10 mol%); MeCN, 1.5 mL; O_2_ (1 atm); 100 °C; 24 h.

In order to investigate the possible reaction mechanism, some control experiments and isotope-labeling experiments were conducted (Scheme 6). Two types of radical scavengers, namely, (2,2,6,6-tetramethylpiperidin-1-yl)oxyl (TEMPO) and 1,1-diphenylethylene, were employed to explore whether the transformation involved free radicals or not. The extremely low yields of 2a indicated that the free radicals participated in the process, which was doubtlessly consistent with earlier reports (Scheme 6a and b).^35,37–39^ Compound E was detected in the system and was considered to be an intermediate. Therefore, E was used as the substrate and O_2_ was replaced by ^18^O_2_ to track the O source. Further, product 2a′ with isotope-labeling was produced, as detected by HR-ESI (Scheme 6c). In addition, C^18^O was confirmed using GC-MS. Finally, a proof experiment was conducted (Scheme 6d). In conclusion, O_2_ took part in the reaction and became a carbonyl group in the final product; here, C^18^O was detected again.

**Scheme 7:**
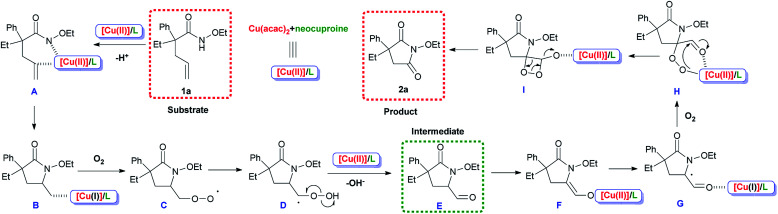
Proposed mechanism.

Considering the experimental results and earlier reports involving this type of metal/ligand system,^35,40–45^ a possible reaction pathway was proposed, as shown in Scheme 7. For precision and emphasis, the acac^−^ anion was omitted and therefore the electric charges were not shown. Copper salts coordinated with the N-ligand neocuproine to form the catalytically active species [Cu(ii)]/L. [Cu(ii)]/L replaced a proton in substrate 1a generating A, and the *cis*-amidocupration of the alkene occurred, affording organo-copper(ii) intermediate B. In the next step, oxygen molecules participated in the process and radical (C) was formed *via* the homolysis of the C–Cu bond.^37,39^ The generation of C from B was achieved *via* a radical species (for detailed process, see ESI, Scheme S1†). Then, the 1,3-hydrogen migration and homolysis of the O–O bond generated intermediate E, which could be detected in the system. A combination of [Cu(ii)]/L and transfer of electrons afforded G. Finally, another oxygen molecule was inserted, and product 2a was formed after intramolecular electron transfer, releasing CO at the same time.^38^

## 3. Conclusions

Summarily, a homogeneous Cu(ii) catalytical system was set up for the preparation of cyclic imides from alkene-tethered amides. In this environmentally friendly reaction pathway, no expensive catalyst was employed. O_2_ was used as an efficient and easily available terminal oxidant. Products containing succinimide and glutarimide structures can find wide applications in pharmaceutical syntheses along with successfully scaled-up experiments. Finally, a possible reaction pathway was proposed and verified by control experiments and isotope-labeling experiments. We believe that our catalytic system has academic and practical worth, and further investigations on the cascade amidoarylation of unactivated olefins catalyzed by copper complexes are ongoing.

## 4. Experimental

### 4.1 Materials

Metal salts, ligands, additives, raw materials, and solvents were purchased from Sinopharm Chemical Reagent Co, J&K Chemicals, or Sigma-Aldrich. Materials obtained from commercial resources were used without further purification unless otherwise noted.

### 4.2 Instrumentation

Liquid ^1^H NMR spectra were obtained in CDCl_3_ using the residual CHCl_3_ as the internal reference (7.26 ppm) using a Bruker 400 spectrometer. ^1^H NMR peaks were labeled as singlet (s), doublet (d), triplet (t), quartet (q), and multiplet (m). The coupling constant values were reported in Hertz (Hz). Liquid ^13^C NMR spectra were conducted at 100 MHz in CDCl_3_ using residual CHCl_3_ as the internal reference (77.0 ppm). GC-MS analysis was performed using gas chromatography-mass spectrometry (GC-MS, 7890A and 5975C, Agilent). High-resolution electrospray ionization mass spectrometry (HR-ESI-MS) was performed on a Bruker FT-ICR-MS instrument (Solarix 9.4T).

### 4.3 Synthesis of substrates

Taking the synthesis of 2-ethyl-*N*-ethyoxy-2-phenylpent-4-enamide (1a) as the example, the procedure was as follows. According to the reported processes,^41,46^ freshly made lithium diisopropylamide (LDA, 12.5 mmol) was mixed with a pre-prepared solution of 2-phenylbutanoic acid (10 mmol) in 10 mL extra dry THF at 0 and 40 °C and maintained for 2 h. After that, allyl bromide (18 mmol) was added dropwise and the solution was stirred for 2.5 h. Liquid separation after dilution (diethyl ether/water), extraction after acidification (water phase), and column chromatography on the silica gel afforded 2-ethyl-2-phenylpent-4-enoic acid for use in the subsequent step. Oxalyl chloride (12.5 mmol) was added dropwise into a solution of 2-ethyl-2-phenylpent-4-enoic acid (10 mmol) in CH_2_Cl_2_ (10 mL) followed by adding a catalytic amount of DMF. After stirring for 2 h, the solvent was removed *via* a rotary evaporator. The remnant solid was slowly added to a mixture of EtONH_2_·HCl (15 mmol) and K_2_CO_3_ (20 mmol) in EtOAc and H_2_O (2 : 1). After another 2 h, the organic phase was collected and the target compound in the aqueous phase was extracted by EtOAc. Washing, desiccation, filtration, and column chromatography afforded the target compound of 2-ethyl-*N*-ethyoxy-2-phenylpent-4-enamide (1a).

### 4.4 General procedure for the reaction of alkene-tethered amides to cyclic imides

Substrate (0.1 mmol), copper salts (0.01 mmol), ligand (0.015 mmol), and solvent (1.5 mL) were successively loaded into a reactor; then, the reactor was connected to an O_2_ balloon. Next, the reactor was moved into an oil bath maintained at the desired temperature (*e.g.*, 100 °C) and stirred for 24 h. After this reaction, the reactor was cooled down to room temperature. The products were isolated by column chromatography on silica gel using *n*-hexane/ethyl acetate as the eluent and their NMR and MS spectra were obtained.

### 4.5 Characterization data of substrates, products, and reaction intermediates

#### *N*-Ethoxy-2-ethyl-2-phenylpent-4-enamide (1a)

^1^H NMR (400 MHz, chloroform-*d*) *δ* 7.39–7.30 (m, 4H), 7.29–7.22 (m, 1H), 7.20 (s, 1H), 5.78 (m, 1H), 5.24–5.17 (m, 1H), 5.17–5.12 (m, 1H), 3.72 (q, *J* = 8.0 Hz, 2H), 2.73–2.62 (m, 2H), 1.91 (q, *J* = 7.9 Hz, 2H), 1.18 (t, *J* = 8.0 Hz, 3H), 0.87 (t, *J* = 8.0 Hz, 3H); ^13^C {^1^H} NMR (100 MHz, chloroform-*d*) *δ* 174.00, 141.54, 132.73, 128.59, 127.40, 126.84, 118.10, 67.96, 51.12, 41.63, 29.38, 12.96, 8.90; HRMS (ESI) *m*/*z* calcd for C_15_H_22_NO_2_ [M + H]^+^ 248.1645, found 248.1648.

#### *N*-Ethoxy-2,2-dimethylpent-4-enamide (1b)

^1^H NMR (400 MHz, chloroform-*d*) *δ* 7.48 (s, 1H), 5.78 (m, 1H), 5.16 (m, 2H), 3.72 (q, *J* = 8.0 Hz, 2H), 2.31 (m, 2H), 1.20 (t, *J* = 8.0 Hz, 3H), 1.11 (s, 6H); ^13^C {^1^H} NMR (100 MHz, chloroform-*d*) *δ* 176.75, 134.30, 118.04, 67.92, 44.68, 42.56, 25.10, 12.95; HRMS (ESI) *m*/*z* calcd for C_9_H_18_NO_2_ [M + H]^+^ 172.1332, found 172.1327.

#### *N*-Ethoxy-2,2-diethylpent-4-enamide (1c)

^1^H NMR (400 MHz, chloroform-*d*) *δ* 7.45 (s, 1H), 5.77 (m, 1H), 5.15 (m, 2H), 3.72 (q, *J* = 8.0 Hz, 2H), 2.32 (m, 2H), 1.71 (m, 4H), 1.19 (t, *J* = 8.0 Hz, 3H), 0.87 (t, *J* = 8.0 Hz, 6H); ^13^C {^1^H} NMR (100 MHz, chloroform-*d*) *δ* 175.99, 134.68, 118.15, 67.96, 49.62, 41.30, 28.04, 12.95, 8.66; HRMS (ESI) *m*/*z* calcd for C_11_H_22_NO_2_ [M + H]^+^ 200.1645, found 200.1651.

#### *N*-Ethoxy-2-ethyl-2-methylpent-4-enamide (1d)

^1^H NMR (400 MHz, chloroform-*d*) *δ* 7.47 (s, 1H), 5.77 (m, 1H), 5.15 (m, 2H), 3.72 (q, *J* = 8.0 Hz, 2H), 2.32 (m, 1H), 2.25 (m, 1H), 1.63 (q, *J* = 7.9 Hz, 2H), 1.20 (t, *J* = 8.0 Hz, 3H), 1.15 (s, 3H), 0.89 (t, *J* = 8.0 Hz, 3H); ^13^C {^1^H} NMR (100 MHz, chloroform-*d*) *δ* 175.02, 134.92, 118.03, 67.89, 46.68, 42.40, 29.25, 20.35, 12.95, 9.07; HRMS (ESI) *m*/*z* calcd for C_10_H_20_NO_2_ [M + H]^+^ 186.1489, found 186.1483.

#### *N*-Ethoxy-2-methyl-2-phenylpent-4-enamide (1e)

^1^H NMR (400 MHz, chloroform-*d*) *δ* 7.39–7.31 (m, 2H), 7.31–7.23 (m, 3H), 7.22 (s, 1H), 5.78 (m, 1H), 5.18 (m, 2H), 3.72 (q, *J* = 8.0 Hz, 2H), 2.77–2.65 (m, 2H), 1.45 (s, 3H), 1.18 (t, *J* = 8.0 Hz, 3H); ^13^C {^1^H} NMR (100 MHz, chloroform-*d*) *δ* 173.43, 141.28, 132.81, 128.46, 127.63, 126.55, 118.22, 67.96, 47.46, 41.63, 23.47, 12.96; HRMS (ESI) *m*/*z* calcd for C_14_H_20_NO_2_ [M + H]^+^ 234.1489, found 234.1492.

#### *N*-Ethoxy-2,3-diethyl-2-phenylpent-4-enamide (1f)

^1^H NMR (400 MHz, chloroform-*d*) *δ* 7.47 (s, 1H), 7.38–7.23 (m, 5H), 5.80 (m, 1H), 5.25–5.15 (m, 2H), 3.72 (q, *J* = 8.0 Hz, 2H), 2.86 (m, 1H), 2.01 (q, *J* = 8.0 Hz, 2H), 1.39–1.26 (m, 2H), 1.18 (t, *J* = 8.0 Hz, 3H), 0.90 (m, 6H); ^13^C {^1^H} NMR (100 MHz, chloroform-*d*) *δ* 175.16, 141.80, 137.59, 128.46, 127.58, 127.35, 117.10, 67.96, 57.83, 48.83, 28.95, 24.81, 12.95, 11.65, 8.82; HRMS (ESI) *m*/*z* calcd for C_17_H_26_NO_2_ [M + H]^+^ 276.1958, found 276.1955.

#### *N*-Ethoxy-2,2-diphenylpent-4-enamide (1g)

^1^H NMR (400 MHz, chloroform-*d*) *δ* 7.39–7.32 (m, 4H), 7.32–7.24 (m, 6H), 6.96 (s, 1H), 5.89 (m, 1H), 5.24 (m, 1H), 5.17 (m, 1H), 3.72 (q, *J* = 8.0 Hz, 2H), 2.75 (m, 2H), 1.17 (t, *J* = 8.0 Hz, 3H); ^13^C {^1^H} NMR (100 MHz, chloroform-*d*) *δ* 173.14, 141.72, 132.24, 128.24, 128.06, 127.64, 118.56, 67.96, 58.67, 44.32, 12.95; HRMS (ESI) *m*/*z* calcd for C_19_H_22_NO_2_ [M + H]^+^ 296.1645, found 296.1641.

#### 1-Allyl-*N*-ethoxycyclobutane-1-carboxamide (1h)

^1^H NMR (400 MHz, chloroform-*d*) *δ* 7.88 (s, 1H), 5.76 (m, 1H), 5.17 (m, 1H), 5.12 (m, 1H), 3.72 (q, *J* = 8.0 Hz, 2H), 2.28 (m, 2H), 2.04–1.92 (m, 4H), 1.77–1.69 (m, 1H), 1.69–1.59 (m, 1H), 1.20 (t, *J* = 8.0 Hz, 3H); ^13^C {^1^H} NMR (100 MHz, chloroform-*d*) *δ* 173.47, 134.55, 118.14, 67.89, 51.47, 41.38, 30.94, 17.78, 12.95; HRMS (ESI) *m*/*z* calcd for C_10_H_18_NO_2_ [M + H]^+^ 184.1332, found 184.1336.

#### 1-Allyl-*N*-ethoxycyclopentane-1-carboxamide (1i)

^1^H NMR (400 MHz, chloroform-*d*) *δ* 8.07 (s, 1H), 5.76 (m, 1H), 5.16 (m, 1H), 5.11 (m, 1H), 3.72 (q, *J* = 8.0 Hz, 2H), 2.26 (dt, *J* = 6.2, 1.0 Hz, 2H), 1.86–1.74 (m, 6H), 1.72–1.64 (m, 2H), 1.19 (t, *J* = 8.0 Hz, 3H); ^13^C {^1^H} NMR (100 MHz, chloroform-*d*) *δ* 175.14, 134.64, 118.24, 67.96, 54.40, 41.09, 35.01, 23.59, 12.95; HRMS (ESI) *m*/*z* calcd for C_11_H_20_NO_2_ [M + H]^+^ 198.1489, found 198.1493.

#### 1-Allyl-*N*-ethoxycyclohexane-1-carboxamide (1j)

^1^H NMR (400 MHz, chloroform-*d*) *δ* 8.07 (s, 1H), 5.76 (m, 1H), 5.17 (m, 1H), 5.10 (dq, *J* = 13.4, 1.0 Hz, 1H), 3.72 (q, *J* = 8.0 Hz, 2H), 2.25 (m, 2H), 1.79 (t, *J* = 6.8 Hz, 4H), 1.66–1.56 (m, 4H), 1.56–1.46 (m, 2H), 1.19 (t, *J* = 8.0 Hz, 3H); ^13^C {^1^H} NMR (100 MHz, chloroform-*d*) *δ* 175.53, 134.56, 118.24, 67.97, 48.45, 40.96, 33.41, 25.60, 23.25, 12.96; HRMS (ESI) *m*/*z* calcd for C_12_H_22_NO_2_ [M + H]^+^ 212.1645, found 212.1649.

#### *N*-Ethoxy-2-vinylcyclohexane-1-carboxamide (1k)

^1^H NMR (400 MHz, chloroform-*d*) *δ* 8.40 (s, 1H), 5.79 (m, 1H), 5.20 (m, 1H), 5.14 (m, 1H), 3.71 (q, *J* = 8.0 Hz, 2H), 2.77 (m, 1H), 2.57 (q, *J* = 7.0 Hz, 1H), 1.74–1.50 (m, 8H), 1.19 (t, *J* = 8.0 Hz, 3H); ^13^C {^1^H} NMR (100 MHz, chloroform-*d*) *δ* 173.39, 139.80, 115.37, 68.18, 47.38, 39.81, 30.29, 26.60, 24.62, 24.47, 12.95; HRMS (ESI) *m*/*z* calcd for C_11_H_20_NO_2_ [M + H]^+^ 198.1489, found 198.1492.

#### *N*-Ethoxy-2-vinylbenzamide (1l)

^1^H NMR (400 MHz, chloroform-*d*) *δ* 9.55 (s, 1H), 7.75–7.70 (m, 1H), 7.51–7.43 (m, 2H), 7.43–7.38 (m, 1H), 7.12 (m, 1H), 5.69 (m, 1H), 5.55 (m, 1H), 3.73 (q, *J* = 8.0 Hz, 2H), 1.19 (t, *J* = 8.0 Hz, 3H); ^13^C {^1^H} NMR (100 MHz, chloroform-*d*) *δ* 166.56, 135.88, 133.60, 130.72, 130.45, 128.64, 127.45, 127.02, 117.60, 68.02, 12.95; HRMS (ESI) *m*/*z* calcd for C_11_H_14_NO_2_ [M + H]^+^ 192.1019, found 192.1023.

#### 1-Ethoxy-3-ethyl-3-phenylpyrrolidine-2,5-dione (2a)

^1^H NMR (400 MHz, chloroform-*d*) *δ* 7.37–7.29 (m, 4H), 7.29–7.21 (m, 1H), 3.88 (q, *J* = 8.0 Hz, 2H), 3.01–2.91 (m, 2H), 2.25 (m, 1H), 1.75 (m, 1H), 1.20 (t, *J* = 8.0 Hz, 3H), 0.87 (t, *J* = 8.0 Hz, 3H); ^13^C {^1^H} NMR (100 MHz, chloroform-*d*) *δ* 178.38, 169.68, 141.37, 128.72, 127.46, 126.74, 66.92, 49.63, 42.99, 29.04, 13.28, 9.30; HRMS (ESI) *m*/*z* calcd for C_14_H_18_NO_3_ [M + H]^+^ 248.1281, found 248.1273.

#### 1-Ethoxy-3,3-dimethylpyrrolidine-2,5-dione (2b)

^1^H NMR (400 MHz, chloroform-*d*) *δ* 3.88 (q, *J* = 8.0 Hz, 2H), 2.60 (s, 2H), 1.26 (s, 6H), 1.22 (t, *J* = 8.0 Hz, 3H); ^13^C {^1^H} NMR (100 MHz, chloroform-*d*) *δ* 174.79, 170.53, 66.83, 43.03, 40.86, 26.41, 13.31; HRMS (ESI) *m*/*z* calcd for C_8_H_14_NO_3_ [M + H]^+^ 172.0968, found 172.0972.

#### 1-Ethoxy-3,3-diethylpyrrolidine-2,5-dione (2c)

^1^H NMR (400 MHz, chloroform-*d*) *δ* 3.88 (q, *J* = 8.0 Hz, 2H), 2.63 (s, 1H), 2.56 (s, 1H), 1.88–1.70 (m, 4H), 1.21 (t, *J* = 8.0 Hz, 3H), 0.88 (t, *J* = 8.0 Hz, 6H); ^13^C {^1^H} NMR (100 MHz, chloroform-*d*) *δ* 177.06, 171.12, 66.90, 48.68, 40.58, 27.91, 13.30, 8.60; HRMS (ESI) *m*/*z* calcd for C_10_H_18_NO_3_ [M + H]^+^ 200.1281, found 200.1275.

#### 1-Ethoxy-3-ethyl-3-methylpyrrolidine-2,5-dione (2d)

^1^H NMR (400 MHz, chloroform-*d*) *δ* 3.88 (q, *J* = 8.0 Hz, 2H), 2.56 (d, *J* = 12.4 Hz, 1H), 2.51 (d, *J* = 12.4 Hz, 1H), 1.64 (q, *J* = 8.0 Hz, 2H), 1.26–1.18 (m, 6H), 0.88 (t, *J* = 8.0 Hz, 3H); ^13^C {^1^H} NMR (100 MHz, chloroform-*d*) *δ* 175.03, 170.65, 66.84, 43.38, 41.30, 29.19, 20.69, 13.30, 9.18; HRMS (ESI) *m*/*z* calcd for C_9_H_16_NO_3_ [M + H]^+^ 186.1125, found 186.1114.

#### 1-Ethoxy-3-methyl-3-phenylpyrrolidine-2,5-dione (2e)

^1^H NMR (400 MHz, chloroform-*d*) *δ* 7.38–7.31 (m, 2H), 7.31–7.22 (m, 3H), 3.88 (q, *J* = 8.0 Hz, 2H), 2.94 (d, *J* = 1.5 Hz, 2H), 1.58 (s, 3H), 1.21 (t, *J* = 8.0 Hz, 3H); ^13^C {^1^H} NMR (100 MHz, chloroform-*d*) *δ* 174.42, 169.74, 142.30, 128.68, 127.68, 126.04, 66.92, 46.96, 45.20, 24.22, 13.29; HRMS (ESI) *m*/*z* calcd for C_13_H_16_NO_3_ [M + H]^+^ 234.1125, found 234.1133.

#### 1-Ethoxy-3,4-diethyl-3-phenylpyrrolidine-2,5-dione (2f)

^1^H NMR (400 MHz, chloroform-*d*) *δ* 7.37–7.30 (m, 2H), 7.30–7.23 (m, 3H), 3.89 (m, 2H), 3.15 (m, 1H), 2.25–2.14 (m, 1H), 2.14–2.06 (m, 1H), 1.67 (m, 1H), 1.56 (m, 1H), 1.20 (t, *J* = 8.0 Hz, 3H), 1.00 (m, 3H), 0.92 (t, *J* = 8.0 Hz, 3H); ^13^C {^1^H} NMR (100 MHz, chloroform-*d*) *δ* 175.46, 171.98, 139.38, 128.63, 127.66, 126.55, 66.97, 51.64, 46.31, 29.95, 18.55, 13.28, 12.66, 8.76; HRMS (ESI) *m*/*z* calcd for C_16_H_22_NO_3_ [M + H]^+^ 276.1594, found 276.1598.

#### 1-Ethoxy-3,3-diphenylpyrrolidine-2,5-dione (2g)

^1^H NMR (400 MHz, chloroform-*d*) *δ* 7.39–7.32 (m, 4H), 7.32–7.26 (m, 6H), 3.88 (q, *J* = 8.0 Hz, 2H), 3.23 (s, 1H), 3.17 (s, 1H), 1.18 (t, *J* = 8.0 Hz, 3H); ^13^C {^1^H} NMR (100 MHz, chloroform-*d*) *δ* 173.01, 169.19, 139.86, 128.21, 127.58, 126.77, 66.97, 55.51, 44.39, 13.28; HRMS (ESI) *m*/*z* calcd for C_19_H_18_NO_3_ [M + H]^+^ 308.1281, found 308.1287.

#### 6-Ethoxy-6-azaspiro[3.4]octane-5,7-dione (2h)

^1^H NMR (400 MHz, chloroform-*d*) *δ* 3.88 (q, *J* = 8.0 Hz, 2H), 2.63 (s, 2H), 2.19–2.04 (m, 4H), 1.79–1.61 (m, 2H), 1.21 (t, *J* = 8.0 Hz, 3H); ^13^C {^1^H} NMR (100 MHz, chloroform-*d*) *δ* 173.80, 171.10, 66.84, 50.09, 40.05, 31.83, 17.90, 13.29; HRMS (ESI) *m*/*z* calcd for C_9_H_14_NO_3_ [M + H]^+^ 184.0968, found 184.0964.

#### 2-Ethoxy-2-azaspiro[4.4]nonane-1,3-dione (2i)

^1^H NMR (400 MHz, chloroform-*d*) *δ* 3.88 (q, *J* = 8.0 Hz, 2H), 2.61 (s, 2H), 1.88–1.74 (m, 8H), 1.21 (t, *J* = 8.0 Hz, 3H); ^13^C {^1^H} NMR (100 MHz, chloroform-*d*) *δ* 175.09, 170.67, 66.90, 47.93, 42.19, 36.21, 23.27, 13.30; HRMS (ESI) *m*/*z* calcd for C_10_H_16_NO_3_ [M + H]^+^ 198.1125, found 198.1129.

#### 2-Ethoxy-2-azaspiro[4.5]decane-1,3-dione (2j)

^1^H NMR (400 MHz, chloroform-*d*) *δ* 3.88 (q, *J* = 8.0 Hz, 2H), 2.64 (s, 2H), 1.89–1.81 (m, 2H), 1.75 (m, 2H), 1.69–1.55 (m, 4H), 1.55–1.46 (m, 2H), 1.21 (t, *J* = 8.0 Hz, 3H); ^13^C {^1^H} NMR (100 MHz, chloroform-*d*) *δ* 175.02, 170.89, 66.88, 46.23, 40.02, 32.45, 25.74, 23.01, 13.31; HRMS (ESI) *m*/*z* calcd for C_11_H_18_NO_3_ [M + H]^+^ 212.1281, found 212.1286.

#### 2-Ethoxyhexahydro-1*H*-isoindole-1,3(2*H*)-dione (2k)

^1^H NMR (400 MHz, chloroform-*d*) *δ* 3.88 (m, 2H), 2.87–2.79 (m, 2H), 1.71–1.53 (m, 6H), 1.53–1.41 (m, 2H), 1.21 (t, *J* = 8.0 Hz, 3H); ^13^C {^1^H} NMR (100 MHz, chloroform-*d*) *δ* 171.99, 67.18, 40.06, 24.98, 24.71, 13.30; HRMS (ESI) *m*/*z* calcd for C_10_H_16_NO_3_ [M + H]^+^ 198.1125, found 198.1129.

#### 2-Ethoxyisoindoline-1,3-dione (2l)

^1^H NMR (400 MHz, chloroform-*d*) *δ* 7.88–7.83 (m, 2H), 7.79 (m, 2H), 3.99 (q, *J* = 8.0 Hz, 2H), 1.20 (t, *J* = 8.0 Hz, 3H); ^13^C {^1^H} NMR (100 MHz, chloroform-*d*) *δ* 164.75, 133.16, 129.67, 123.31, 70.59, 12.86; HRMS (ESI) *m*/*z* calcd for C_10_H_10_NO_3_ [M + H]^+^ 192.0655, found 1920647.

#### *N*-Ethoxy-2-ethyl-2-phenylhex-5-enamide (3a)

^1^H NMR (400 MHz, chloroform-*d*) *δ* 7.40–7.33 (m, 3H), 7.33–7.29 (m, 1H), 7.29–7.23 (m, 2H), 5.76 (m, 1H), 5.14–5.06 (m, 2H), 3.72 (q, *J* = 8.0 Hz, 2H), 2.28–2.12 (m, 2H), 2.01–1.92 (m, 1H), 1.92–1.81 (m, 3H), 1.18 (t, *J* = 8.0 Hz, 3H), 0.86 (t, *J* = 8.0 Hz, 3H); ^13^C {^1^H} NMR (100 MHz, chloroform-*d*) *δ* 173.43, 142.46, 137.88, 128.58, 127.35, 126.63, 114.75, 67.96, 51.20, 35.75, 30.36, 29.37, 12.96, 8.89; HRMS (ESI) *m*/*z* calcd for C_16_H_24_NO_2_ [M + H]^+^ 262.1802, found 262.1799.

#### *N*-Ethoxy-2,2-diethylhex-5-enamide (3b)

^1^H NMR (400 MHz, chloroform-*d*) *δ* 7.52 (s, 1H), 5.73 (m, 1H), 5.05 (m, 2H), 3.72 (q, *J* = 8.0 Hz, 2H), 2.11 (m, 2H), 1.80–1.63 (m, 5H), 1.59 (m, 1H), 1.19 (t, *J* = 8.0 Hz, 3H), 0.87 (t, *J* = 8.0 Hz, 6H); ^13^C {^1^H} NMR (100 MHz, chloroform-*d*) *δ* 175.54, 137.73, 114.74, 67.97, 48.96, 33.43, 29.67, 28.32, 12.96, 8.61; HRMS (ESI) *m*/*z* calcd for C_12_H_24_NO_2_ [M + H]^+^ 214.1802, found 214.1806.

#### *N*-Ethoxy-2-methyl-2-phenylhex-5-enamide (3c)

^1^H NMR (400 MHz, chloroform-*d*) *δ* 7.39–7.32 (m, 2H), 7.31–7.23 (m, 4H), 5.75 (m, 1H), 5.14–5.06 (m, 2H), 3.72 (q, *J* = 8.0 Hz, 2H), 2.28–2.14 (m, 2H), 2.06 (m, 1H), 2.01–1.94 (m, 1H), 1.47 (s, 3H), 1.18 (t, *J* = 8.0 Hz, 3H); ^13^C {^1^H} NMR (100 MHz, chloroform-*d*) *δ* 173.74, 141.93, 137.77, 128.47, 127.66, 126.74, 114.75, 67.96, 47.50, 37.50, 28.89, 24.06, 12.96; HRMS (ESI) *m*/*z* calcd for C_15_H_22_NO_2_ [M + H]^+^ 248.1645, found 248.1642.

#### *N*-Ethoxy-2,2-diphenylhex-5-enamide (3d)

^1^H NMR (400 MHz, chloroform-*d*) *δ* 7.40–7.33 (m, 4H), 7.33–7.26 (m, 2H), 7.26–7.20 (m, 4H), 7.03 (s, 1H), 5.85–5.73 (m, 1H), 5.09 (dt, *J* = 13.4, 1.0 Hz, 2H), 3.72 (q, *J* = 8.0 Hz, 2H), 2.35–2.28 (m, 2H), 2.28–2.21 (m, 2H), 1.16 (t, *J* = 8.0 Hz, 3H); ^13^C {^1^H} NMR (100 MHz, chloroform-*d*) *δ* 172.56, 142.26, 138.04, 128.12, 127.81, 127.55, 114.75, 67.96, 57.68, 38.65, 29.59, 12.95; HRMS (ESI) *m*/*z* calcd for C_20_H_24_NO_2_ [M + H]^+^ 310.1802, found 310.1806.

#### 1-(But-3-en-1-yl)-*N*-ethoxycyclobutane-1-carboxamide (3e)

^1^H NMR (400 MHz, chloroform-*d*) *δ* 7.95 (s, 1H), 5.73 (m, 1H), 5.05 (dt, *J* = 13.5, 1.1 Hz, 2H), 3.72 (q, *J* = 8.0 Hz, 2H), 2.16–1.96 (m, 6H), 1.78–1.62 (m, 2H), 1.59 (t, *J* = 7.1 Hz, 2H), 1.19 (t, *J* = 8.0 Hz, 3H); ^13^C {^1^H} NMR (100 MHz, chloroform-*d*) *δ* 173.78, 137.79, 114.73, 67.96, 51.08, 33.98, 31.25, 29.62, 17.14, 12.95; HRMS (ESI) *m*/*z* calcd for C_11_H_20_NO_2_ [M + H]^+^ 198.1489, found 198.1485.

#### 1-(But-3-en-1-yl)-*N*-ethoxycyclopentane-1-carboxamide (3f)

^1^H NMR (400 MHz, chloroform-*d*) *δ* 8.14 (s, 1H), 5.73 (m, 1H), 5.04 (dt, *J* = 13.4, 1.0 Hz, 2H), 3.72 (q, *J* = 8.0 Hz, 2H), 2.12 (m, 2H), 1.93–1.85 (m, 2H), 1.85–1.79 (m, 1H), 1.79–1.68 (m, 5H), 1.58 (t, *J* = 7.1 Hz, 2H), 1.19 (t, *J* = 8.0 Hz, 3H); ^13^C {^1^H} NMR (100 MHz, chloroform-*d*) *δ* 176.04, 137.73, 114.74, 67.97, 54.40, 35.77, 35.27, 29.79, 23.64, 12.96; HRMS (ESI) *m*/*z* calcd for C_12_H_22_NO_2_ [M + H]^+^ 212.1645, found 212.1643.

#### 1-(But-3-en-1-yl)-*N*-ethoxycyclohexane-1-carboxamide (3g)

^1^H NMR (400 MHz, chloroform-*d*) *δ* 8.14 (s, 1H), 5.73 (m, 1H), 5.06 (dt, *J* = 13.4, 1.0 Hz, 2H), 3.72 (q, *J* = 8.0 Hz, 2H), 2.11 (m, 2H), 1.82–1.70 (m, 4H), 1.65–1.47 (m, 8H), 1.19 (t, *J* = 8.0 Hz, 3H); ^13^C {^1^H} NMR (100 MHz, chloroform-*d*) *δ* 176.60, 137.82, 114.74, 67.97, 48.19, 34.60, 33.69, 29.83, 25.70, 23.31, 12.96; HRMS (ESI) *m*/*z* calcd for C_13_H_24_NO_2_ [M + H]^+^ 226.1802, found 226.1807.

#### 2-Allyl-*N*-ethoxybenzamide (3h)

^1^H NMR (400 MHz, chloroform-*d*) *δ* 8.35 (s, 1H), 7.76 (dd, *J* = 7.4, 1.5 Hz, 1H), 7.46 (m, 1H), 7.40–7.31 (m, 2H), 5.82 (m, 1H), 5.11 (m, 1H), 5.01 (m, 1H), 3.74 (q, *J* = 8.0 Hz, 2H), 3.47 (dq, *J* = 6.2, 1.0 Hz, 2H), 1.19 (t, *J* = 8.0 Hz, 3H); ^13^C {^1^H} NMR (100 MHz, chloroform-*d*) *δ* 165.58, 136.70, 136.54, 132.86, 130.08, 129.51, 128.89, 127.57, 116.11, 68.05, 36.36, 12.95; HRMS (ESI) *m*/*z* calcd for C_12_H_16_NO_2_ [M + H]^+^ 206.1176, found 206.1174.

#### 1-Ethoxy-3-ethyl-3-phenylpiperidine-2,6-dione (4a)

^1^H NMR (400 MHz, chloroform-*d*) *δ* 7.39–7.32 (m, 2H), 7.30 (t, *J* = 1.6 Hz, 1H), 7.29–7.22 (m, 2H), 3.88 (m, 2H), 2.62–2.50 (m, 2H), 2.11 (dt, *J* = 12.4, 7.1 Hz, 1H), 2.07–1.92 (m, 2H), 1.92–1.85 (m, 1H), 1.18 (t, *J* = 8.0 Hz, 3H), 0.84 (t, *J* = 8.0 Hz, 3H); ^13^C {^1^H} NMR (100 MHz, chloroform-*d*) *δ* 173.73, 167.96, 141.60, 128.59, 127.34, 126.26, 66.92, 47.89, 32.63, 30.37, 30.27, 13.28, 9.45; HRMS (ESI) *m*/*z* calcd for C_15_H_20_NO_3_ [M + H]^+^ 262.1438, found 262.1442.

#### 1-Ethoxy-3,3-diethylpiperidine-2,6-dione (4b)

^1^H NMR (400 MHz, chloroform-*d*) *δ* 3.88 (q, *J* = 8.0 Hz, 2H), 2.49 (t, *J* = 7.1 Hz, 2H), 2.02 (t, *J* = 7.1 Hz, 2H), 1.83–1.68 (m, 4H), 1.21 (t, *J* = 8.0 Hz, 3H), 0.86 (t, *J* = 8.0 Hz, 6H); ^13^C {^1^H} NMR (100 MHz, chloroform-*d*) *δ* 174.37, 167.42, 66.88, 45.25, 31.55, 30.46, 27.87, 13.31, 8.50; HRMS (ESI) *m*/*z* calcd for C_11_H_20_NO_3_ [M + H]^+^ 214.1438, found 214.1435.

#### 1-Ethoxy-3-methyl-3-phenylpiperidine-2,6-dione (4c)

^1^H NMR (400 MHz, chloroform-*d*) *δ* 7.38–7.31 (m, 2H), 7.29–7.21 (m, 3H), 3.88 (m, 2H), 2.62–2.49 (m, 2H), 2.21–2.06 (m, 2H), 1.52 (s, 3H), 1.18 (t, *J* = 8.0 Hz, 3H); ^13^C {^1^H} NMR (100 MHz, chloroform-*d*) *δ* 172.40, 167.97, 142.32, 128.47, 127.66, 126.21, 66.92, 45.32, 33.70, 29.90, 24.64, 13.28; HRMS (ESI) *m*/*z* calcd for C_14_H_18_NO_3_ [M + H]^+^ 248.1281, found 248.1279.

#### 1-Ethoxy-3,3-diphenylpiperidine-2,6-dione (4d)

^1^H NMR (400 MHz, chloroform-*d*) *δ* 7.39–7.32 (m, 4H), 7.32–7.25 (m, 2H), 7.25–7.20 (m, 4H), 3.88 (q, *J* = 8.0 Hz, 2H), 2.58–2.52 (m, 2H), 2.27 (m, 2H), 1.15 (t, *J* = 8.0 Hz, 3H); ^13^C {^1^H} NMR (100 MHz, chloroform-*d*) *δ* 169.72, 167.94, 141.45, 128.14, 127.55, 126.72, 66.99, 55.54, 35.03, 29.72, 13.28; HRMS (ESI) *m*/*z* calcd for C_19_H_20_NO_3_ [M + H]^+^ 310.1438, found 310.1435.

#### 6-Ethoxy-6-azaspiro[3.5]nonane-5,7-dione (4e)

^1^H NMR (400 MHz, chloroform-*d*) *δ* 3.88 (q, *J* = 8.0 Hz, 2H), 2.51 (t, *J* = 7.1 Hz, 2H), 2.15–2.11 (m, 1H), 2.09 (d, *J* = 7.1 Hz, 1H), 2.05 (d, *J* = 7.1 Hz, 1H), 2.04–2.01 (m, 1H), 1.99 (t, *J* = 7.1 Hz, 2H), 1.68 (q, *J* = 7.2 Hz, 2H), 1.21 (t, *J* = 8.0 Hz, 3H); ^13^C {^1^H} NMR (100 MHz, chloroform-*d*) *δ* 171.71, 167.42, 66.90, 45.69, 32.39, 30.73, 29.81, 17.28, 13.30; HRMS (ESI) *m*/*z* calcd for C_10_H_16_NO_3_ [M + H]^+^ 198.1125, found 198.1129.

#### 7-Ethoxy-7-azaspiro[4.5]decane-6,8-dione (4f)

^1^H NMR (400 MHz, chloroform-*d*) *δ* 3.88 (q, *J* = 8.0 Hz, 2H), 2.50 (t, *J* = 7.1 Hz, 2H), 2.01–1.86 (m, 4H), 1.82–1.74 (m, 4H), 1.74–1.63 (m, 2H), 1.21 (t, *J* = 8.0 Hz, 3H); ^13^C {^1^H} NMR (100 MHz, chloroform-*d*) *δ* 172.79, 167.42, 66.88, 50.52, 33.73, 33.33, 30.40, 23.49, 13.31; HRMS (ESI) *m*/*z* calcd for C_11_H_18_NO_3_ [M + H]^+^ 212.1281, found 212.1284.

#### 2-Ethoxy-2-azaspiro[5.5]undecane-1,3-dione (4g)

^1^H NMR (400 MHz, chloroform-*d*) *δ* 3.88 (q, *J* = 8.0 Hz, 2H), 2.50 (t, *J* = 7.1 Hz, 2H), 1.97 (t, *J* = 7.1 Hz, 2H), 1.86–1.73 (m, 4H), 1.66–1.47 (m, 6H), 1.21 (t, *J* = 8.0 Hz, 3H); ^13^C {^1^H} NMR (100 MHz, chloroform-*d*) *δ* 173.99, 168.03, 66.92, 42.92, 32.89, 32.23, 30.40, 25.74, 22.91, 13.29; HRMS (ESI) *m*/*z* calcd for C_12_H_20_NO_3_ [M + H]^+^ 226.1438, found 226.1443.

#### 2-Ethoxyisoquinoline-1,3(2*H*,4*H*)-dione (4h)

^1^H NMR (400 MHz, chloroform-*d*) *δ* 7.62 (m, 1H), 7.51 (m, 1H), 7.43 (m, 1H), 7.40–7.35 (m, 1H), 3.89 (q, *J* = 8.0 Hz, 2H), 3.44 (d, *J* = 1.0 Hz, 2H), 1.21 (t, *J* = 8.0 Hz, 3H); ^13^C {^1^H} NMR (100 MHz, chloroform-*d*) *δ* 172.53, 162.88, 135.20, 131.03, 129.81, 127.44, 126.49, 125.53, 67.01, 35.82, 13.31; HRMS (ESI) *m*/*z* calcd for C_11_H_12_NO_3_ [M + H]^+^ 206.0812, found 206.0809.

#### 1-Ethoxy-4-ethyl-5-oxo-4-phenylpyrrolidine-2-carbaldehyde (intermediate E)

^1^H NMR (400 MHz, chloroform-*d*) *δ* 9.77 (d, *J* = 6.2 Hz, 1H), 7.38–7.32 (m, 2H), 7.31–7.22 (m, 3H), 4.19 (td, *J* = 7.0, 6.2 Hz, 1H), 3.78 (q, *J* = 8.0 Hz, 2H), 2.53 (dd, *J* = 12.4, 7.0 Hz, 1H), 2.47 (dd, *J* = 12.4, 7.0 Hz, 1H), 2.01–1.84 (m, 2H), 1.20 (t, *J* = 8.0 Hz, 3H), 0.85 (t, *J* = 8.0 Hz, 3H); ^13^C {^1^H} NMR (100 MHz, chloroform-*d*) *δ* 197.34, 174.65, 143.08, 128.59, 127.31, 126.28, 65.63, 65.01, 50.54, 40.23, 29.78, 13.28, 9.45; HRMS (ESI) *m*/*z* calcd for C_15_H_20_NO_3_ [M + H]^+^ 262.1438, found 262.1443.

## Conflicts of interest

There are no conflicts to declare.

## Supplementary Material

RA-010-C9RA10422D-s001
