# Supplementary material for: Preparation of cyclic imides from alkene-tethered amides: application of homogeneous Cu(ii) catalytic systems
Source: RSC Adv. 2020 Feb 21;10(13):7698–707. doi: 10.1039/c9ra10422d (PMC9049870; doi:10.1039/c9ra10422d)

Electronic Supplementary Material (ESI) for RSC Advances.
This journal is © The Royal Society of Chemistry 2020

Electronic Supplementary Information

Preparation of Cyclic Imides from Alkene-tethered Amides: an Application of Homogeneous Cu(II) Catalytical System

Zhenghui Liu[a,*], Peng Wang[b,c], Hualin Ou[b], Zhenzhong Yan[a], Suqing Chen[a], Xingxing Tan[b,d], Dongkun Yu[d], Xinhui Zhao[d], Tiancheng Mu[d,*]

[a] School of Pharmaceutical and Materials Engineering, Taizhou University, Taizhou 318000, Zhejiang, China

[b] Beijing National Laboratory for Molecular Sciences, CAS Research/Education Center for Excellence in Molecular Sciences, Institute of Chemistry, Chinese Academy of Sciences, Beijing 100190, China

[c] Key Laboratory of Green Chemical Media and Reactions, Ministry of Education, School of Chemistry and Chemical Engineering, Henan Normal University, Xinxiang 453007, Henan, China

[d] Department of Chemistry, Renmin University of China, Beijing 100872, China

* Corresponding author.

E-mail address: liuzhenghui@iccas.ac.cn (Z. Liu); tcmu@ruc.edu.cn (T. Mu).

Abstract: A Cu-based homogeneous catalytic system was put forward for the preparation of imides from alkene-tethered amides. O_2 acted as a cheap and easily available oxygen source and a terminal oxidant. Cleavage of C=C bonds and formation of C-N bond were catalyzed by Cu(II) salts with proper nitrogen-containing ligand under 100 °C. The synthetic approach has potential of applying onto pharmaceutical synthesis. Moreover, scaled-up experiment confirmed the practical application ability.

Table of Contents

1.	Reaction condition screening of catalytic system		S3-S15
	Table S1	Ligand information	S3
	Table S2	$FeCl_3 \cdot 6H_2O$	S4
	Table S3	$Co(BF_4)_2 \cdot 6H_2O$	S5
	Table S4	$Ni(acac)_2$	S6
	Table S5	$Cu(acac)_2$	S7
	Table S6	$ZnCl_2$	S8
	Table S7	$MnCl_2 \cdot 4H_2O$	S9
	Table S8	$PdCl_2$	S10
	Table S9	$RuCl_3$	S11
	Table S10	$RhCl_3 \cdot 3H_2O$	S12
	Table S11	$IrCl_3$	S13
	Table S12	Screening of Cu salts	S14
	Table S13	Screening of Cu salts	S14
2.	Studies on the catalytic system		S15-S16
	Table S14	Control experiments	S15
	Table S15	Quantitative data for the kinetic studies	S16
	Scheme S1	Process between intermediate B and C	S16
3.	NMR spectra of substrates, products and intermediate		S17-S57
4.	ESI-MS measurement: instrument parameters and results		S58
	Figure S1	ESI-MS spectra of mechanism research	S59

1. Reaction condition screening of catalytic system

Table S1 Ligand information

Entry	Code	Name
1	A	2,9-dimethyl-1,10-phenanthroline
2	B	2,2'-bipyridine
3	C	2,2'-bipyrimidine
4	D	5*H*-cyclopenta[2,1-*b*:3,4-*b*']dipyridin-5-one
5	E	4-methyl-2-(pyridin-2-yl)-4,5-dihydrooxazole
6	F	(*S*)-4-isopropyl-2-(pyridin-2-yl)-4,5-dihydrooxazole
7	G	2,2'-(propane-2,2-diyl)bis(4,5-dihydrooxazole)
8	H	2,2'-(propane-2,2-diyl)bis(4-isopropyl-4,5-dihydrooxazole)
9	I	N^1,N^1,N^2,N^2-tetramethylethane-1,2-diamine
10	J	N^1,N^1,N^3,N^3-tetramethylpropane-1,3-diamine
11	K	N^1,N^1,N^4,N^4-tetramethylbutane-1,4-diamine
12	L	N^1,N^1,N^5,N^5-tetramethylpentane-1,5-diamine
13	M	triethylamine
14	N	1*H*-imidazole
15	O	(1*E*,2*E*)-N^1,N^2-dicyclohexylethane-1,2-diimine
16	P	7-(tert-butyl)-2,5-dimethyl-3,4-dihydro-2*H*-pyrano[2,3-*b*]quinoline
17	Q	1,2-bis(phenylsulfinyl)ethane
18	R	ethyl(4-methoxyphenyl)sulfane
19	S	triphenylphosphane
20	T	1,2-bis(diphenylphosphanyl)ethane
21	U	2,2'-bis(diphenylphosphanyl)-1,1'-binaphthalene
22	V	(oxybis(2,1-phenylene))bis(diphenylphosphane)

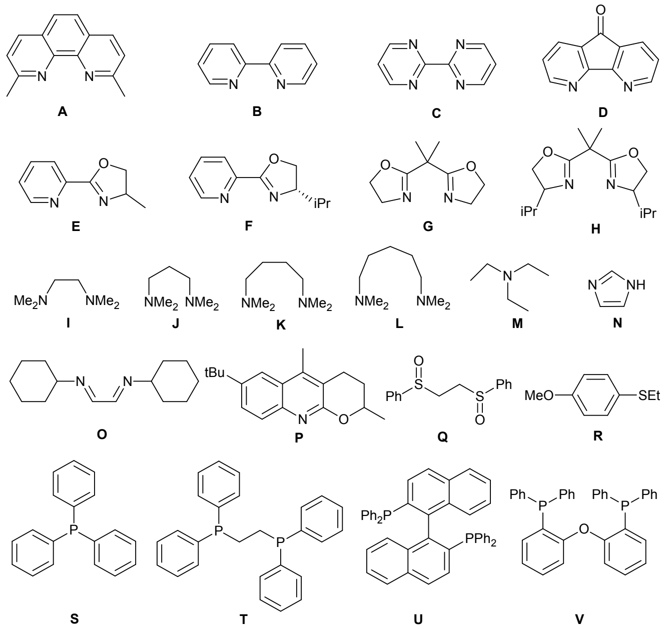

Table S2 Catalytic performance of $FeCl_3 \cdot 6H_2O$: effect of ligands[a]

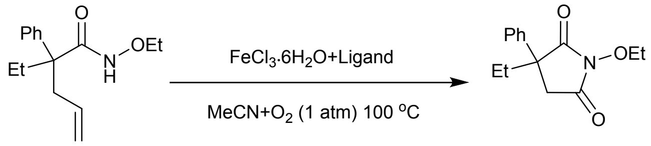

1a **2a**

Entry	Ligand Code	Yield/%[b]
1	A	0.5
2	B	Not detected
3	C	Not detected
4	D	0.2
5	E	0.5
6	F	Not detected
7	G	Not detected
8	H	Not detected
9	I	Not detected
10	J	Not detected
11	K	Not detected
12	L	Not detected
13	M	Not detected
14	N	Not detected
15	O	Not detected
16	P	0.8
17	Q	Not detected
18	R	Not detected
19	S	Not detected
20	T	Not detected
21	U	Not detected
22	V	Not detected

[a] Reaction conditions: **1a**, 0.1 mmol; $FeCl_3 \cdot 6H_2O$, 0.01 mmol (10 mol%); Ligand, 0.015 mmol (15 mol%); MeCN, 1.5 mL; O_2 (1 atm); 100 °C; 24 h.

[b] Determined by ^1H NMR analysis using 1,1,2,2-tetrachloroethane as an internal standard.

Table S3 Catalytic performance of $Co(BF_4)_2·6H_2O$: effect of ligands[a]

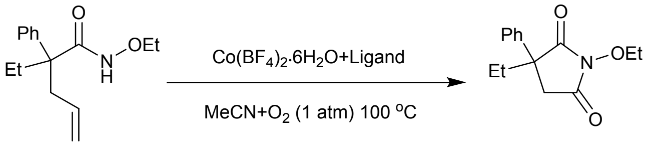

Entry	Ligand Code	Yield/%[b]
1	A	Not detected
2	B	Not detected
3	C	4.8
4	D	3.2
5	E	Not detected
6	F	Not detected
7	G	Not detected
8	H	Not detected
9	I	0.5
10	J	1
11	K	Not detected
12	L	Not detected
13	M	Not detected
14	N	Not detected
15	O	0.8
16	P	1.2
17	Q	Not detected
18	R	Not detected
19	S	Not detected
20	T	Not detected
21	U	Not detected
22	V	Not detected

[a] Reaction conditions: **1a**, 0.1 mmol; $Co(BF_4)_2·6H_2O$, 0.01 mmol (10 mol%); Ligand, 0.015 mmol (15 mol%); MeCN, 1.5 mL; O_2 (1 atm); 100 °C; 24 h.

[b] Determined by ^1H NMR analysis using 1,1,2,2-tetrachloroethane as an internal standard.

Table S4 Catalytic performance of $Ni(acac)_2$: effect of ligands[a]

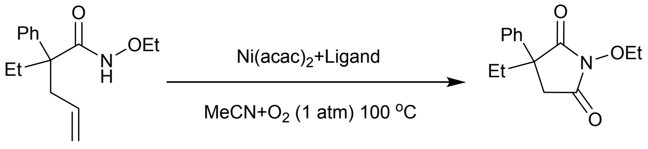

1a　　2a

Entry	Ligand Code	Yield/%[b]
1	A	0.5
2	B	0.4
3	C	0.6
4	D	Not detected
5	E	Not detected
6	F	Not detected
7	G	Not detected
8	H	Not detected
9	I	Not detected
10	J	Not detected
11	K	Not detected
12	L	0.5
13	M	0.8
14	N	0.6
15	O	Not detected
16	P	0.5
17	Q	Not detected
18	R	Not detected
19	S	Not detected
20	T	Not detected
21	U	Not detected
22	V	Not detected

[a] Reaction conditions: **1a**, 0.1 mmol; $Ni(acac)_2$, 0.01 mmol (10 mol%); Ligand, 0.015 mmol (15 mol%); MeCN, 1.5 mL; O_2 (1 atm); 100 °C; 24 h.

[b] Determined by ^1H NMR analysis using 1,1,2,2-tetrachloroethane as an internal standard.

Table S5 Catalytic performance of $Cu(acac)_2$: effect of ligands[a]

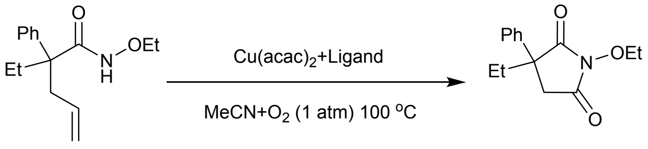

Entry	Ligand Code	Yield/%[b]
1	A	85
2	B	63
3	C	72
4	D	28
5	E	51
6	F	44
7	G	42
8	H	25
9	I	8
10	J	6
11	K	5
12	L	3
13	M	5
14	N	33
15	O	8
16	P	18
17	Q	2
18	R	1
19	S	Not detected
20	T	Not detected
21	U	Not detected
22	V	Not detected

[a] Reaction conditions: **1a**, 0.1 mmol; $Cu(acac)_2$, 0.01 mmol (10 mol%); Ligand, 0.015 mmol (15 mol%); MeCN, 1.5 mL; O_2 (1 atm); 100 °C; 24 h.

[b] Determined by ^1H NMR analysis using 1,1,2,2-tetrachloroethane as an internal standard.

Table S6 Catalytic performance of $ZnCl_2$: effect of ligands[a]

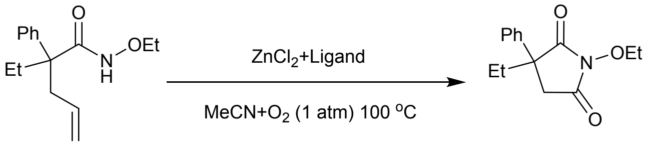

1a **2a**

Entry	Ligand Code	Yield/%[b]
1	A	0.5
2	B	0.5
3	C	0.3
4	D	Not detected
5	E	Not detected
6	F	Not detected
7	G	Not detected
8	H	Not detected
9	I	Not detected
10	J	Not detected
11	K	Not detected
12	L	0.8
13	M	0.4
14	N	0.3
15	O	Not detected
16	P	0.5
17	Q	0.6
18	R	Not detected
19	S	Not detected
20	T	Not detected
21	U	Not detected
22	V	Not detected

[a] Reaction conditions: **1a**, 0.1 mmol; $ZnCl_2$, 0.01 mmol (10 mol%); Ligand, 0.015 mmol (15 mol%); MeCN, 1.5 mL; O_2 (1 atm); 100 °C; 24 h.

[b] Determined by ^1H NMR analysis using 1,1,2,2-tetrachloroethane as an internal standard.

Table S7 Catalytic performance of $MnCl_2·4H_2O$: effect of ligands[a]

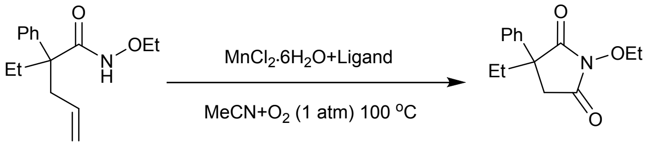

Entry	Ligand Code	Yield/%[b]
1	A	Not detected
2	B	Not detected
3	C	Not detected
4	D	Not detected
5	E	Not detected
6	F	0.4
7	G	0.5
8	H	0.6
9	I	0.3
10	J	Not detected
11	K	Not detected
12	L	Not detected
13	M	Not detected
14	N	Not detected
15	O	Not detected
16	P	Not detected
17	Q	0.3
18	R	0.5
19	S	Not detected
20	T	Not detected
21	U	Not detected
22	V	Not detected

[a] Reaction conditions: **1a**, 0.1 mmol; $MnCl_2·4H_2O$, 0.01 mmol (10 mol%); Ligand, 0.015 mmol (15 mol%); MeCN, 1.5 mL; O_2 (1 atm); 100 °C; 24 h.

[b] Determined by ^1H NMR analysis using 1,1,2,2-tetrachloroethane as an internal standard.

Table S8 Catalytic performance of $PdCl_2$: effect of ligands[a]

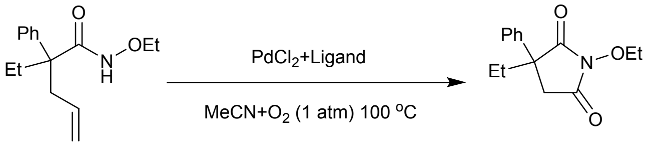

Entry	Ligand Code	Yield/%[b]
1	A	0.5
2	B	0.6
3	C	5.6
4	D	3.2
5	E	Not detected
6	F	Not detected
7	G	Not detected
8	H	Not detected
9	I	Not detected
10	J	Not detected
11	K	Not detected
12	L	1.2
13	M	Not detected
14	N	1
15	O	Not detected
16	P	0.8
17	Q	0.5
18	R	Not detected
19	S	Not detected
20	T	Not detected
21	U	Not detected
22	V	Not detected

[a] Reaction conditions: **1a**, 0.1 mmol; $PdCl_2$, 0.01 mmol (10 mol%); Ligand, 0.015 mmol (15 mol%); MeCN, 1.5 mL; O_2 (1 atm); 100 °C; 24 h.

[b] Determined by ^1H NMR analysis using 1,1,2,2-tetrachloroethane as an internal standard.

Table S9 Catalytic performance of $RuCl_3$: effect of ligands[a]

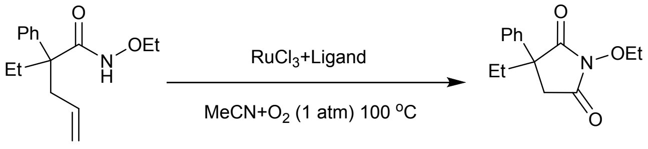

Entry	Ligand Code	Yield/%[b]
1	A	Not detected
2	B	Not detected
3	C	Not detected
4	D	Not detected
5	E	Not detected
6	F	Not detected
7	G	Not detected
8	H	4.8
9	I	Not detected
10	J	1
11	K	Not detected
12	L	Not detected
13	M	Not detected
14	N	0.5
15	O	1.5
16	P	2
17	Q	0.5
18	R	Not detected
19	S	Not detected
20	T	Not detected
21	U	Not detected
22	V	Not detected

[a] Reaction conditions: **1a**, 0.1 mmol; $RuCl_3$, 0.01 mmol (10 mol%); Ligand, 0.015 mmol (15 mol%); MeCN, 1.5 mL; O_2 (1 atm); 100 °C; 24 h.

[b] Determined by ^1H NMR analysis using 1,1,2,2-tetrachloroethane as an internal standard.

Table S10 Catalytic performance of $RhCl_3{\cdot}3H_2O$: effect of ligands[a]

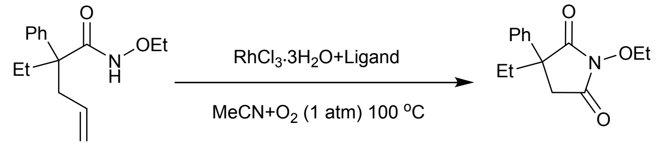

1a **2a**

Entry	Ligand Code	Yield/%[b]
1	A	Not detected
2	B	Not detected
3	C	1
4	D	0.5
5	E	Not detected
6	F	0.6
7	G	5.5
8	H	Not detected
9	I	Not detected
10	J	Not detected
11	K	Not detected
12	L	Not detected
13	M	Not detected
14	N	Not detected
15	O	Not detected
16	P	0.5
17	Q	Not detected
18	R	0.2
19	S	Not detected
20	T	Not detected
21	U	Not detected
22	V	Not detected

[a] Reaction conditions: **1a**, 0.1 mmol; $RhCl_3{\cdot}3H_2O$, 0.01 mmol (10 mol%); Ligand, 0.015 mmol (15 mol%); MeCN, 1.5 mL; O_2 (1 atm); 100 °C; 24 h.

[b] Determined by ^1H NMR analysis using 1,1,2,2-tetrachloroethane as an internal standard.

Table S11 Catalytic performance of $IrCl_3$: effect of ligands[a]

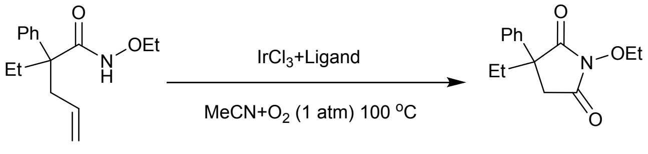

Entry	Ligand Code	Yield/%[b]
1	A	3.2
2	B	0.5
3	C	0.2
4	D	Not detected
5	E	Not detected
6	F	Not detected
7	G	Not detected
8	H	Not detected
9	I	0.5
10	J	Not detected
11	K	1
12	L	Not detected
13	M	0.5
14	N	Not detected
15	O	Not detected
16	P	Not detected
17	Q	Not detected
18	R	Not detected
19	S	Not detected
20	T	Not detected
21	U	Not detected
22	V	Not detected

[a] Reaction conditions: **1a**, 0.1 mmol; $IrCl_3$, 0.01 mmol (10 mol%); Ligand, 0.015 mmol (15 mol%); MeCN, 1.5 mL; O_2 (1 atm); 100 °C; 24 h.

[b] Determined by ^1H NMR analysis using 1,1,2,2-tetrachloroethane as an internal standard.

Table S12 Screening of Cu salts[a]

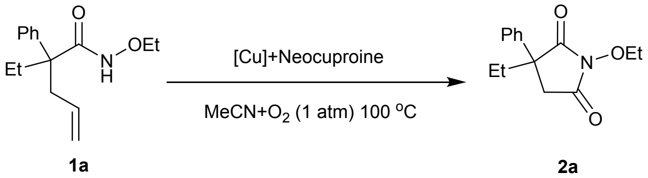

Entry	Cu Salt	Yield/%[b]
1	$CuSO_4 \cdot 5H_2O$	75
2	$Cu(NO_3)_2 \cdot 2.5H_2O$	78
3	$Cu(BF_4)_2 \cdot 2H_2O$	82
4	CuF_2	81
5	$CuCl_2 \cdot 2H_2O$	80
6	$CuBr_2$	77
7	$Cu(acac)_2$	85
8	$CuAc_2 \cdot H_2O$	78

[a] Reaction conditions: **1a**, 0.1 mmol; Cu salts, 0.01 mmol (10 mol%); Neocuproine, 0.015 mmol (15 mol%); MeCN, 1.5 mL; O_2 (1 atm); 100 °C; 24 h.

[b] Determined by ^1H NMR analysis using 1,1,2,2-tetrachloroethane as an internal standard.

Table S13 Screening of Cu salts[a]

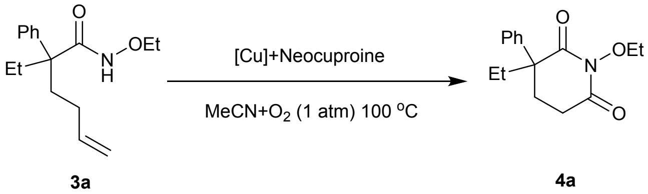

Entry	Cu Salt	Yield/%[b]
1	$CuSO_4 \cdot 5H_2O$	44
2	$Cu(NO_3)_2 \cdot 2.5H_2O$	47
3	$Cu(BF_4)_2 \cdot 2H_2O$	50
4	CuF_2	58
5	$CuCl_2 \cdot 2H_2O$	49
6	$CuBr_2$	45
7	$Cu(acac)_2$	52
8	$CuAc_2 \cdot H_2O$	48

[a] Reaction conditions: **3a**, 0.1 mmol; Cu salts, 0.01 mmol (10 mol%); Neocuproine, 0.015 mmol (15 mol%); MeCN, 1.5 mL; O_2 (1 atm); 100 °C; 24 h.

[b] Determined by ^1H NMR analysis using 1,1,2,2-tetrachloroethane as an internal standard.

2. Studies on the catalytic system

Table S14 Control experiments[a]

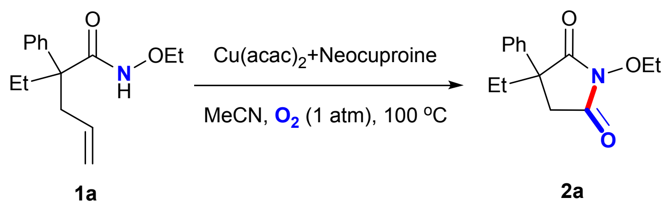

Entry	Deviation from standard conditions	**2a** yield / %[b]
1	No $Cu(acac)_2$	0
2	No neocuproine	0
3	No O_2	0
4	No neocuproine or O_2	0

[a] Stangard conditions: **1a**, 0.1 mmol; $Cu(acac)_2$, 0.01 mmol (10 mol%); ligand neocuproine, 0.015 mmol (15 mol%); MeCN, 1.5 mL; O_2 (1 atm); 100 °C; 24 h. [b] Determined by ^1H NMR analysis using 1,1,2,2-tetrachloroethane as an internal standard.

Table S15 Quantitative data for the kinetic studies[a,b]

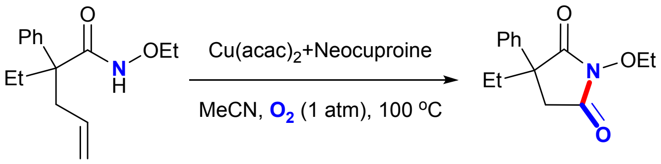

1a **2a**

Entry	Time / h	1a / %	E / %	2a / %
1	0	100	0	0
2	0.5	60	30	8
3	1	40	27	18
4	1.5	35	21	25
5	2	25	18	38
6	3	19	16	49
7	6	17	13	58
8	9	16	10	63
9	12	15	8	68
10	15	12	5	72
11	18	10	3	79
12	21	9	2	82
13	24	8	2	85
14	27	8	2	85

[a] Reaction conditions: 1a, 0.1 mmol; $Cu(acac)_2$, 0.01 mmol (10 mol%); ligand neocuproine, 0.015 mmol (15 mol%); MeCN, 1.5 mL; O_2 (1 atm); 100 ºC. [b] Yields were determined by ^1H NMR analysis using 1,1,2,2-tetrachloroethane as an internal standard.

Scheme S1 Process between intermediate B and C

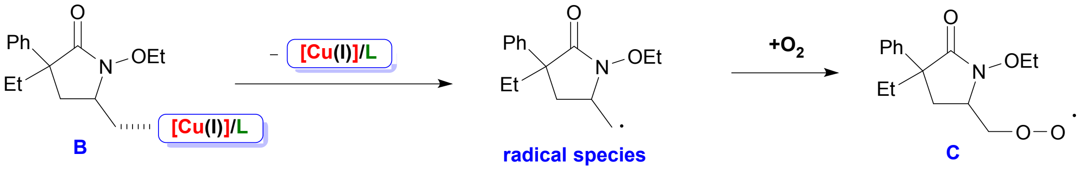

3. NMR spectra of substrates, products and intermediate

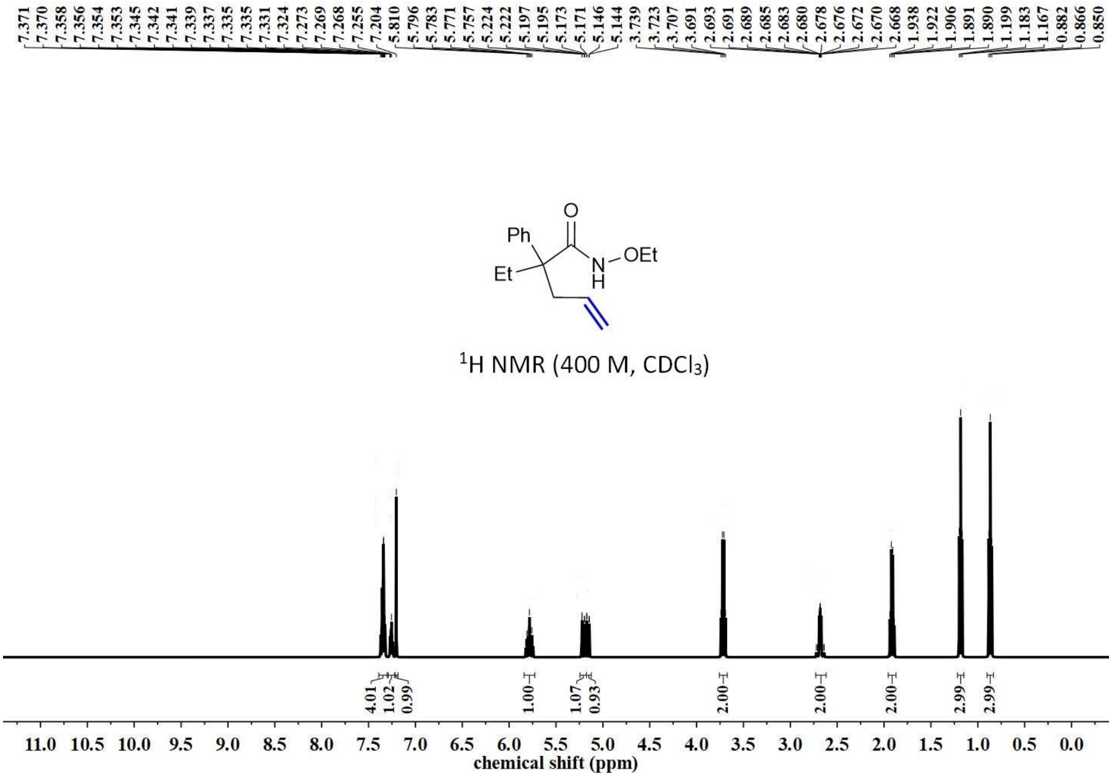

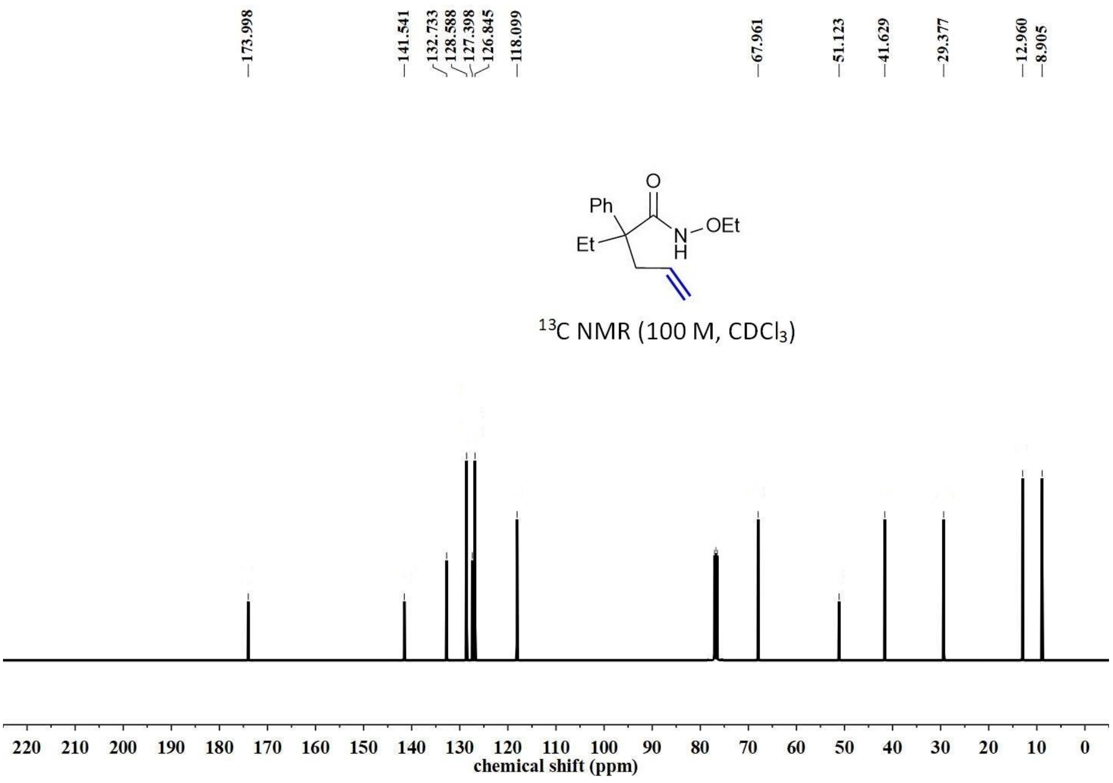

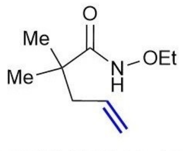

^{1}H NMR (400 M, CDCl$_3$)

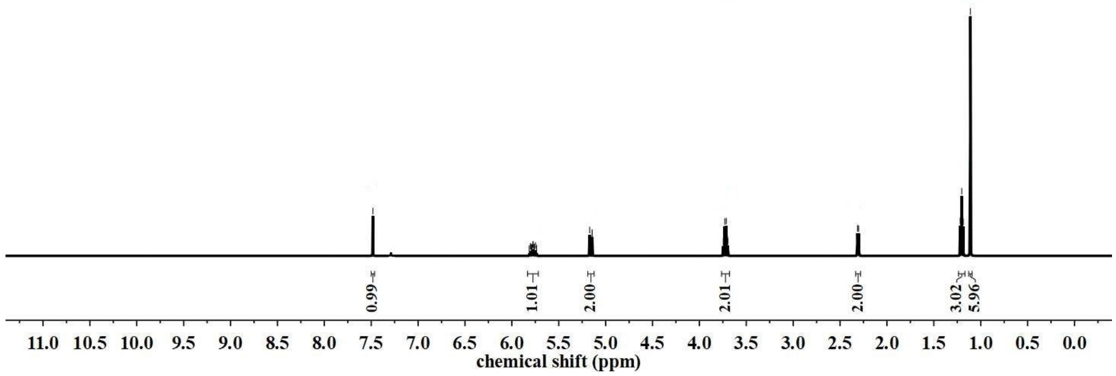

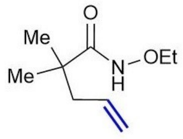

^{13}C NMR (100 M, CDCl$_3$)

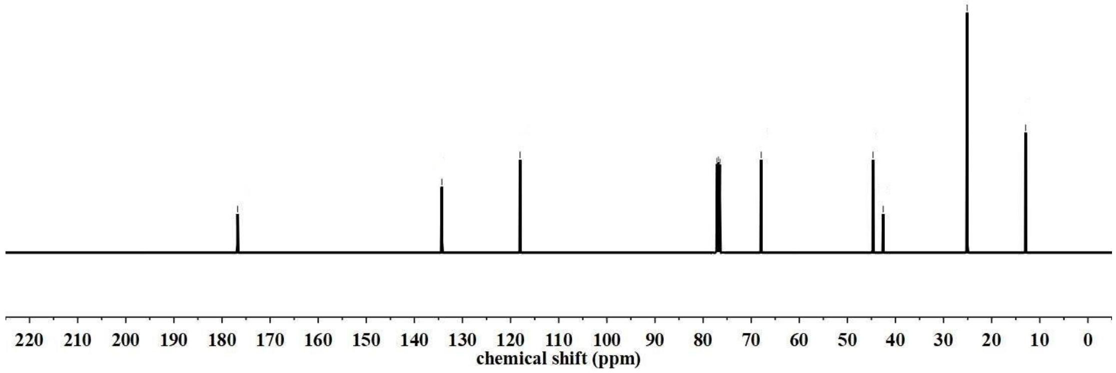

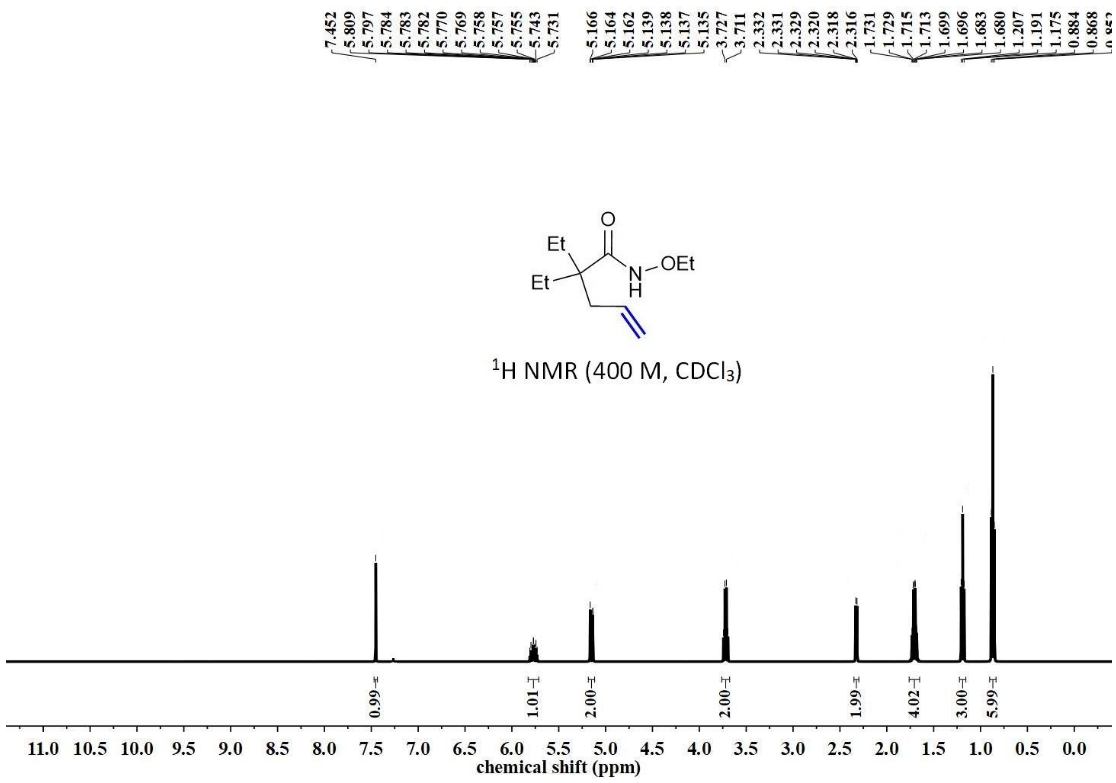
7.452
5.809
5.797
5.784
5.783
5.782
5.770
5.769
5.758
5.757
5.755
5.743
5.731
5.166
5.164
5.162
5.139
5.138
5.137
5.135
3.727
3.711
2.332
2.331
2.329
2.320
2.318
2.316
1.731
1.729
1.715
1.713
1.699
1.696
1.683
1.680
1.207
1.191
1.175
0.884
0.868
O
Et
N
OEt
H
Et
1H NMR (400 M, CDCl3)
0.99
1.01
2.00
2.00
1.99
4.02
3.00
5.99
11.0 10.5 10.0 9.5 9.0 8.5 8.0 7.5 7.0 6.5 6.0 5.5 5.0 4.5 4.0 3.5 3.0 2.5 2.0 1.5 1.0 0.5 0.0
chemical shift (ppm)

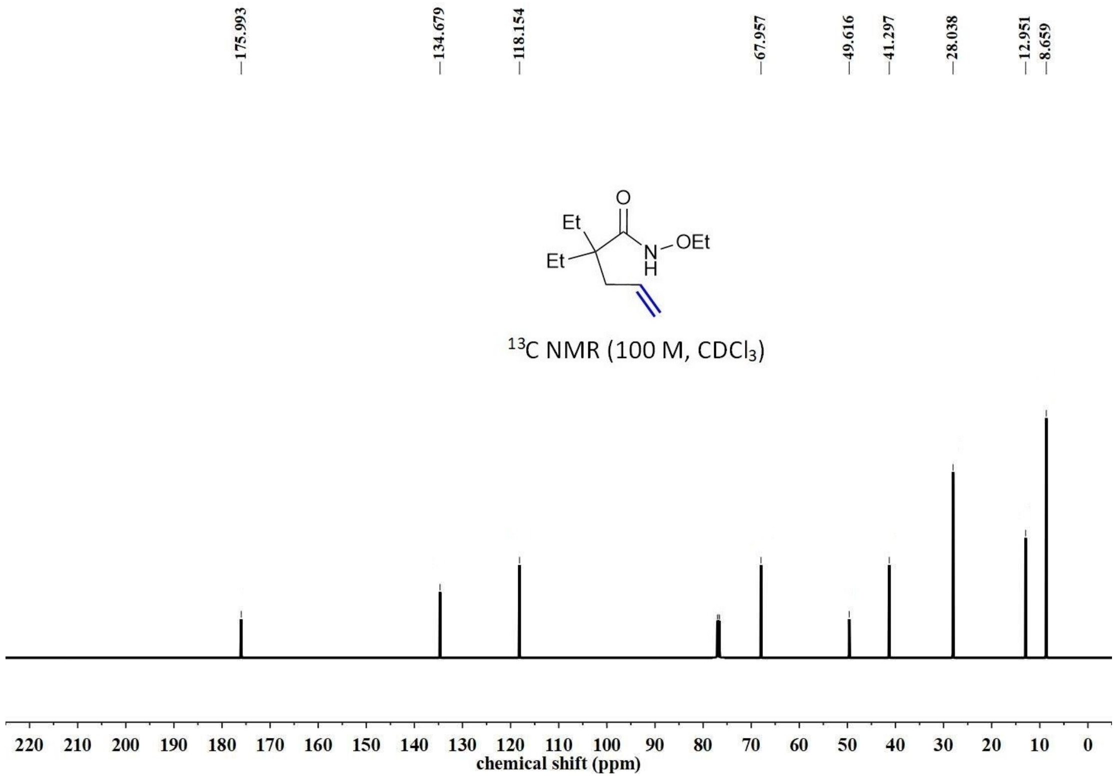
175.993
134.679
118.154
67.957
49.616
41.297
28.038
12.951
8.659
O
Et
N
OEt
H
Et
13C NMR (100 M, CDCl3)
220 210 200 190 180 170 160 150 140 130 120 110 100 90 80 70 60 50 40 30 20 10 0
chemical shift (ppm)

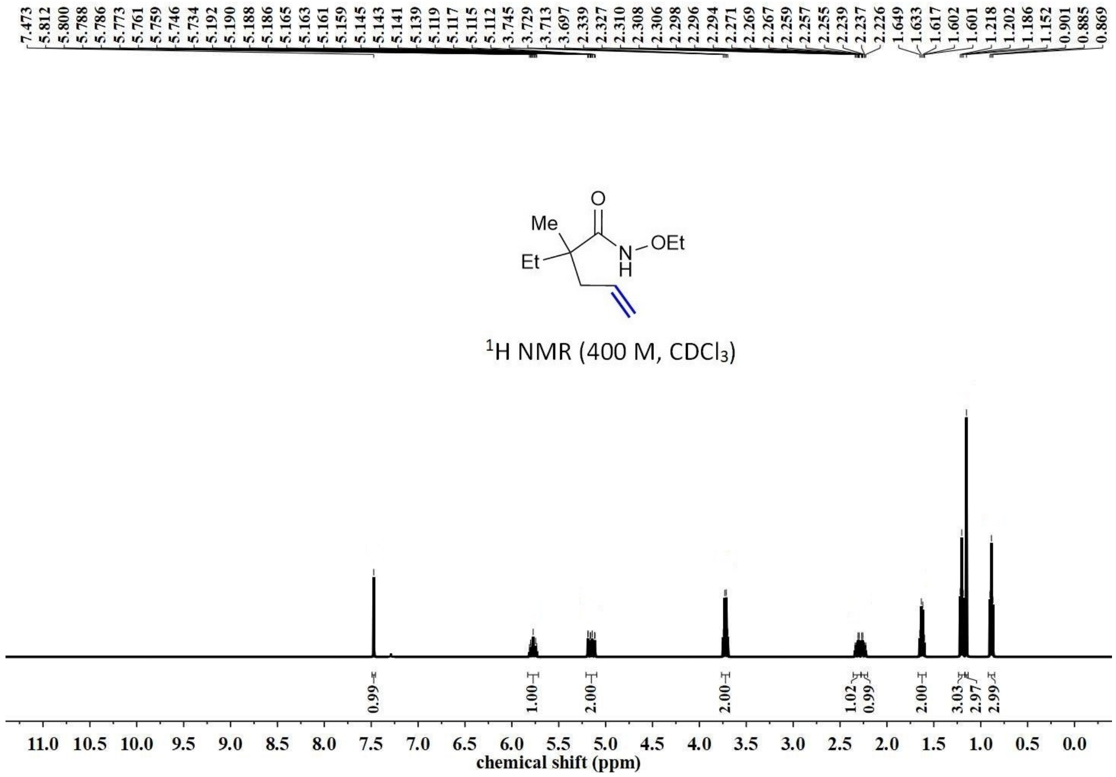

^{1}H NMR (400 M, $CDCl_3$)

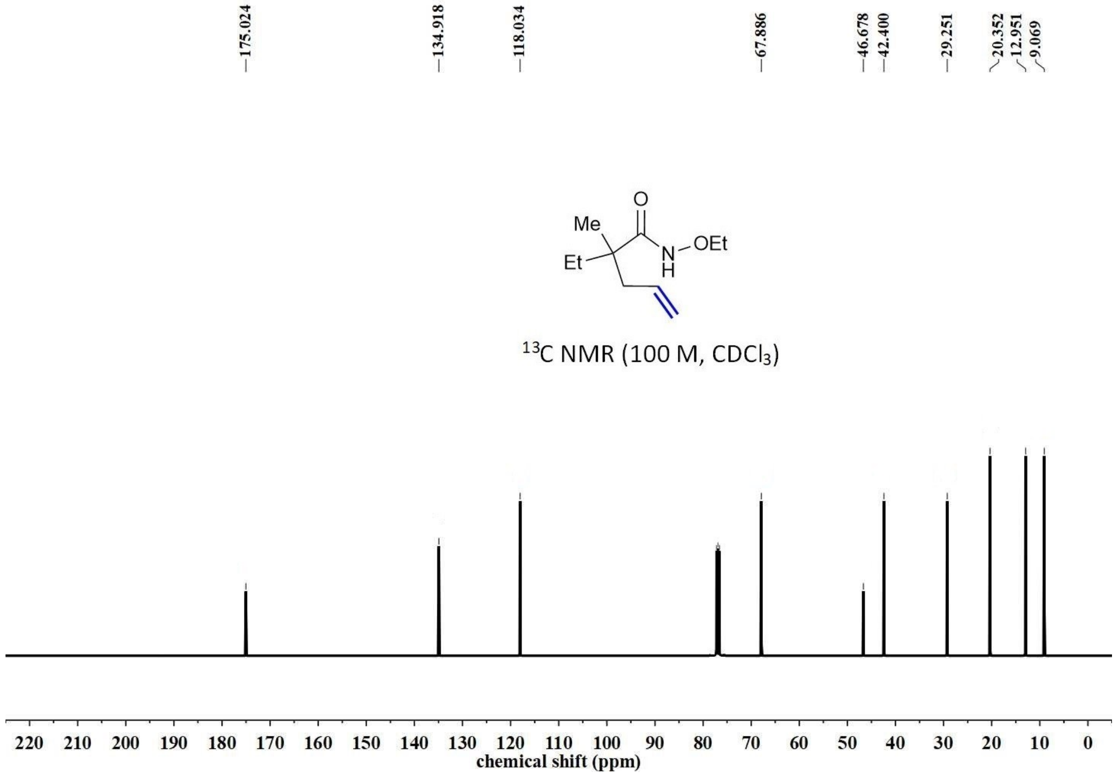

^{13}C NMR (100 M, $CDCl_3$)

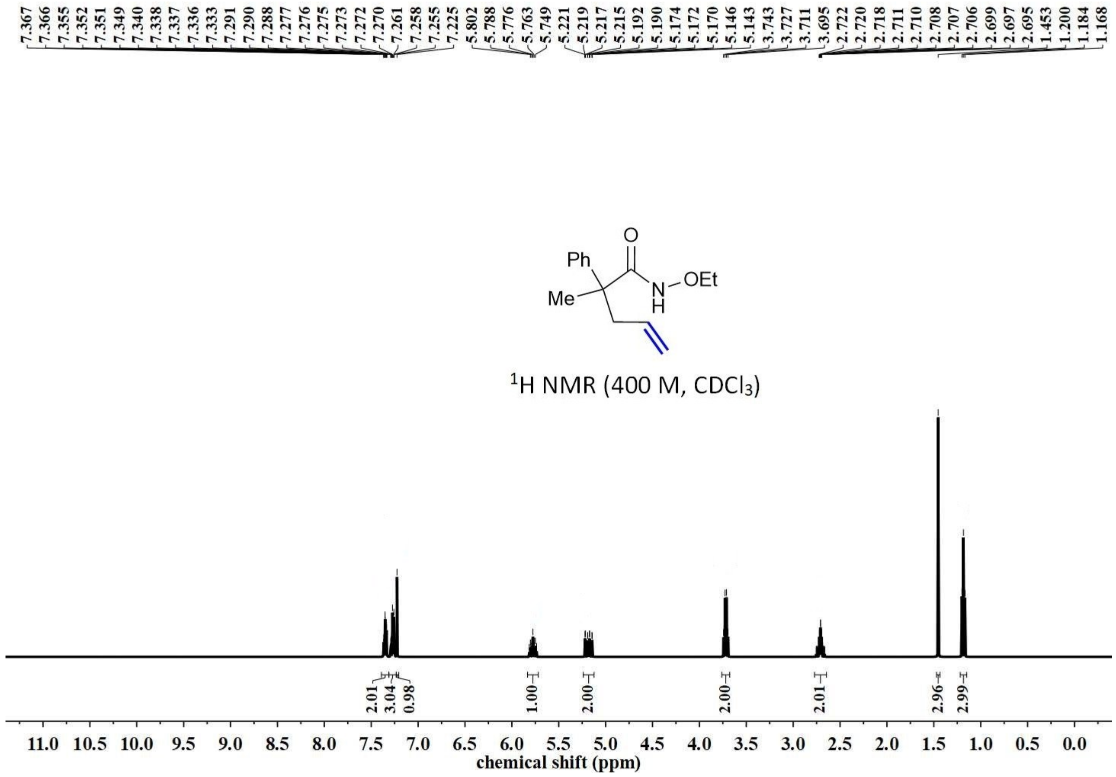

^1H NMR (400 M, $CDCl_3$)

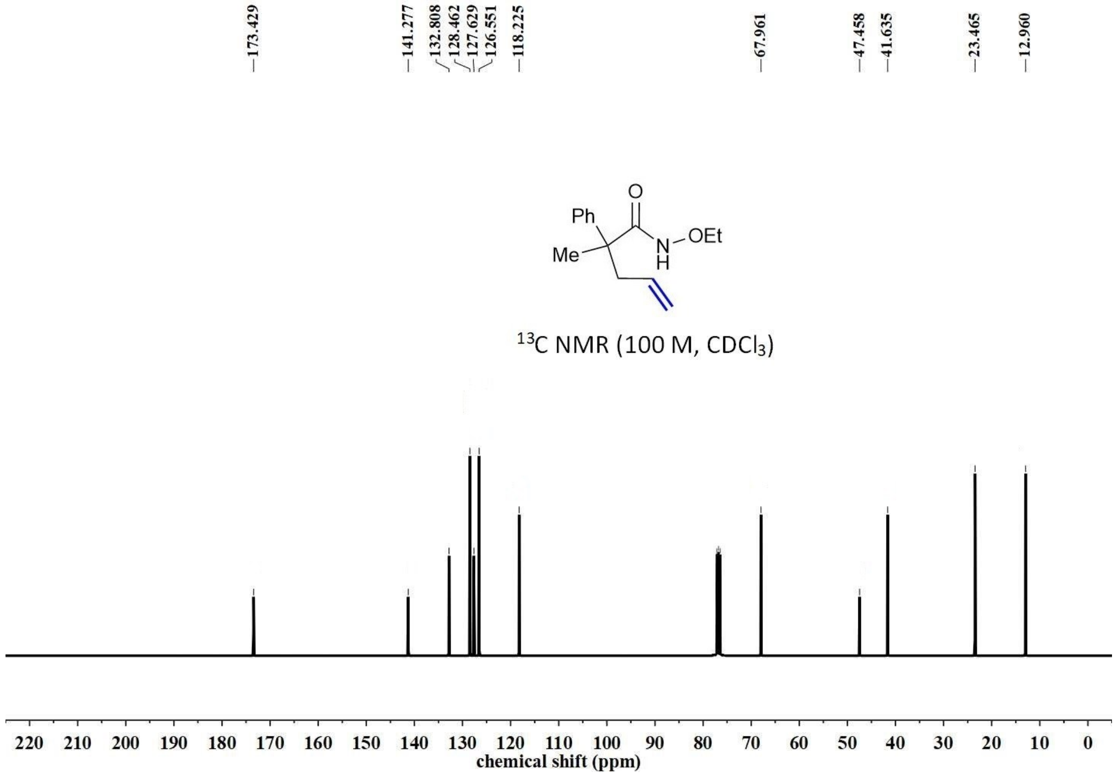

^{13}C NMR (100 M, $CDCl_3$)

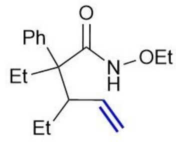

^{1}H NMR (400 M, $CDCl_3$)

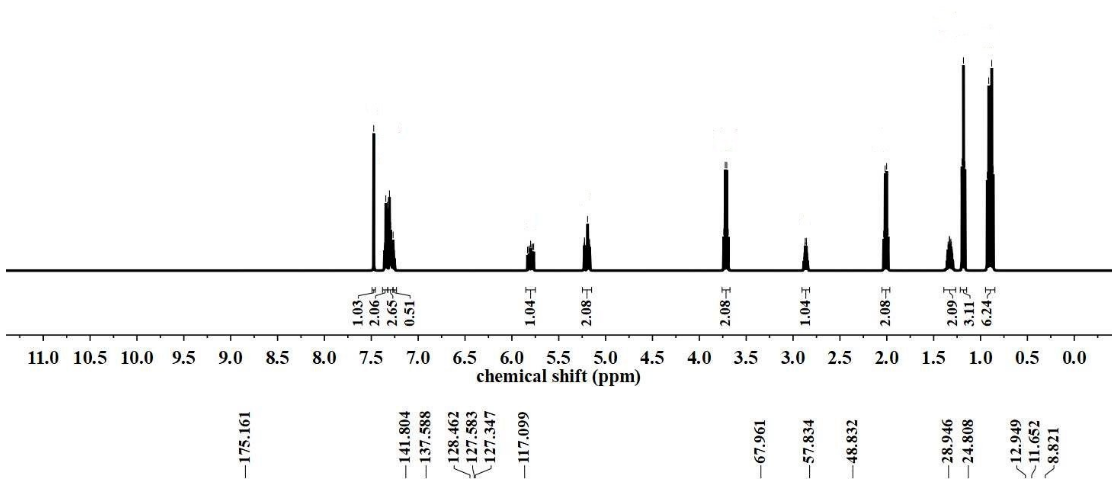

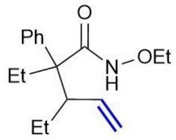

^{13}C NMR (100 M, $CDCl_3$)

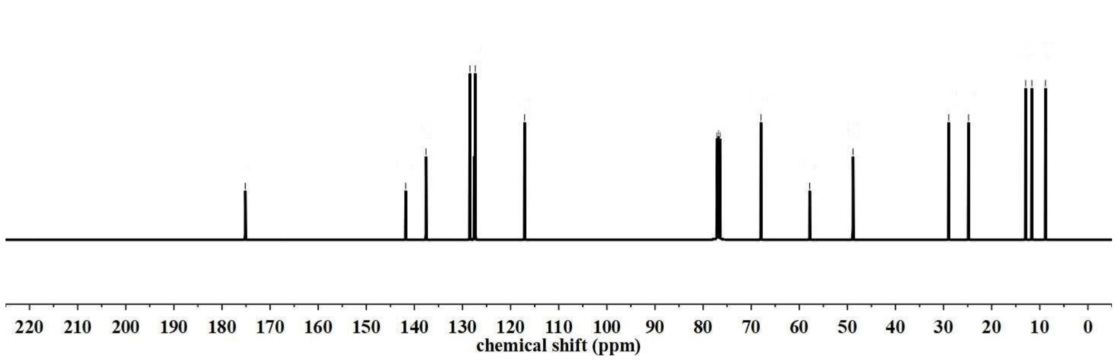

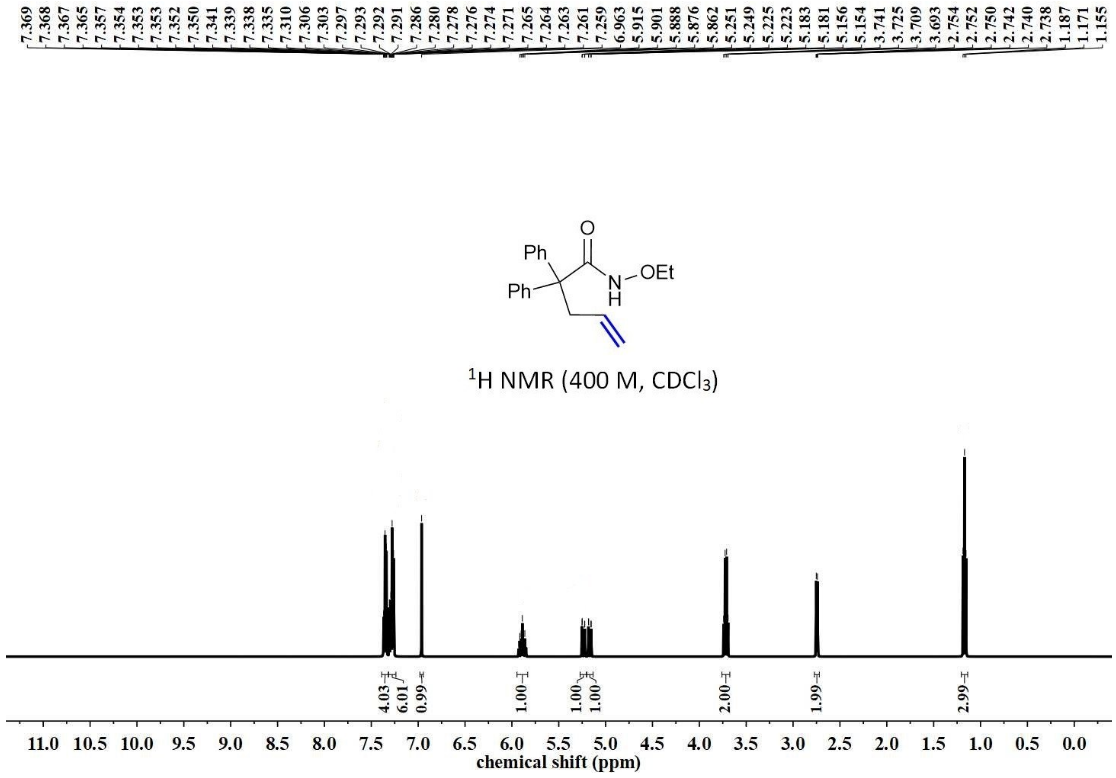
7.369 7.368 7.367 7.365 7.357 7.354 7.353 7.353 7.352 7.350 7.341 7.339 7.338 7.335 7.310 7.306 7.303 7.297 7.293 7.292 7.291 7.286 7.280 7.278 7.276 7.274 7.271 7.265 7.264 7.263 7.261 7.259 6.963 5.915 5.901 5.888 5.876 5.862 5.251 5.249 5.225 5.223 5.183 5.181 5.156 5.154 3.741 3.725 3.709 3.693 2.754 2.752 2.750 2.742 2.740 2.738 1.187 1.171 1.155
Ph
Ph
O
N
H
OEt
^{1}H NMR (400 M, $CDCl_3$)
4.03 6.01 0.99 1.00 1.00 1.00 2.00 1.99 2.99
11.0 10.5 10.0 9.5 9.0 8.5 8.0 7.5 7.0 6.5 6.0 5.5 5.0 4.5 4.0 3.5 3.0 2.5 2.0 1.5 1.0 0.5 0.0
chemical shift (ppm)

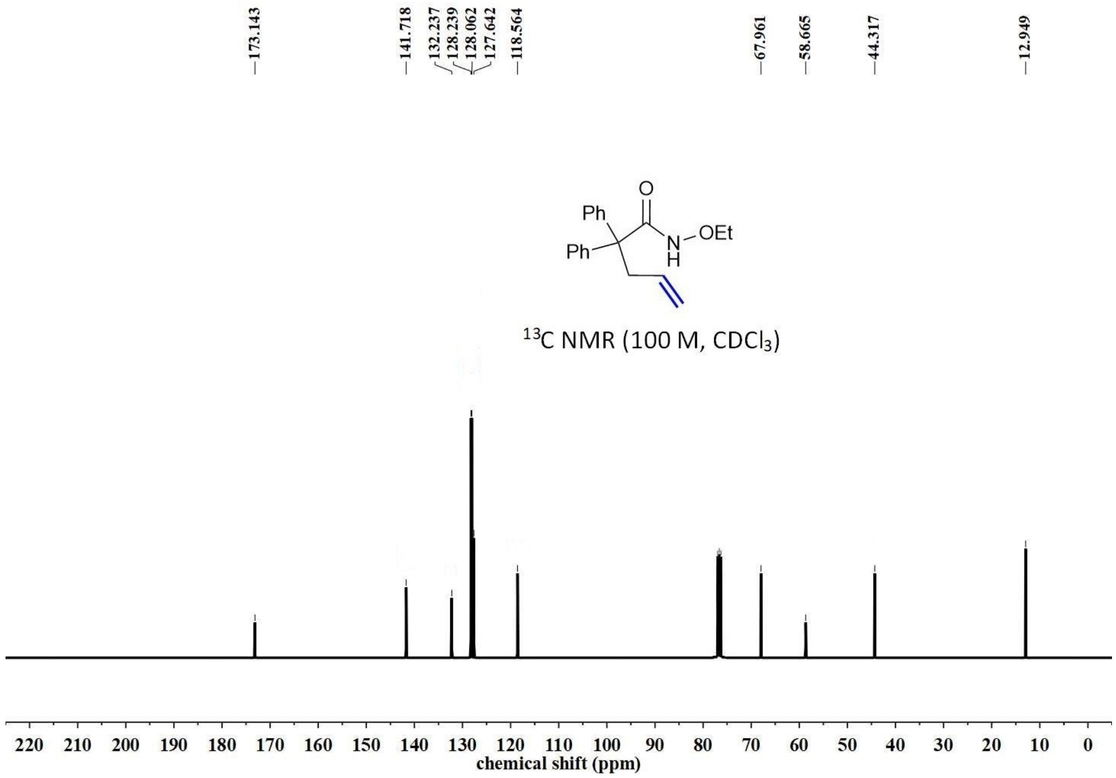
173.143 141.718 132.237 128.239 128.062 127.642 118.564 67.961 58.665 44.317 12.949
Ph
Ph
O
N
H
OEt
^{13}C NMR (100 M, $CDCl_3$)
220 210 200 190 180 170 160 150 140 130 120 110 100 90 80 70 60 50 40 30 20 10 0
chemical shift (ppm)

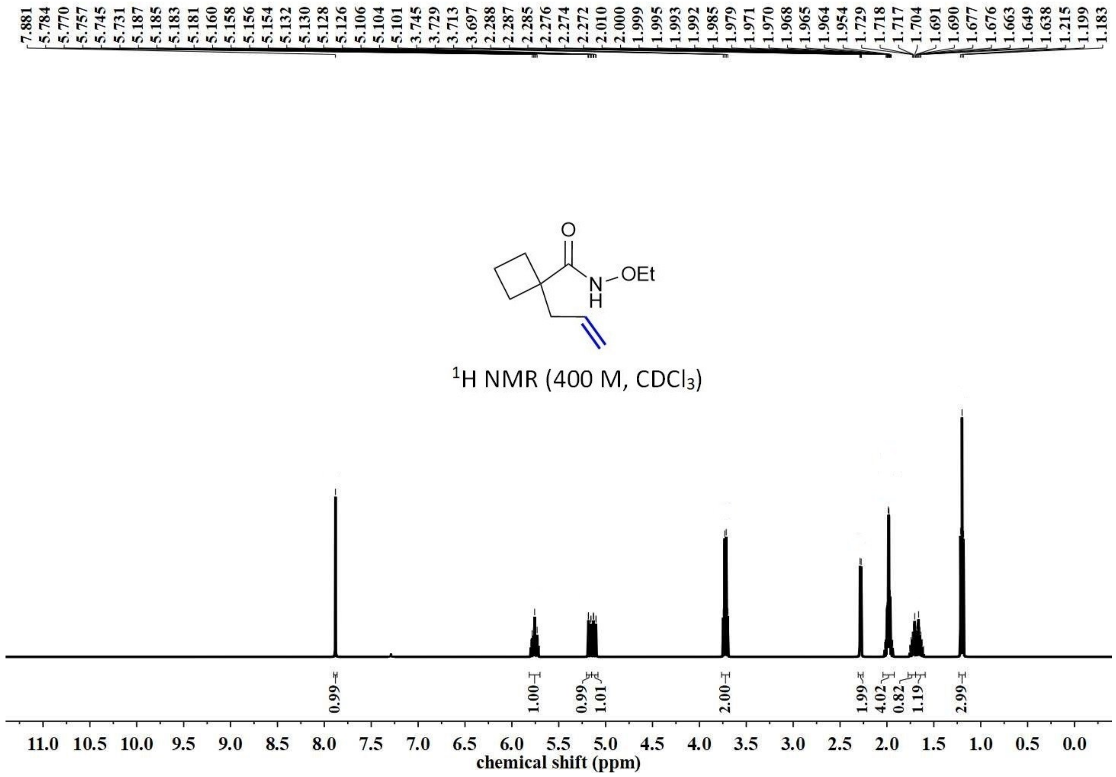
7.881 5.784 5.770 5.757 5.745 5.731 5.187 5.185 5.183 5.181 5.160 5.158 5.156 5.154 5.132 5.130 5.128 5.126 5.106 5.104 5.101 3.745 3.729 3.713 3.697 2.288 2.287 2.285 2.276 2.274 2.272 2.010 2.000 1.999 1.995 1.993 1.992 1.985 1.979 1.971 1.970 1.968 1.965 1.964 1.954 1.729 1.718 1.717 1.704 1.691 1.690 1.677 1.676 1.663 1.649 1.638 1.215 1.199 1.183
O
N
H
OEt
^{1}H NMR (400 M, CDCl$_3$)
0.99
1.00
0.99
1.01
2.00
1.99
4.02
0.82
1.19
2.99
11.0 10.5 10.0 9.5 9.0 8.5 8.0 7.5 7.0 6.5 6.0 5.5 5.0 4.5 4.0 3.5 3.0 2.5 2.0 1.5 1.0 0.5 0.0
chemical shift (ppm)

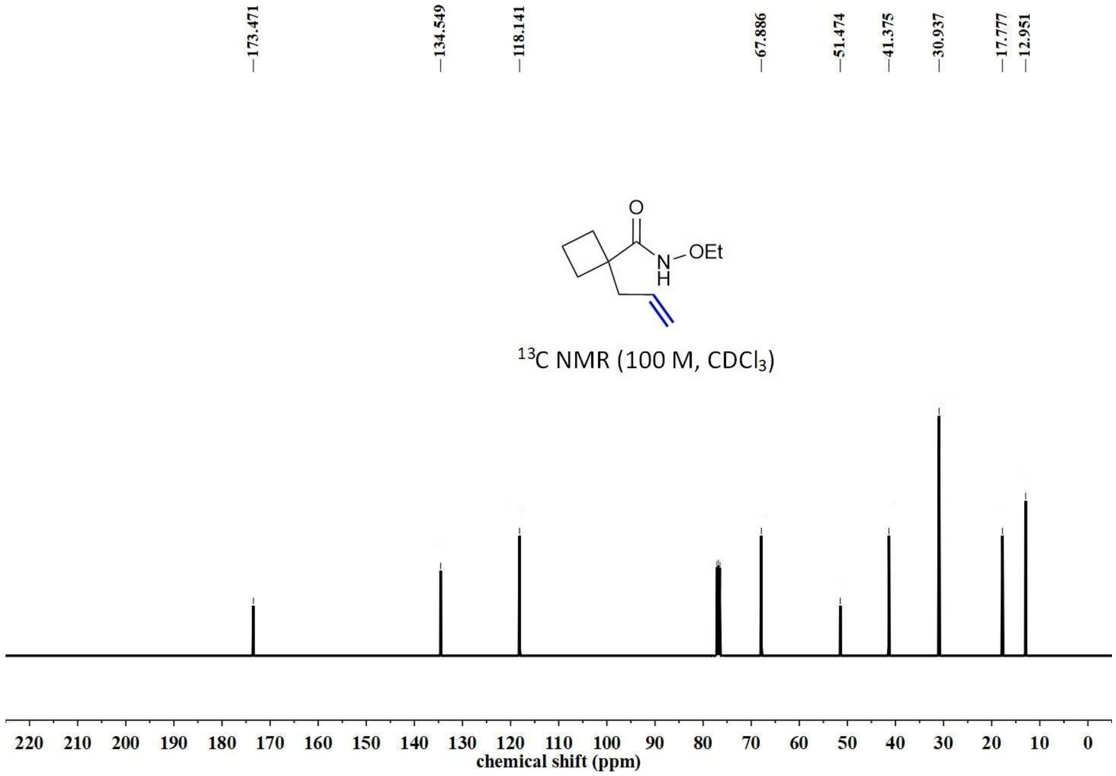
173.471
134.549
118.141
67.886
51.474
41.375
30.937
17.777
12.951
O
N
H
OEt
^{13}C NMR (100 M, CDCl$_3$)
220 210 200 190 180 170 160 150 140 130 120 110 100 90 80 70 60 50 40 30 20 10 0
chemical shift (ppm)

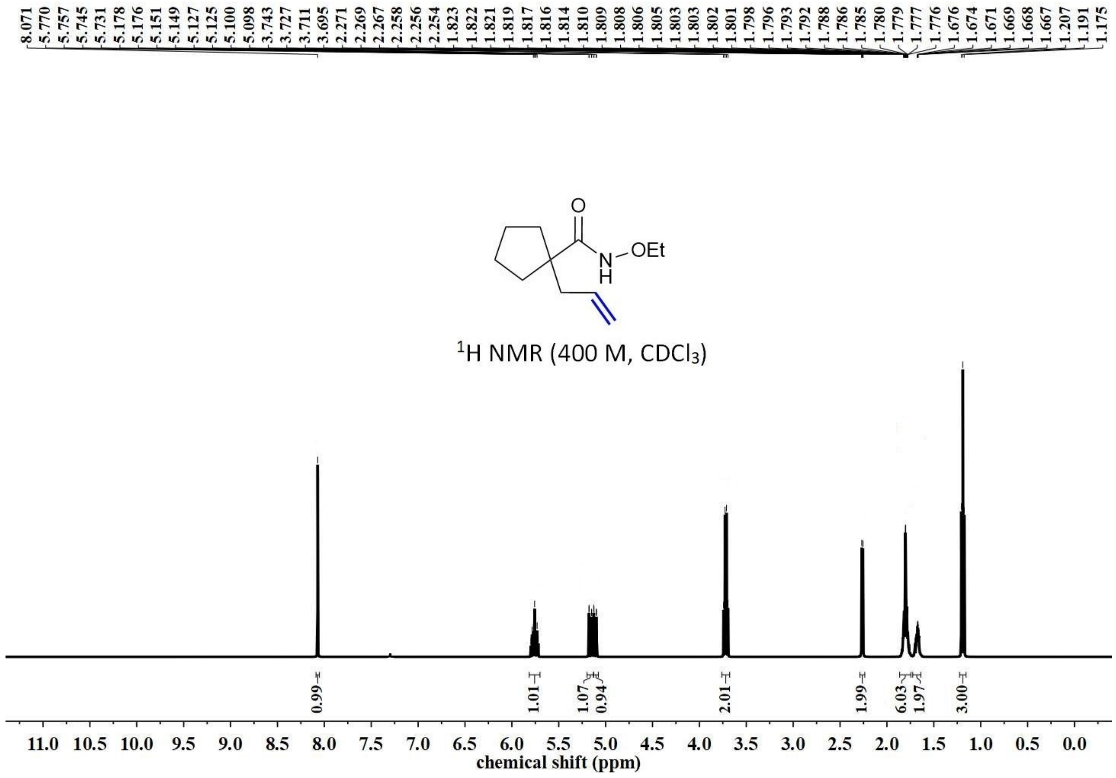
O
N
H
OEt
1H NMR (400 M, CDCl3)
chemical shift (ppm)

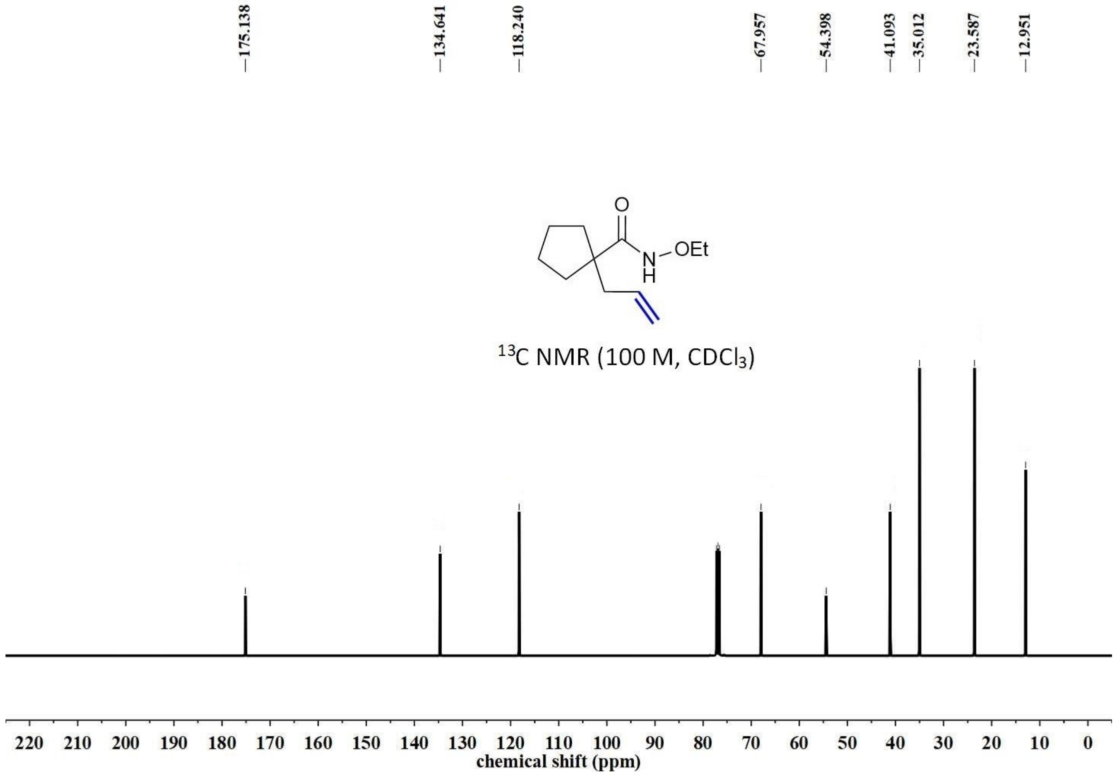
175.138
134.641
118.240
67.957
54.398
41.093
35.012
23.587
12.951
O
N
H
OEt
13C NMR (100 M, CDCl3)
chemical shift (ppm)

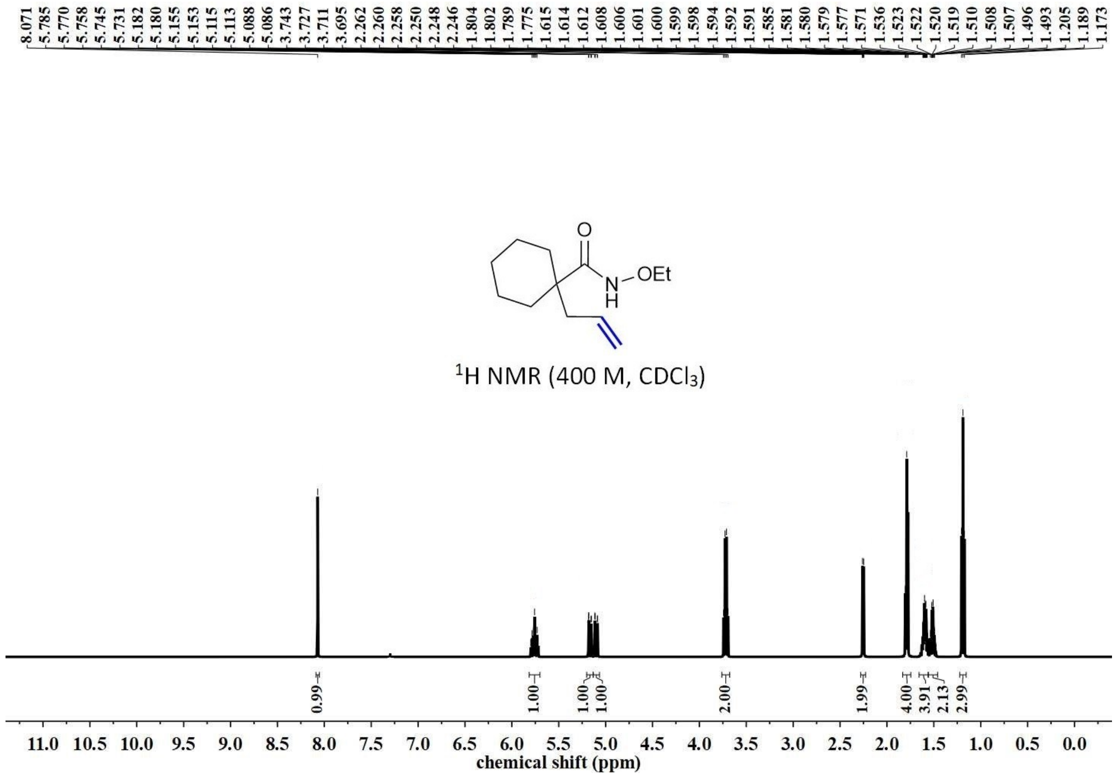

^{1}H NMR (400 M, $CDCl_3$)

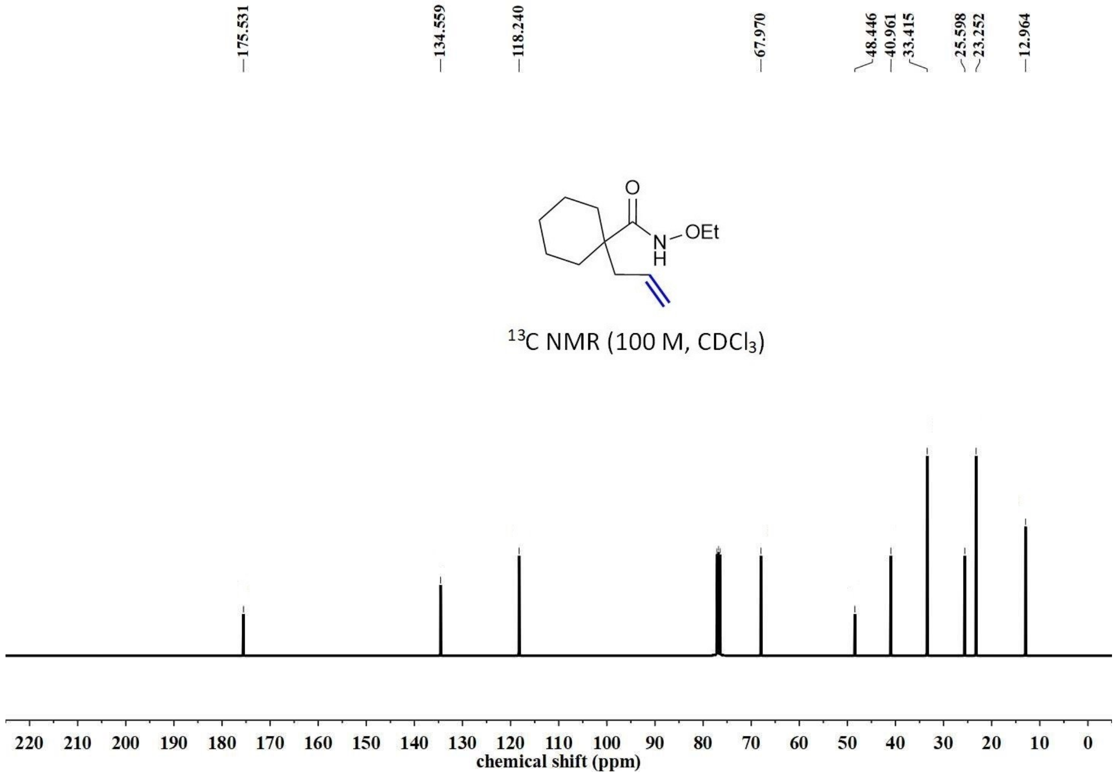

^{13}C NMR (100 M, $CDCl_3$)

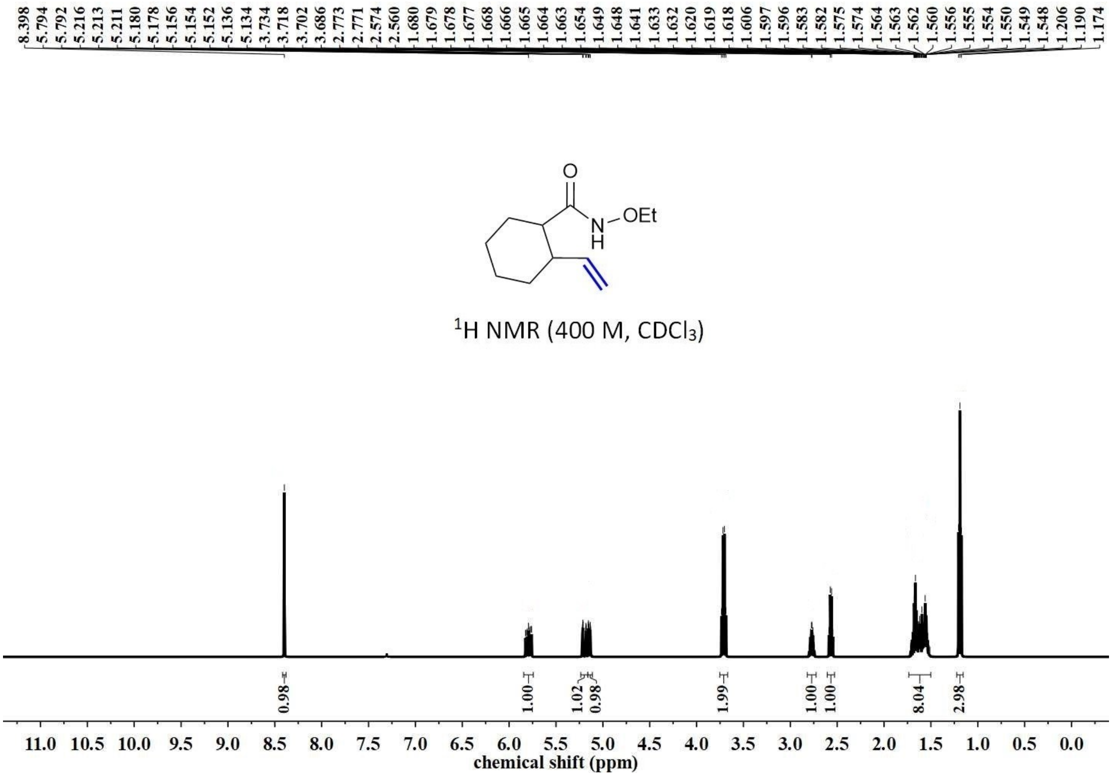
8.398
5.794
5.792
5.216
5.213
5.211
5.180
5.178
5.156
5.154
5.152
5.136
5.134
3.734
3.718
3.702
3.686
2.773
2.771
2.574
2.560
1.680
1.679
1.678
1.677
1.668
1.666
1.665
1.664
1.663
1.654
1.649
1.648
1.641
1.633
1.632
1.620
1.619
1.618
1.606
1.597
1.596
1.583
1.582
1.575
1.574
1.564
1.563
1.562
1.560
1.556
1.555
1.554
1.550
1.549
1.548
1.206
1.190
1.174
O
N
H
OEt
^{1}H NMR (400 M, CDCl$_3$)
0.98
1.00
1.02
0.98
1.99
1.00
1.00
8.04
2.98
11.0 10.5 10.0 9.5 9.0 8.5 8.0 7.5 7.0 6.5 6.0 5.5 5.0 4.5 4.0 3.5 3.0 2.5 2.0 1.5 1.0 0.5 0.0
chemical shift (ppm)

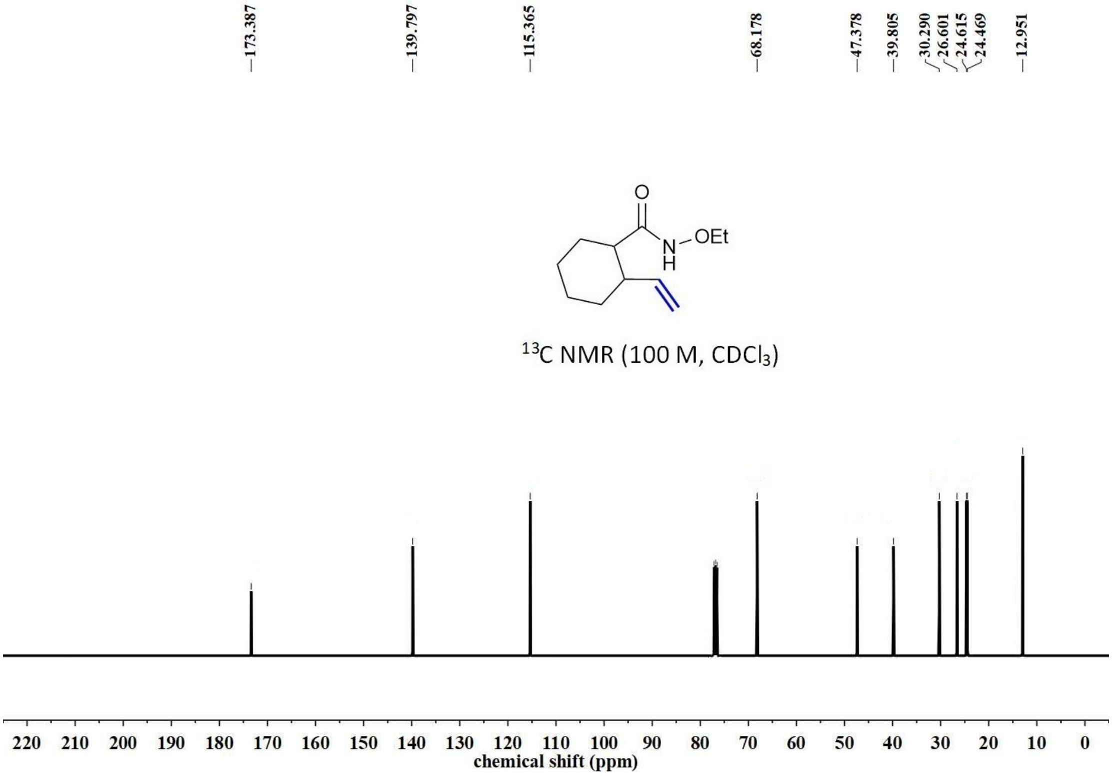
173.387
139.797
115.365
68.178
47.378
39.805
30.290
26.601
24.615
24.469
12.951
O
N
H
OEt
^{13}C NMR (100 M, CDCl$_3$)
220 210 200 190 180 170 160 150 140 130 120 110 100 90 80 70 60 50 40 30 20 10 0
chemical shift (ppm)

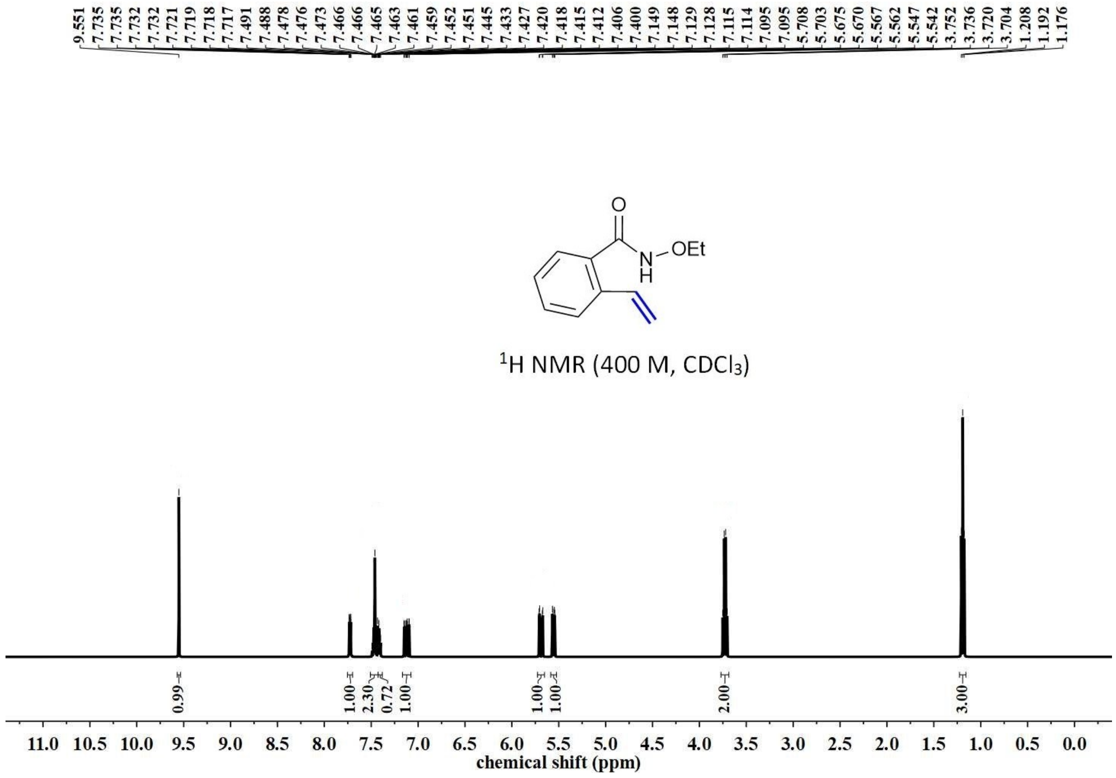
9.551 7.735 7.735 7.732 7.732 7.721 7.719 7.718 7.717 7.491 7.488 7.478 7.476 7.473 7.466 7.466 7.465 7.463 7.461 7.459 7.452 7.451 7.445 7.433 7.427 7.420 7.418 7.415 7.412 7.406 7.400 7.149 7.148 7.129 7.128 7.115 7.114 7.095 7.095 5.708 5.703 5.675 5.670 5.567 5.562 5.547 5.542 3.752 3.736 3.720 3.704 1.208 1.192 1.176
O
N
H
OEt
^{1}H NMR (400 M, CDCl$_3$)
0.99
1.00
2.30
0.72
1.00
1.00
1.00
2.00
3.00
11.0 10.5 10.0 9.5 9.0 8.5 8.0 7.5 7.0 6.5 6.0 5.5 5.0 4.5 4.0 3.5 3.0 2.5 2.0 1.5 1.0 0.5 0.0
chemical shift (ppm)

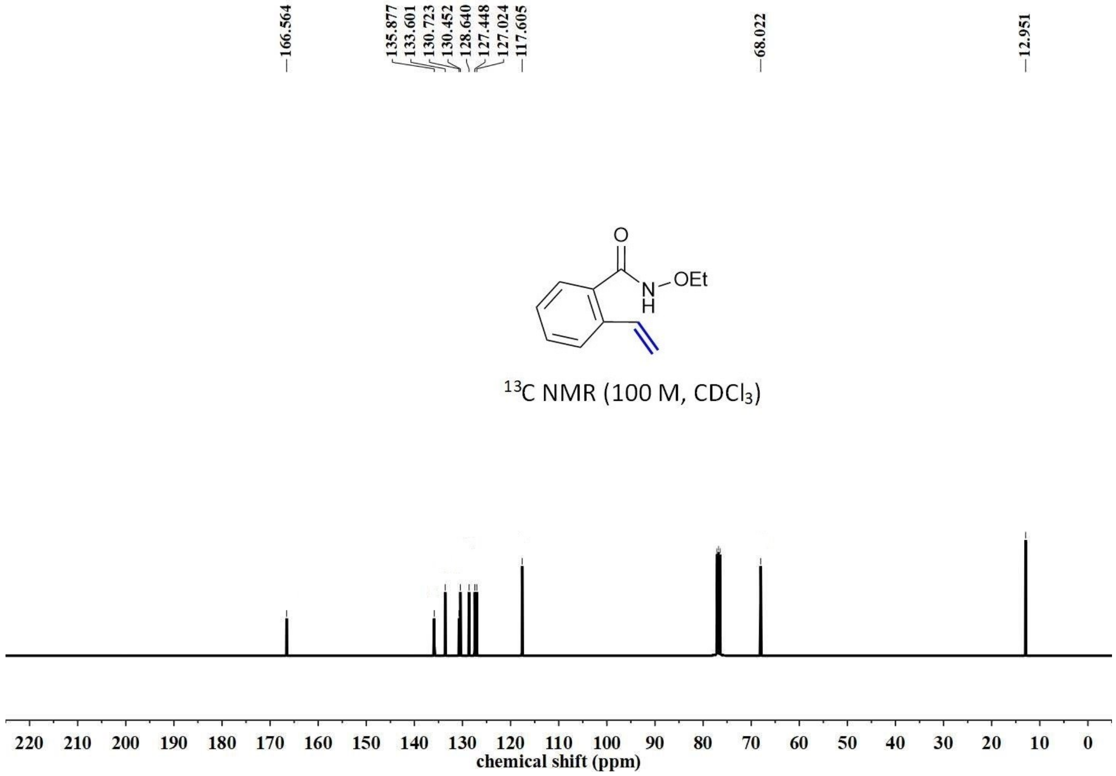
166.564
135.877 133.601 130.723 130.452 128.640 127.448 127.024 117.605
68.022
12.951
O
N
H
OEt
^{13}C NMR (100 M, CDCl$_3$)
220 210 200 190 180 170 160 150 140 130 120 110 100 90 80 70 60 50 40 30 20 10 0
chemical shift (ppm)

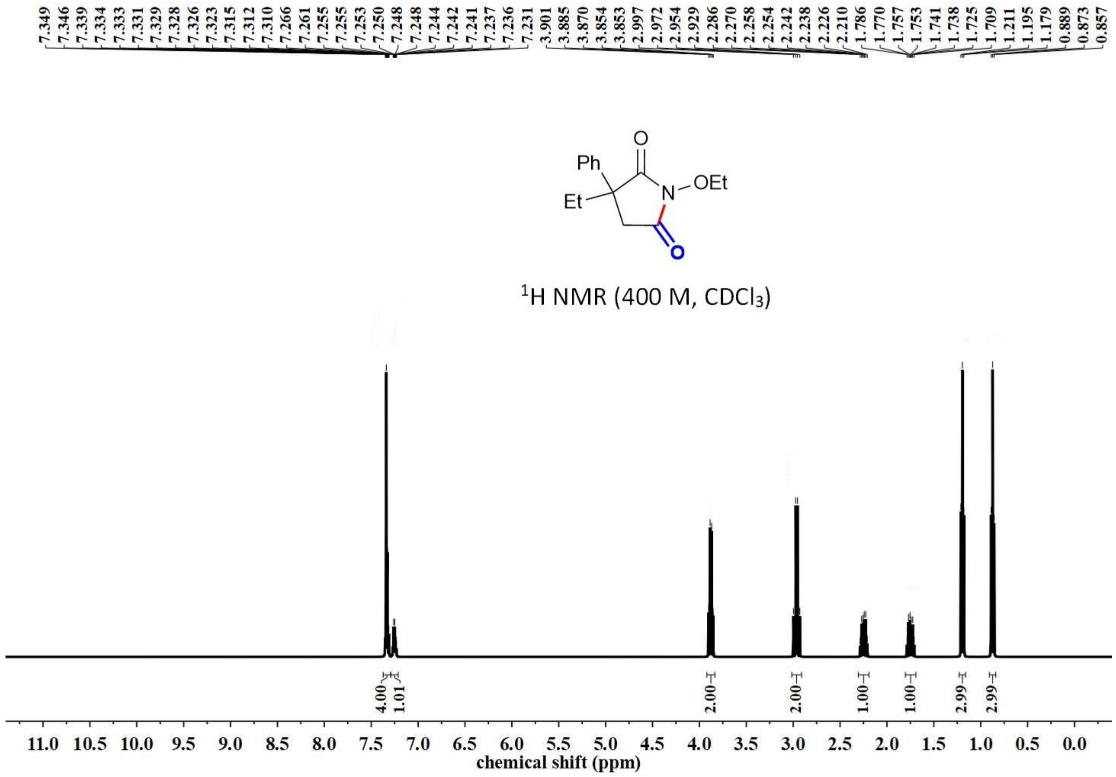
7.349 7.346 7.339 7.334 7.333 7.331 7.329 7.328 7.326 7.323 7.315 7.312 7.310 7.266 7.261 7.255 7.255 7.253 7.250 7.248 7.248 7.244 7.242 7.241 7.237 7.236 7.231 3.901 3.885 3.870 3.854 3.853 2.997 2.972 2.954 2.929 2.286 2.270 2.258 2.254 2.242 2.238 2.226 2.210 1.786 1.770 1.757 1.753 1.741 1.738 1.725 1.709 1.211 1.195 1.179 0.889 0.873 0.857
Ph
O
N
OEt
Et
O
^{1}H NMR (400 M, CDCl$_3$)
4.00 1.01 2.00 2.00 1.00 1.00 2.99 2.99
11.0 10.5 10.0 9.5 9.0 8.5 8.0 7.5 7.0 6.5 6.0 5.5 5.0 4.5 4.0 3.5 3.0 2.5 2.0 1.5 1.0 0.5 0.0
chemical shift (ppm)

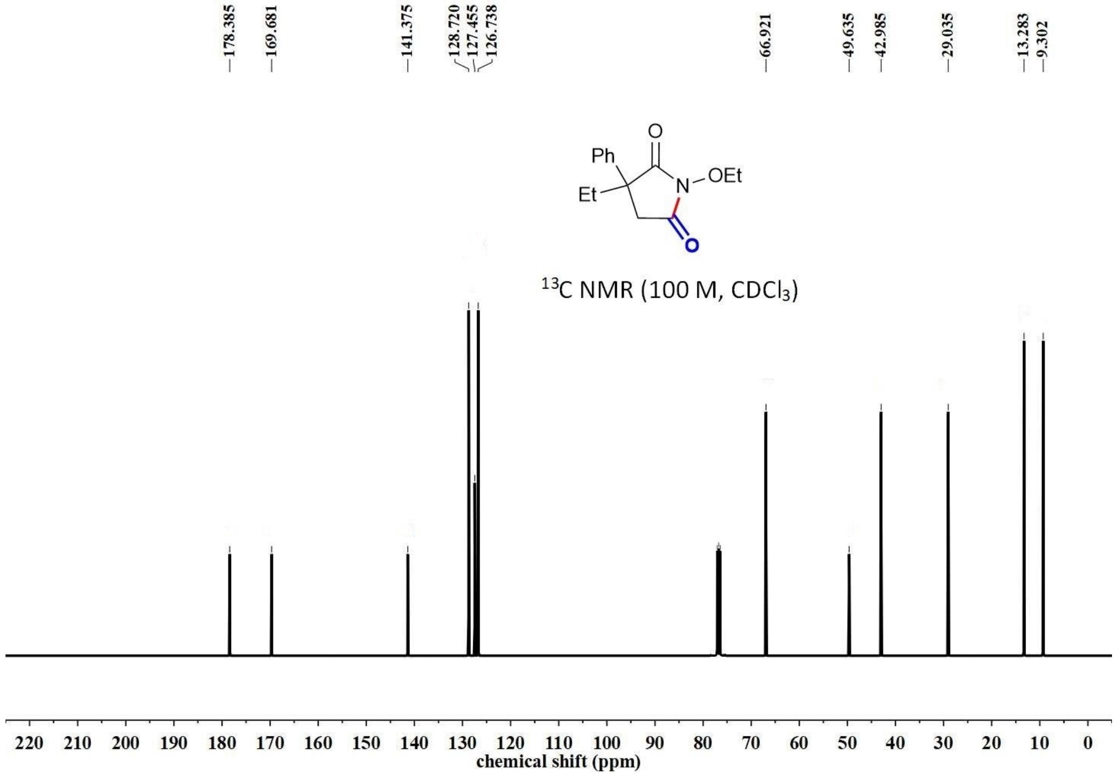
178.385 169.681 141.375 128.720 127.455 126.738 66.921 49.635 42.985 29.035 13.283 9.302
Ph
O
N
OEt
Et
O
^{13}C NMR (100 M, CDCl$_3$)
220 210 200 190 180 170 160 150 140 130 120 110 100 90 80 70 60 50 40 30 20 10 0
chemical shift (ppm)

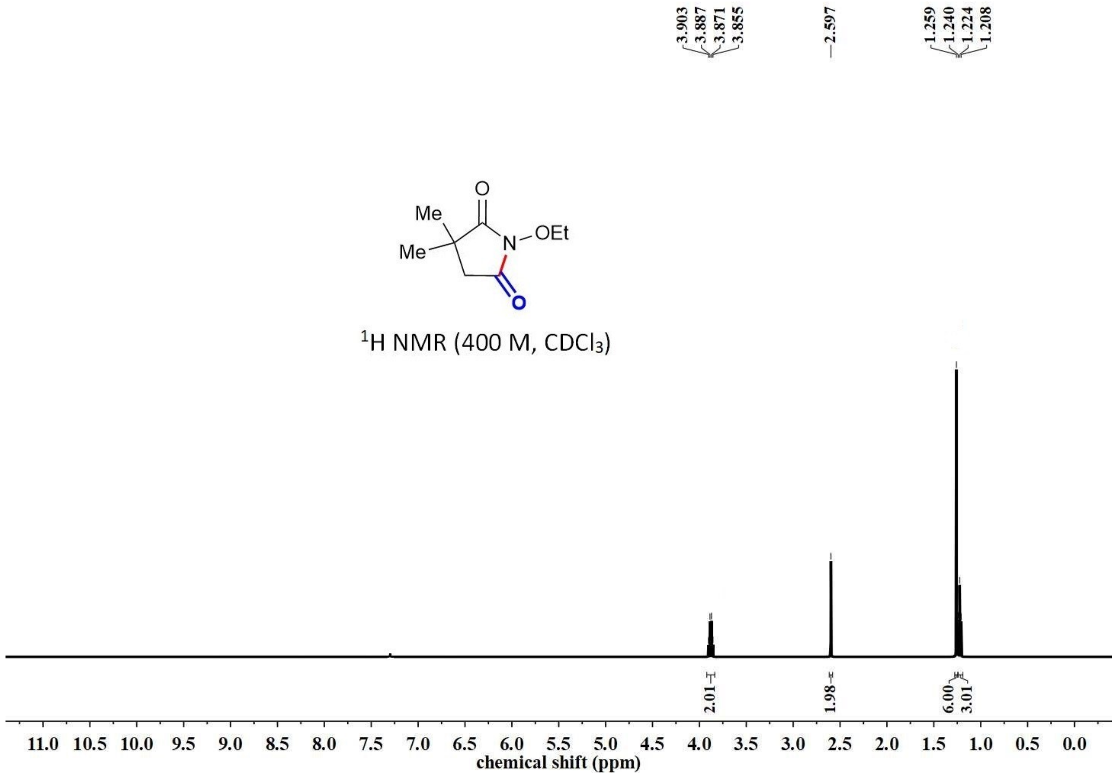

3.903
3.887
3.871
3.855
2.597
1.259
1.240
1.224
1.208
Me
Me
O
N
OEt
O
^{1}H NMR (400 M, CDCl$_3$)
2.01
1.98
6.00
3.01
11.0 10.5 10.0 9.5 9.0 8.5 8.0 7.5 7.0 6.5 6.0 5.5 5.0 4.5 4.0 3.5 3.0 2.5 2.0 1.5 1.0 0.5 0.0
chemical shift (ppm)

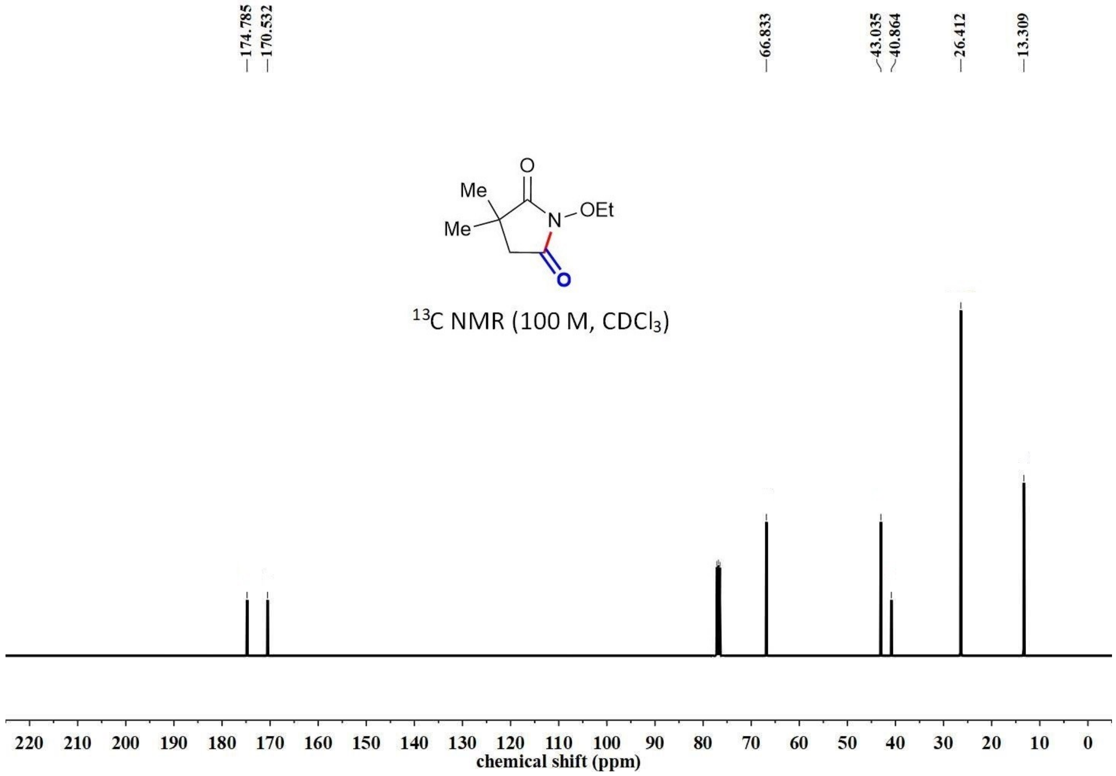

174.785
170.532
66.833
43.035
40.864
26.412
13.309
Me
Me
O
N
OEt
O
^{13}C NMR (100 M, CDCl$_3$)
220 210 200 190 180 170 160 150 140 130 120 110 100 90 80 70 60 50 40 30 20 10 0
chemical shift (ppm)

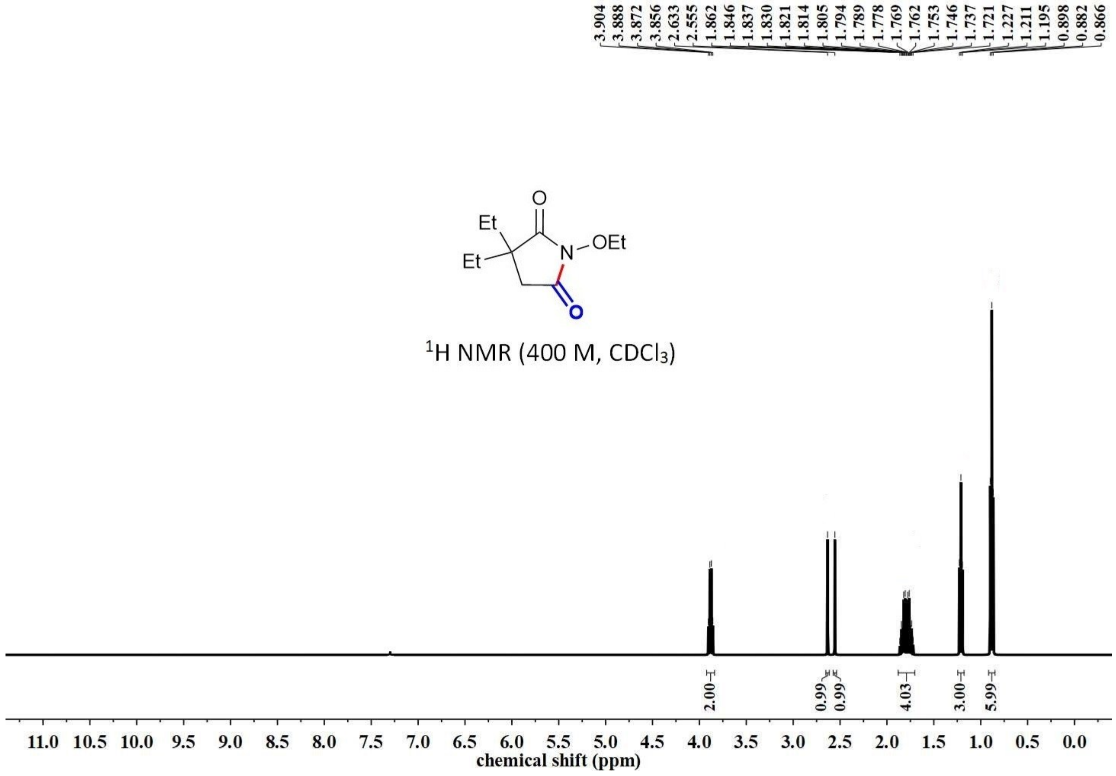
3.904
3.888
3.872
3.856
2.633
2.555
1.862
1.846
1.837
1.830
1.821
1.814
1.805
1.794
1.789
1.778
1.769
1.762
1.753
1.746
1.737
1.721
1.227
1.211
1.195
0.898
0.882
0.866
Et
Et
N
OEt
O
O
^{1}H NMR (400 M, $CDCl_3$)
2.00
0.99
0.99
4.03
3.00
5.99
11.0 10.5 10.0 9.5 9.0 8.5 8.0 7.5 7.0 6.5 6.0 5.5 5.0 4.5 4.0 3.5 3.0 2.5 2.0 1.5 1.0 0.5 0.0
chemical shift (ppm)

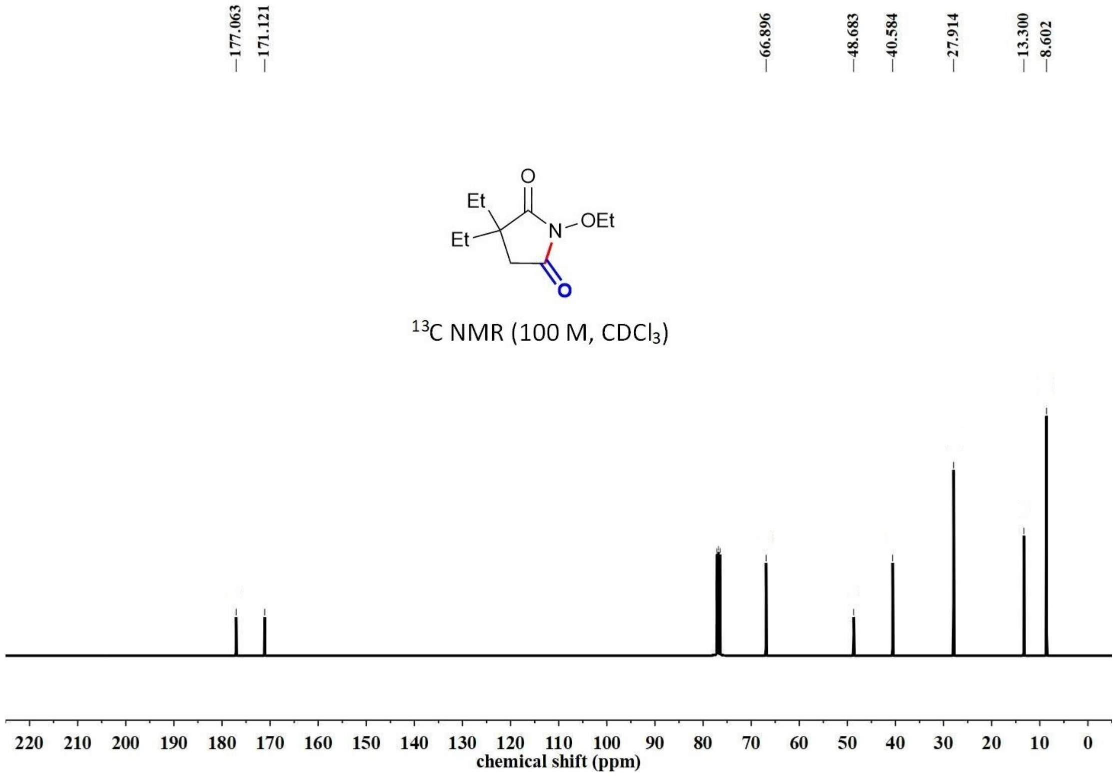
177.063
171.121
66.896
48.683
40.584
27.914
13.300
8.602
Et
Et
N
OEt
O
O
^{13}C NMR (100 M, $CDCl_3$)
220 210 200 190 180 170 160 150 140 130 120 110 100 90 80 70 60 50 40 30 20 10 0
chemical shift (ppm)

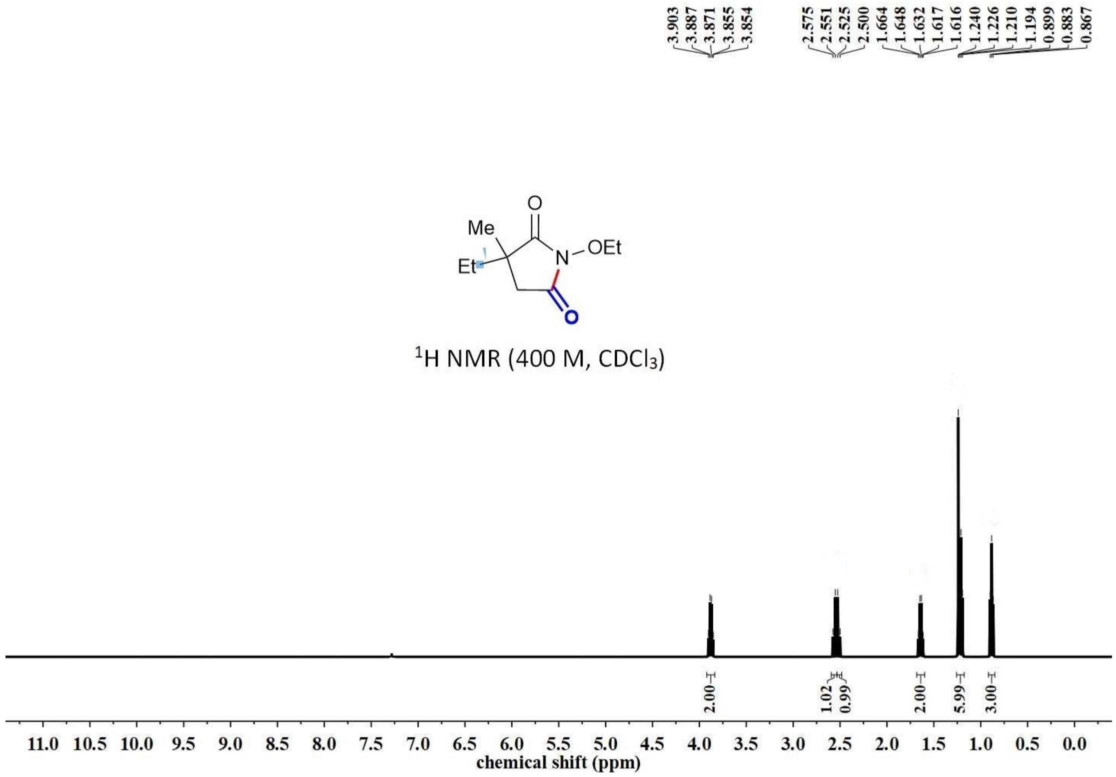

^{1}H NMR (400 M, $CDCl_3$)

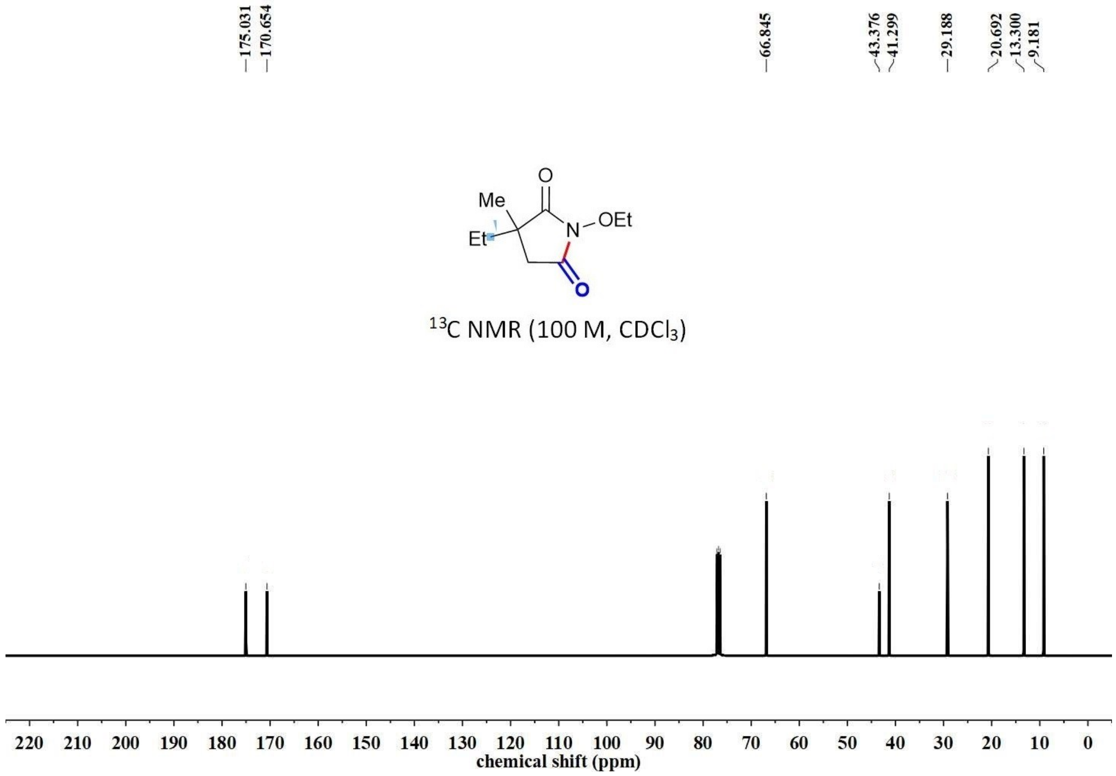

^{13}C NMR (100 M, $CDCl_3$)

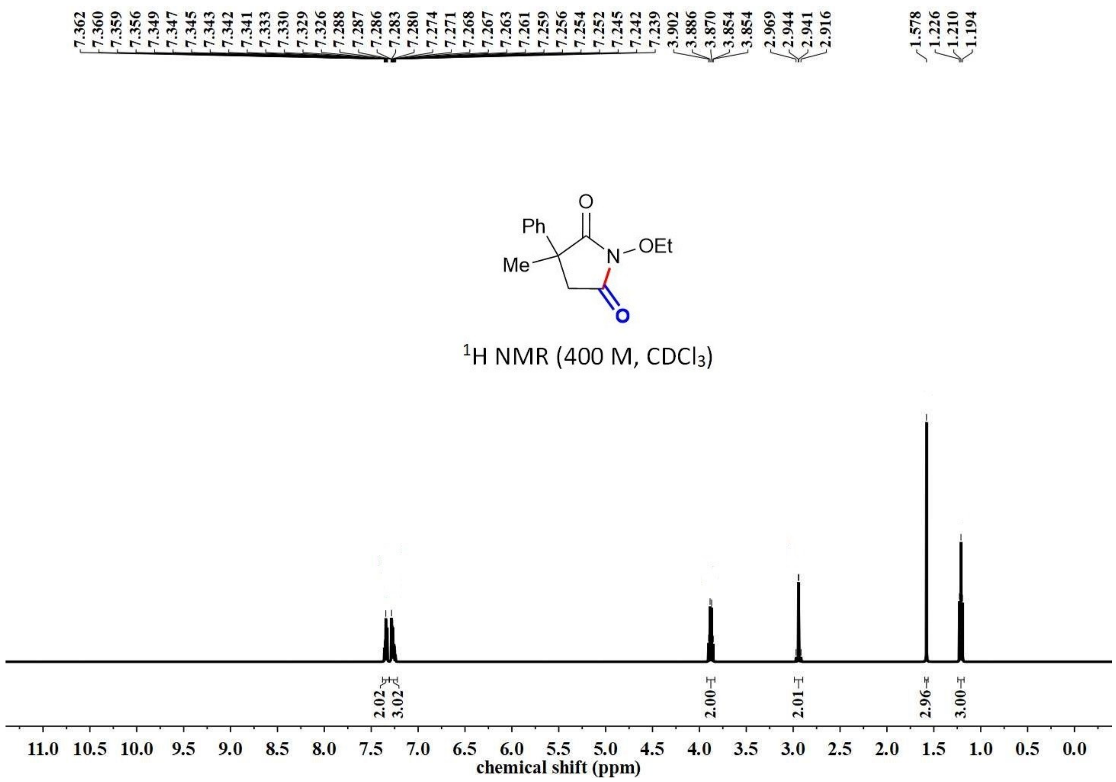

^1H NMR (400 M, $CDCl_3$)

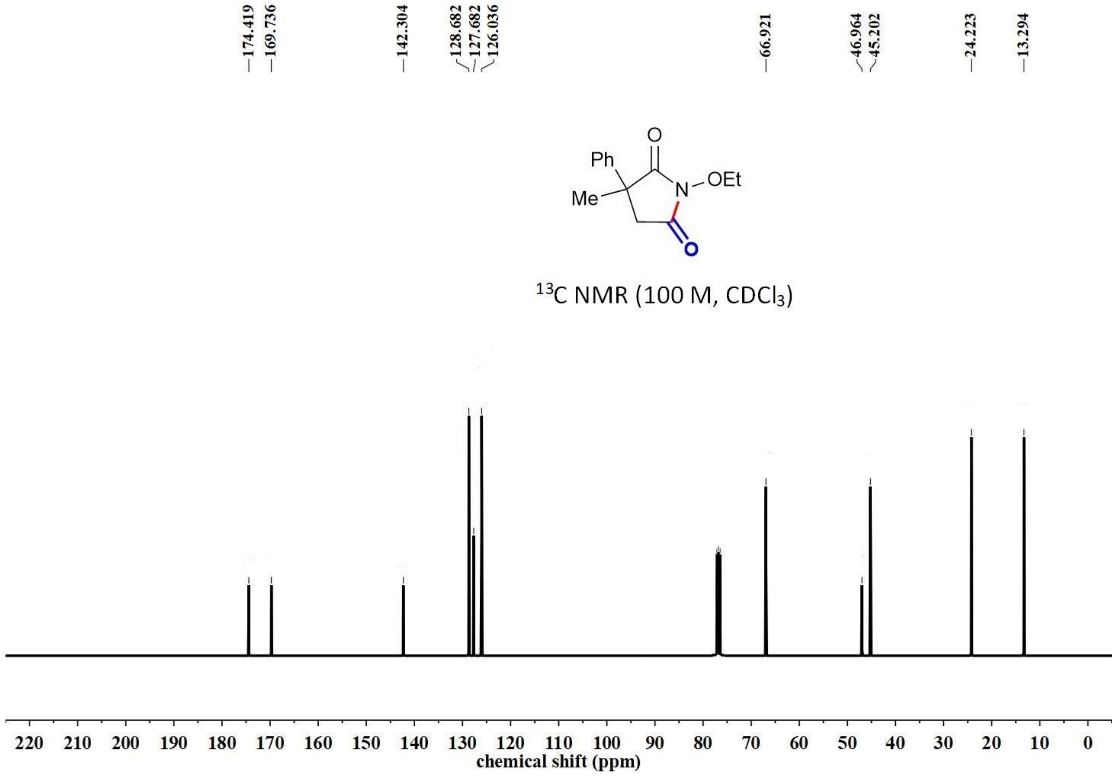

^{13}C NMR (100 M, $CDCl_3$)

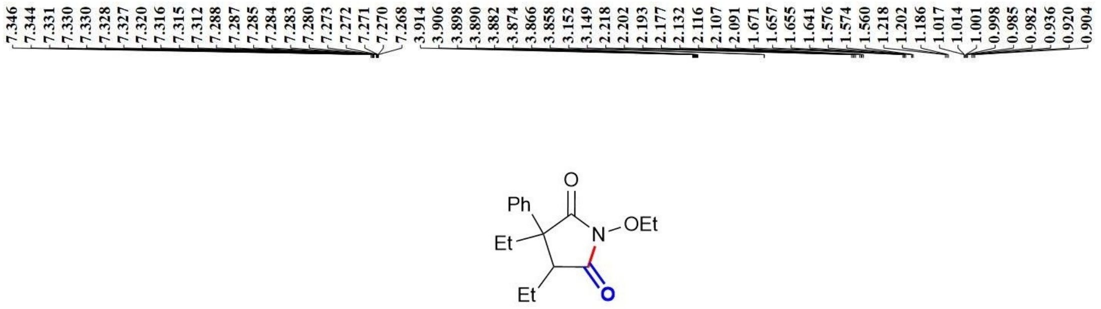

^{1}H NMR (400 M, $CDCl_3$)

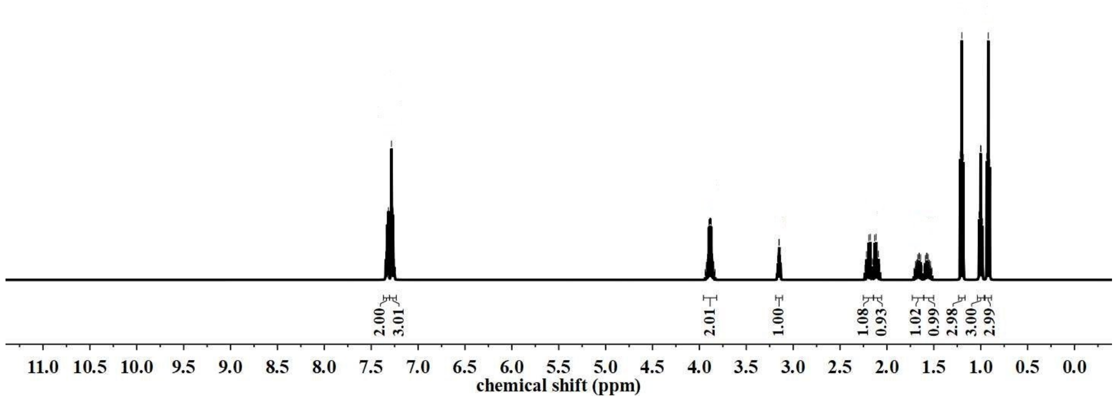

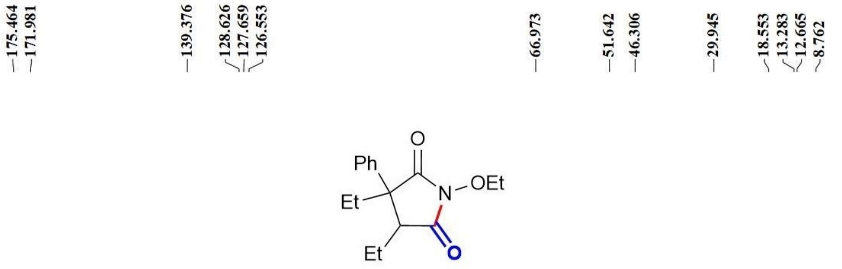

^{13}C NMR (100 M, $CDCl_3$)

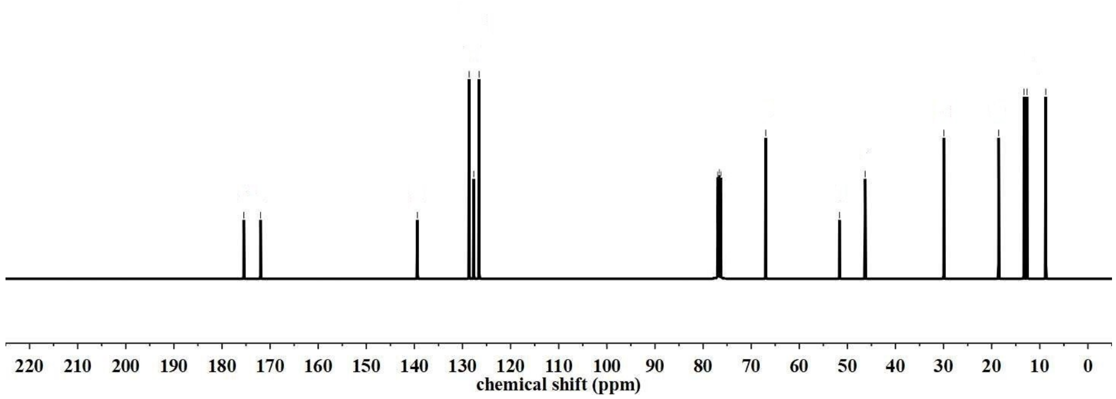

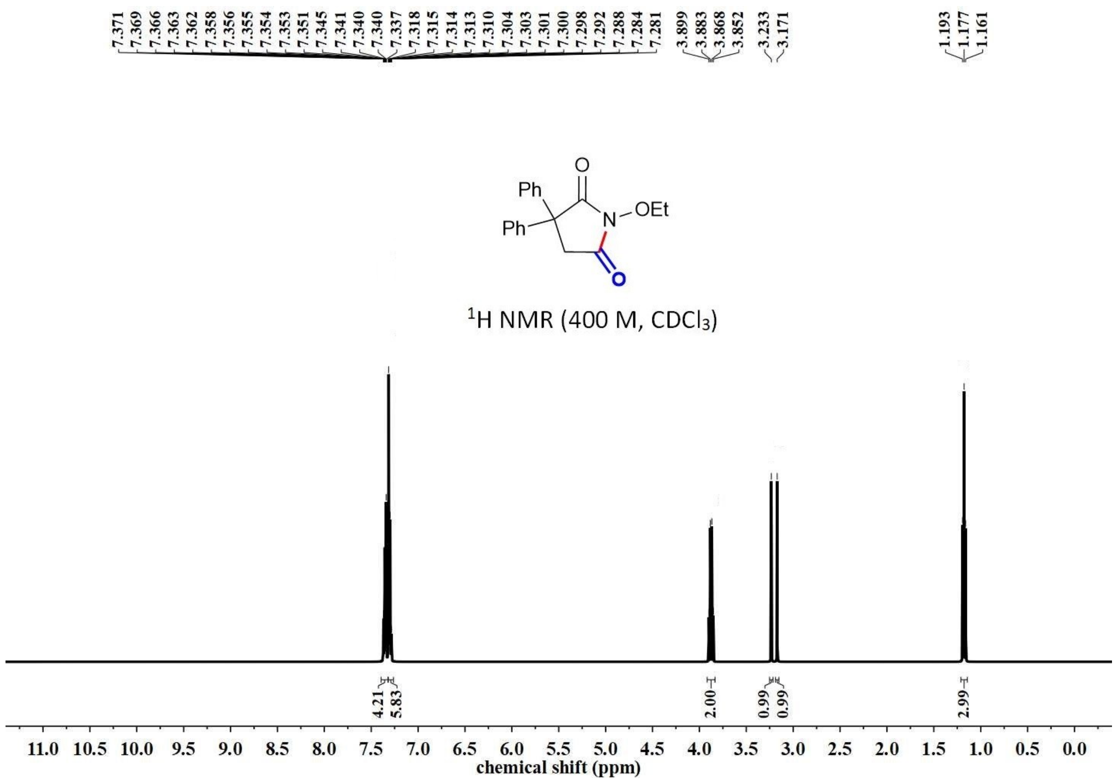

^{1}H NMR (400 M, $CDCl_3$)

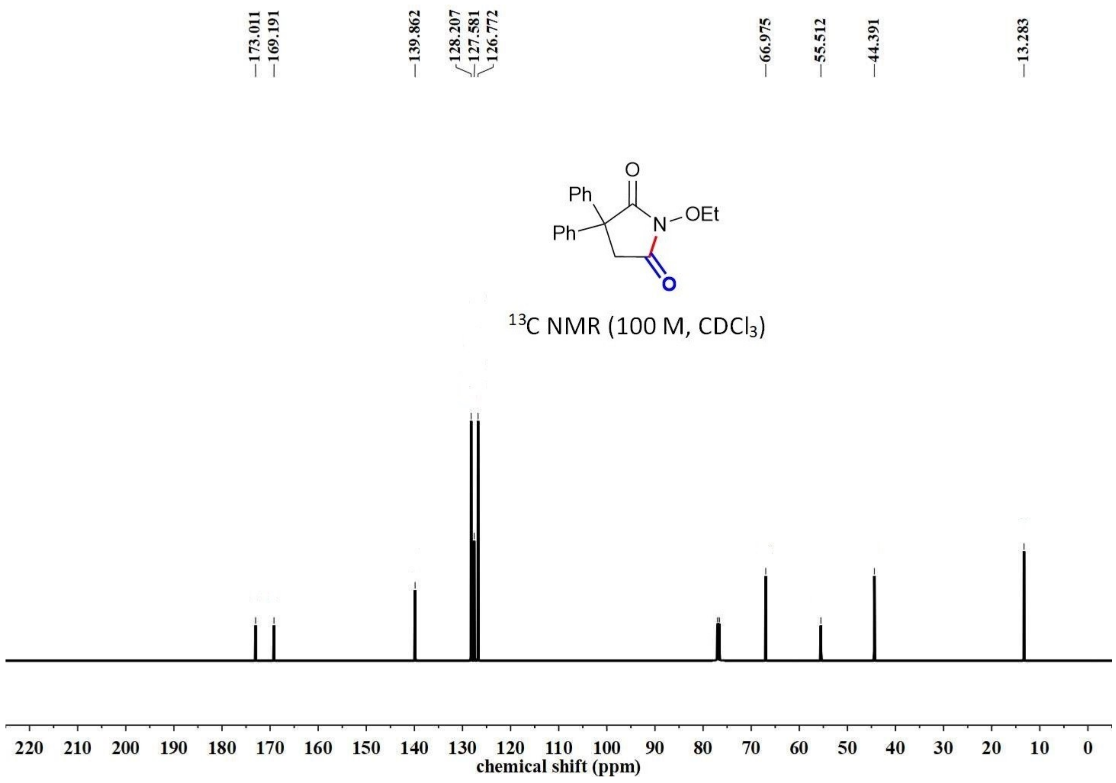

^{13}C NMR (100 M, $CDCl_3$)

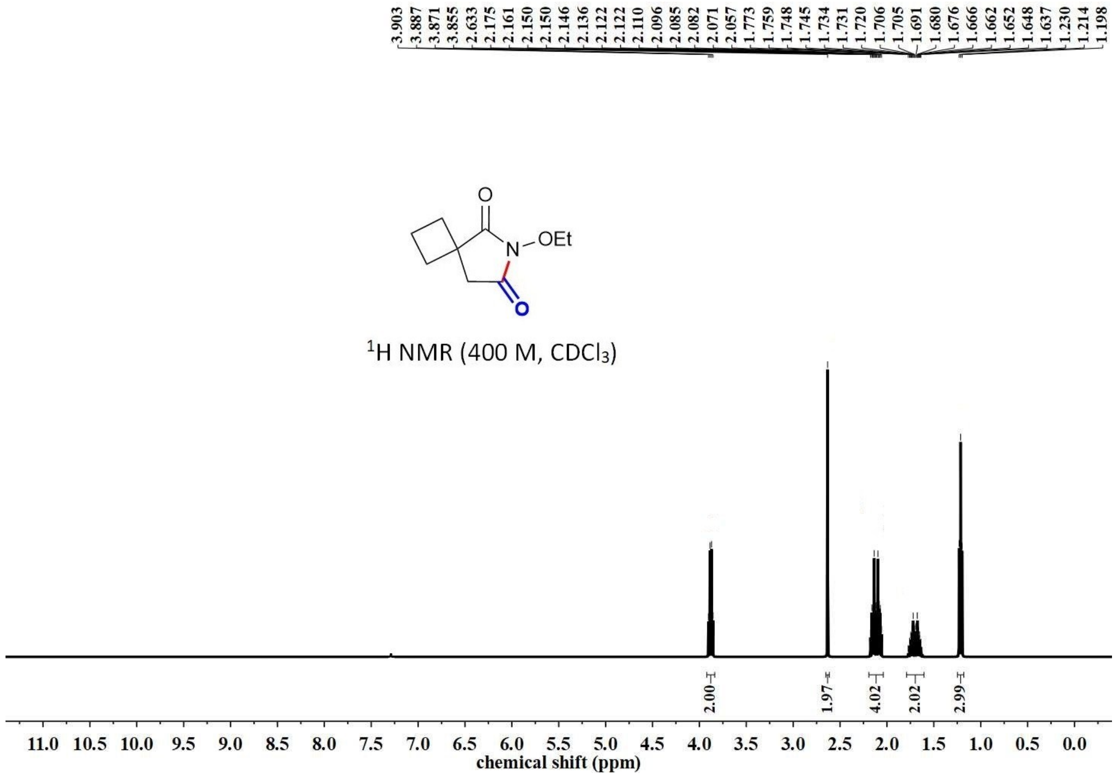
3.903 3.887 3.871 3.855 2.633 2.175 2.161 2.150 2.150 2.146 2.136 2.122 2.122 2.110 2.096 2.085 2.082 2.071 2.057 1.773 1.759 1.748 1.745 1.734 1.731 1.720 1.706 1.705 1.691 1.680 1.676 1.666 1.662 1.652 1.648 1.637 1.230 1.214 1.198
O
N
OEt
O
^{1}H NMR (400 M, $CDCl_3$)
2.00
1.97
4.02
2.02
2.99
11.0 10.5 10.0 9.5 9.0 8.5 8.0 7.5 7.0 6.5 6.0 5.5 5.0 4.5 4.0 3.5 3.0 2.5 2.0 1.5 1.0 0.5 0.0
chemical shift (ppm)

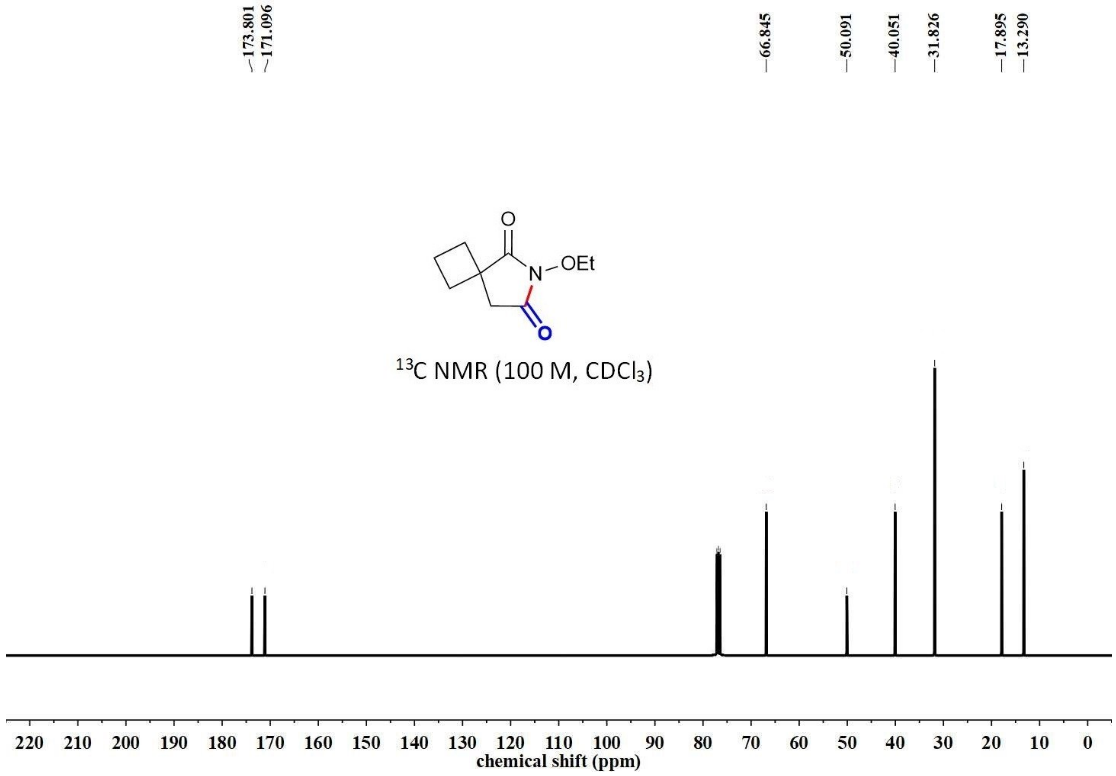
173.801
171.096
66.845
50.091
40.051
31.826
17.895
13.290
O
N
OEt
O
^{13}C NMR (100 M, $CDCl_3$)
220 210 200 190 180 170 160 150 140 130 120 110 100 90 80 70 60 50 40 30 20 10 0
chemical shift (ppm)

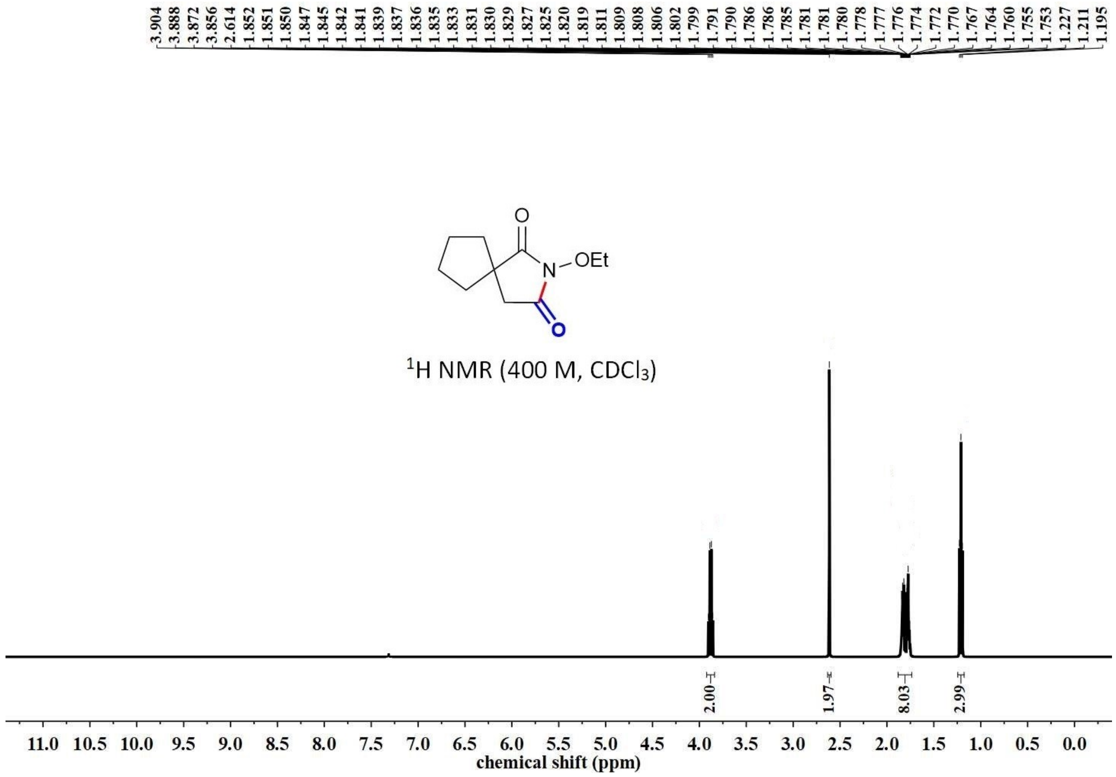
3.904 3.888 3.872 3.856 2.614 1.852 1.851 1.850 1.847 1.845 1.842 1.841 1.839 1.837 1.836 1.835 1.833 1.831 1.830 1.829 1.827 1.825 1.820 1.819 1.811 1.809 1.808 1.806 1.802 1.799 1.791 1.790 1.786 1.786 1.785 1.781 1.781 1.780 1.778 1.777 1.776 1.774 1.772 1.770 1.767 1.764 1.760 1.755 1.753 1.227 1.211 1.195
O
N
OEt
O
¹H NMR (400 M, CDCl₃)
2.00
1.97
8.03
2.99
11.0 10.5 10.0 9.5 9.0 8.5 8.0 7.5 7.0 6.5 6.0 5.5 5.0 4.5 4.0 3.5 3.0 2.5 2.0 1.5 1.0 0.5 0.0
chemical shift (ppm)

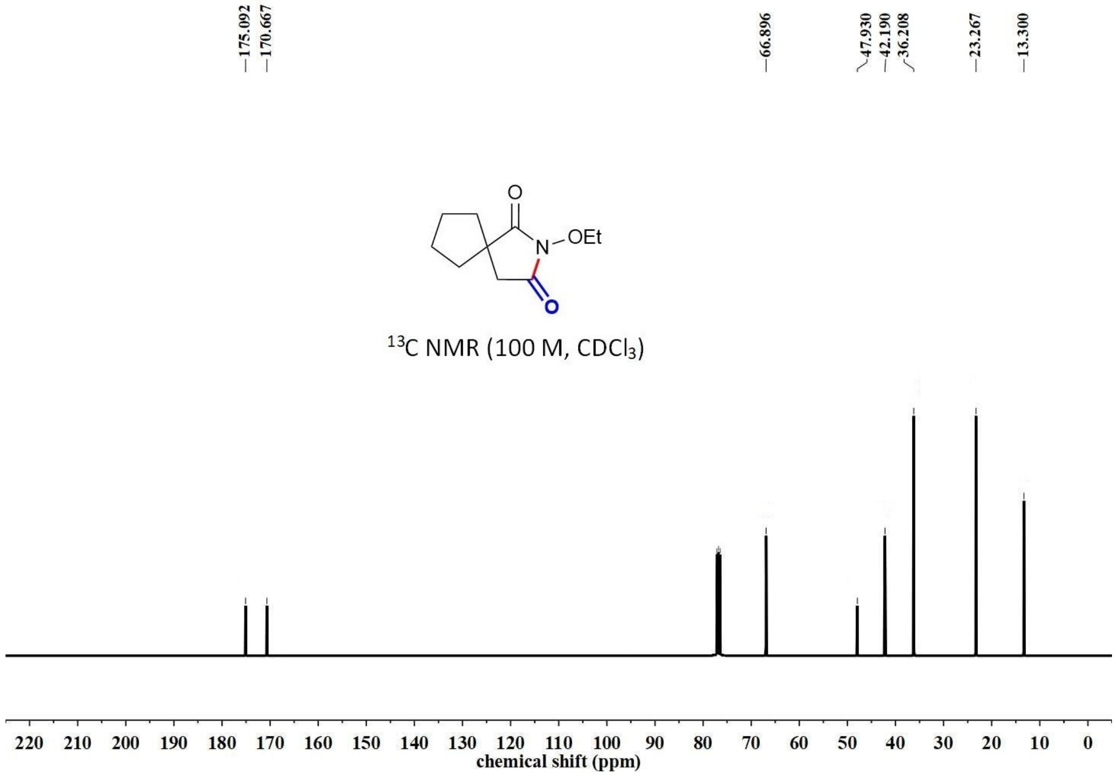
175.092
170.667
66.896
47.930
42.190
36.208
23.267
13.300
O
N
OEt
O
¹³C NMR (100 M, CDCl₃)
220 210 200 190 180 170 160 150 140 130 120 110 100 90 80 70 60 50 40 30 20 10 0
chemical shift (ppm)

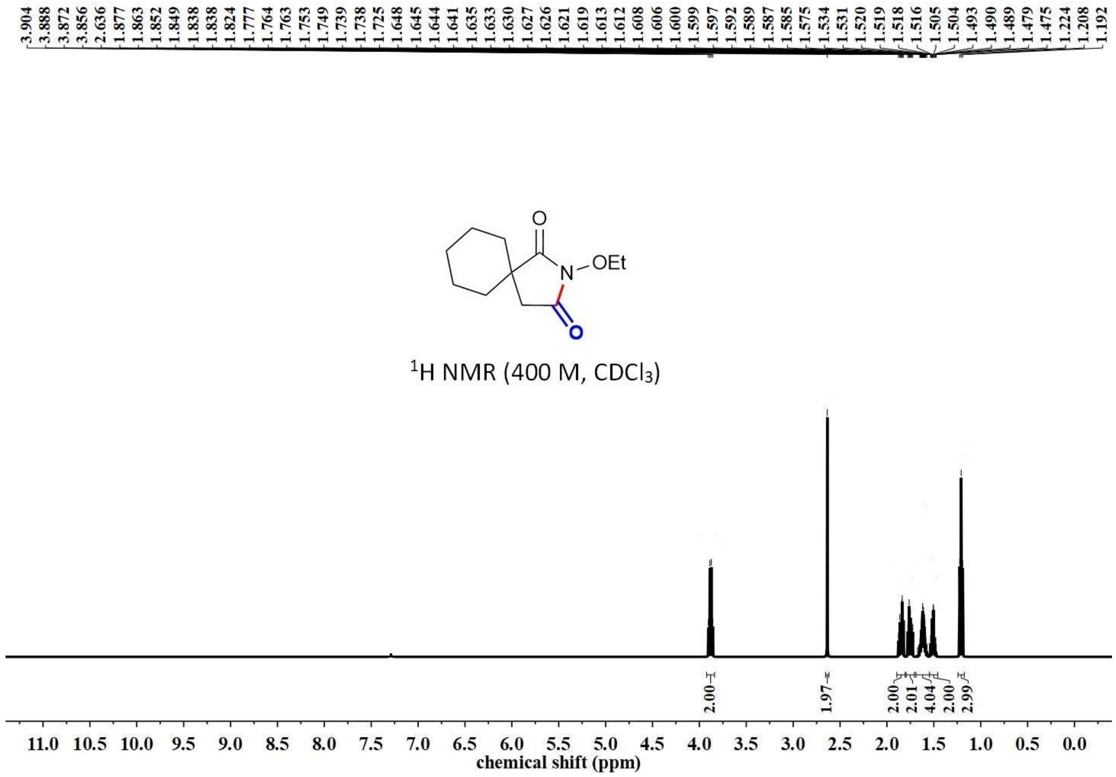

OEt
1H NMR (400 M, CDCl3)
chemical shift (ppm)

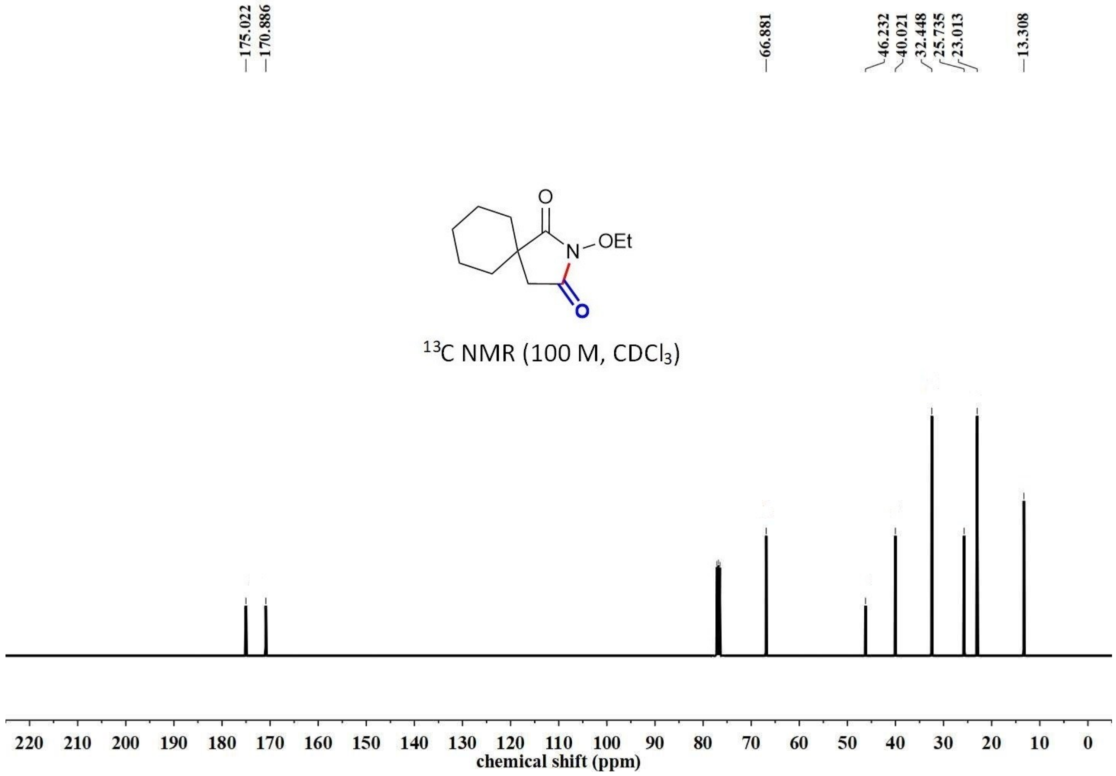

OEt
13C NMR (100 M, CDCl3)
chemical shift (ppm)

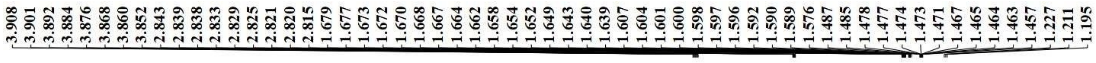

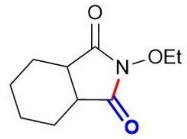

^{1}H NMR (400 M, $CDCl_3$)

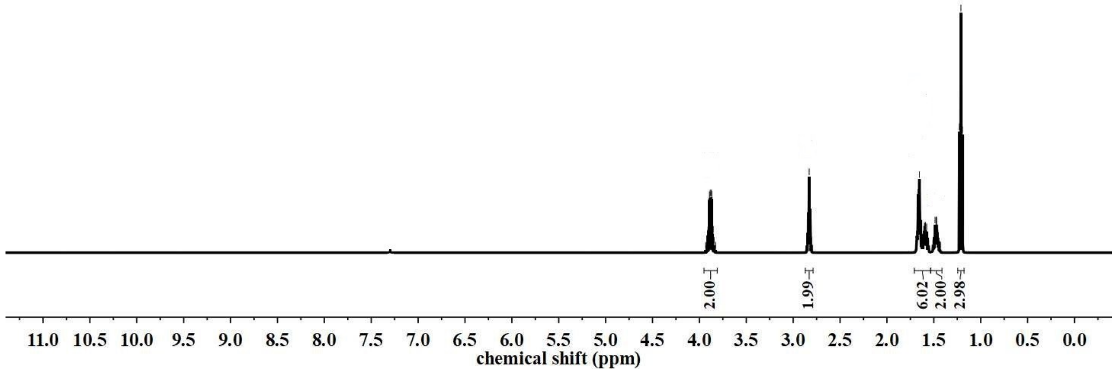

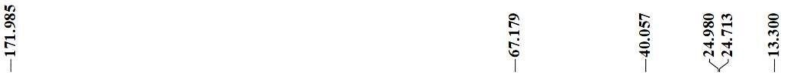

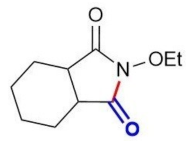

^{13}C NMR (100 M, $CDCl_3$)

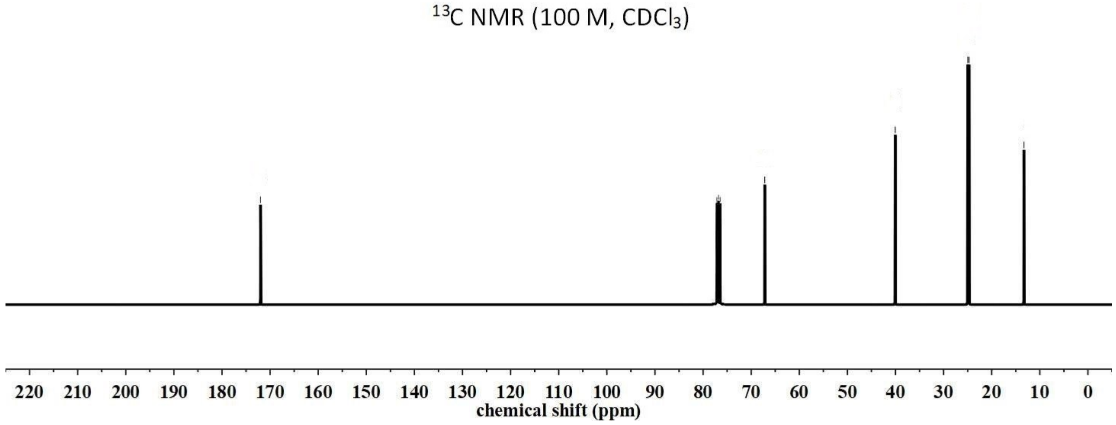

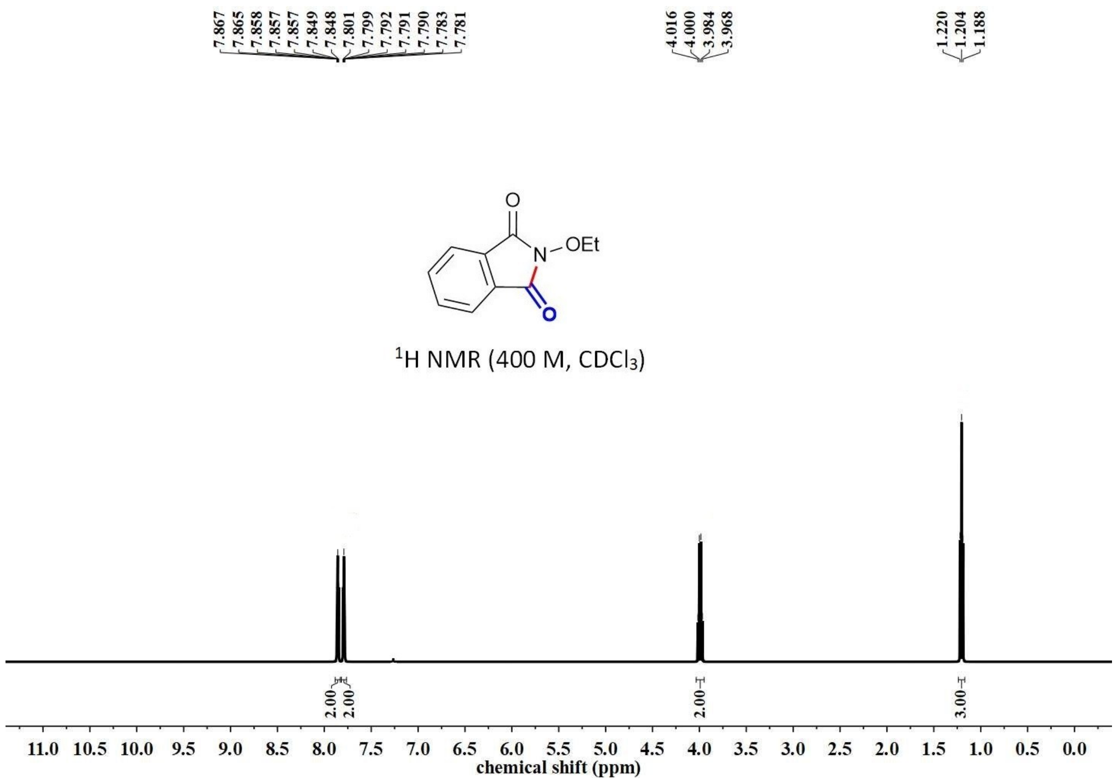
7.867
7.865
7.858
7.857
7.857
7.849
7.848
7.801
7.799
7.792
7.791
7.790
7.783
7.781
4.016
4.000
3.984
3.968
1.220
1.204
1.188
O
N
OEt
O
1H NMR (400 M, CDCl3)
2.00
2.00
2.00
3.00
11.0 10.5 10.0 9.5 9.0 8.5 8.0 7.5 7.0 6.5 6.0 5.5 5.0 4.5 4.0 3.5 3.0 2.5 2.0 1.5 1.0 0.5 0.0
chemical shift (ppm)

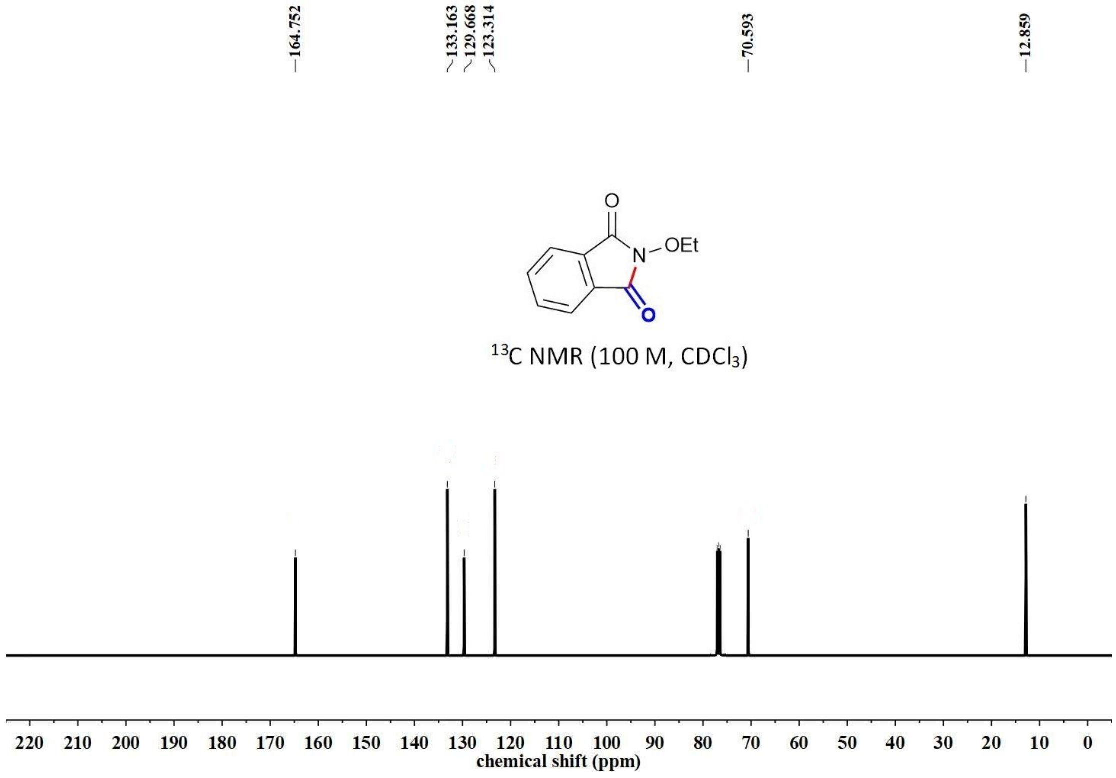
164.752
133.163
129.668
123.314
70.593
12.859
O
N
OEt
O
13C NMR (100 M, CDCl3)
220 210 200 190 180 170 160 150 140 130 120 110 100 90 80 70 60 50 40 30 20 10 0
chemical shift (ppm)

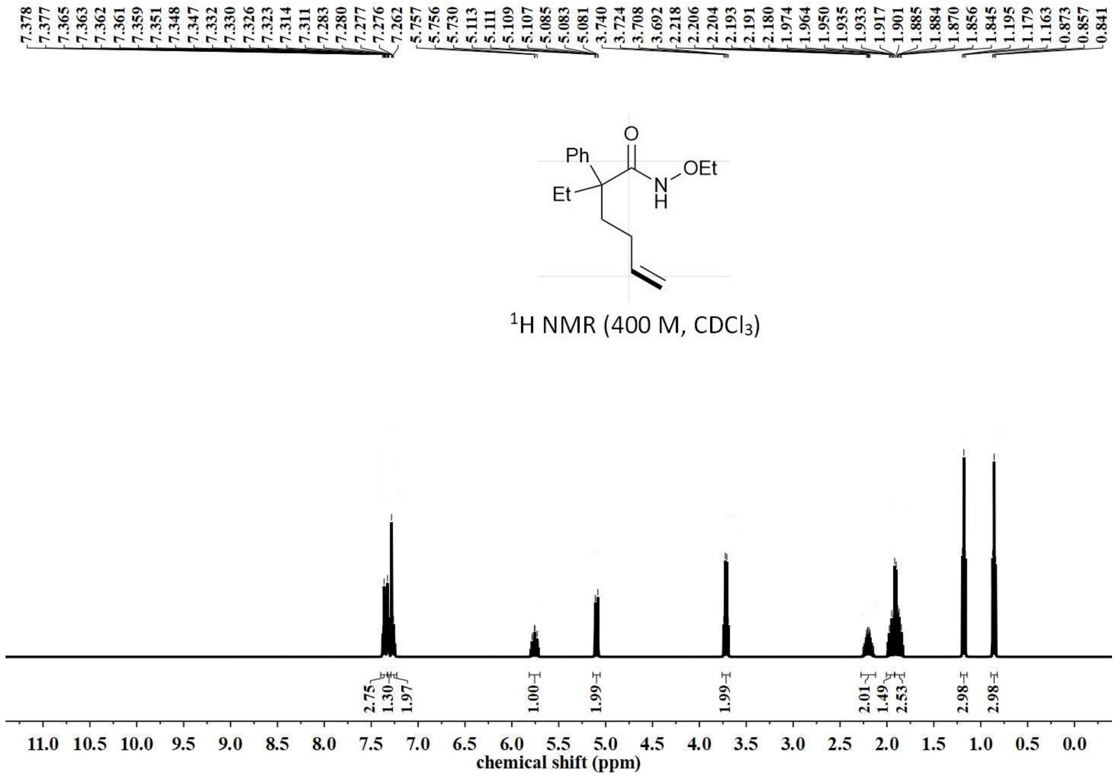

^{1}H NMR (400 M, $CDCl_3$)

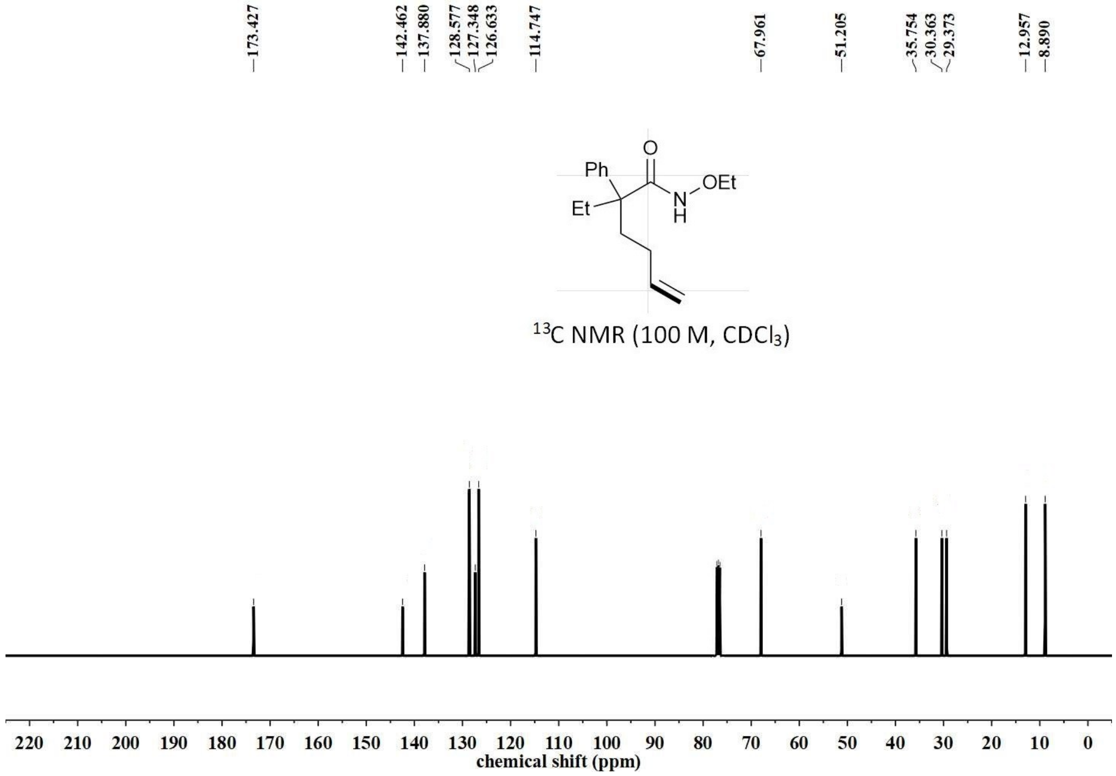

^{13}C NMR (100 M, $CDCl_3$)

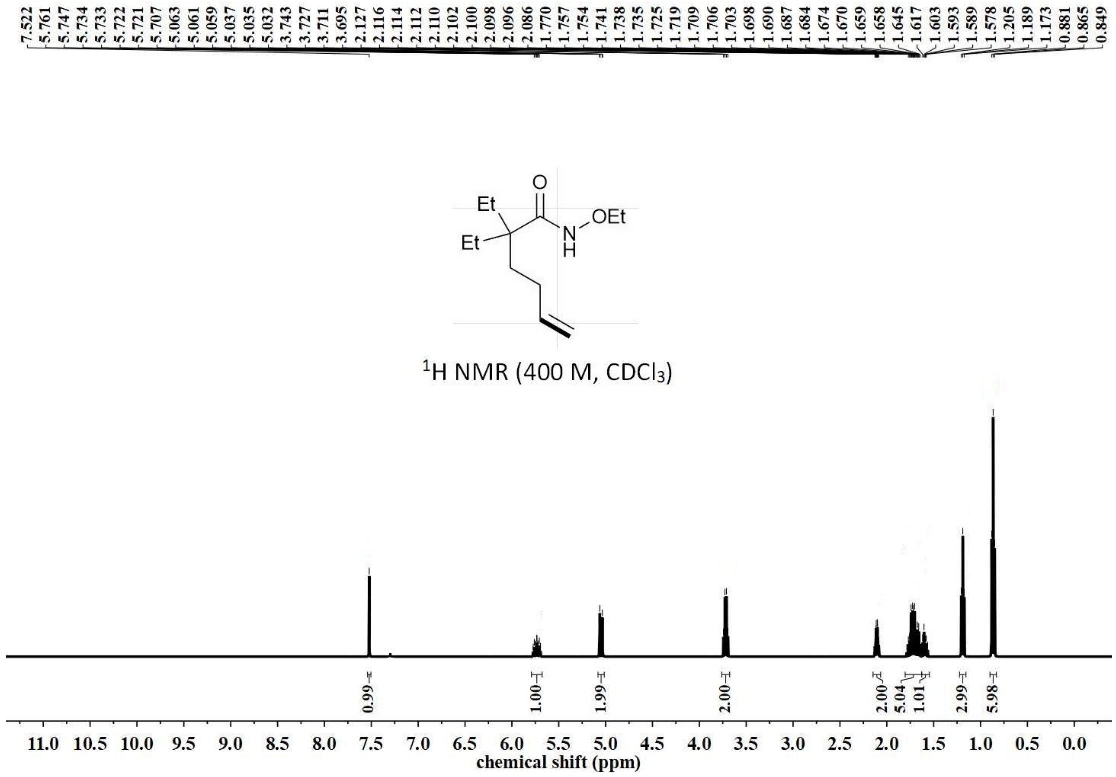
7.522 5.761 5.747 5.734 5.733 5.722 5.721 5.707 5.063 5.061 5.059 5.037 5.035 5.032 3.743 3.727 3.711 3.695 2.127 2.116 2.114 2.112 2.110 2.102 2.100 2.098 2.096 2.086 1.770 1.757 1.754 1.741 1.738 1.735 1.725 1.719 1.709 1.706 1.703 1.698 1.690 1.687 1.684 1.674 1.670 1.659 1.658 1.645 1.617 1.603 1.593 1.589 1.578 1.205 1.189 1.173 0.881 0.865 0.849
Et
Et
O
N
H
OEt
^{1}H NMR (400 M, CDCl$_3$)
0.99
1.00
1.99
2.00
2.00
5.04
1.01
2.99
5.98
11.0 10.5 10.0 9.5 9.0 8.5 8.0 7.5 7.0 6.5 6.0 5.5 5.0 4.5 4.0 3.5 3.0 2.5 2.0 1.5 1.0 0.5 0.0
chemical shift (ppm)

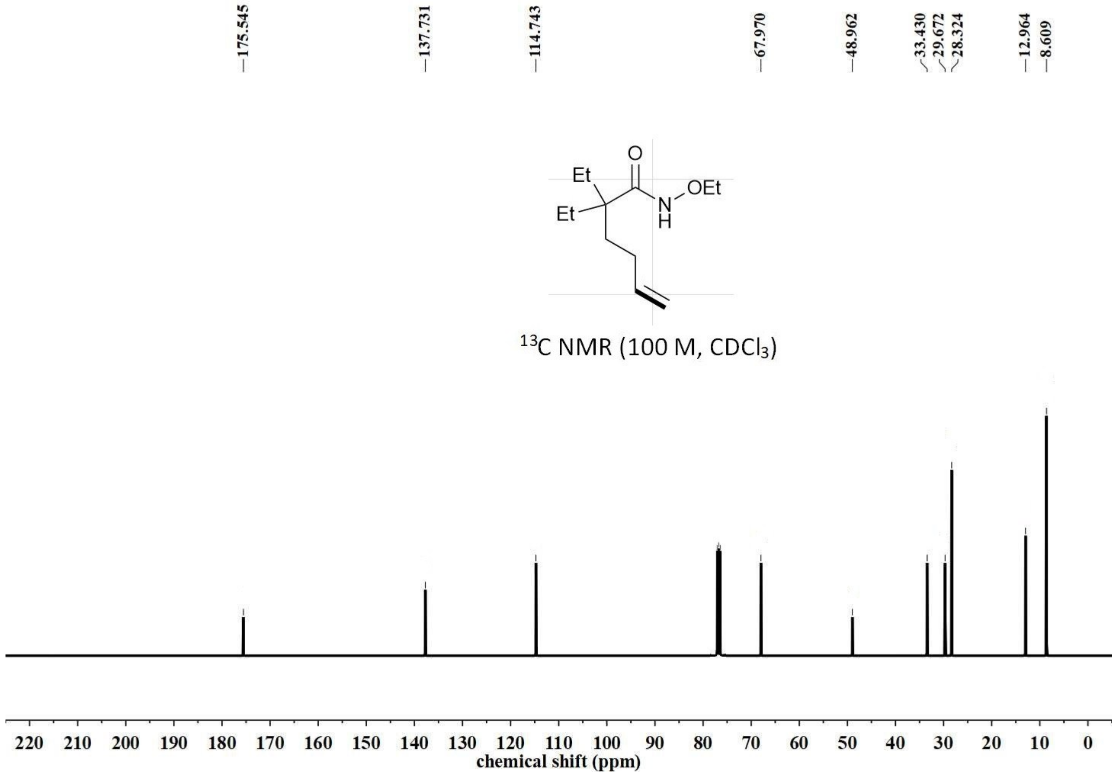
175.545
137.731
114.743
67.970
48.962
33.430
29.672
28.324
12.964
8.609
Et
Et
O
N
H
OEt
^{13}C NMR (100 M, CDCl$_3$)
220 210 200 190 180 170 160 150 140 130 120 110 100 90 80 70 60 50 40 30 20 10 0
chemical shift (ppm)

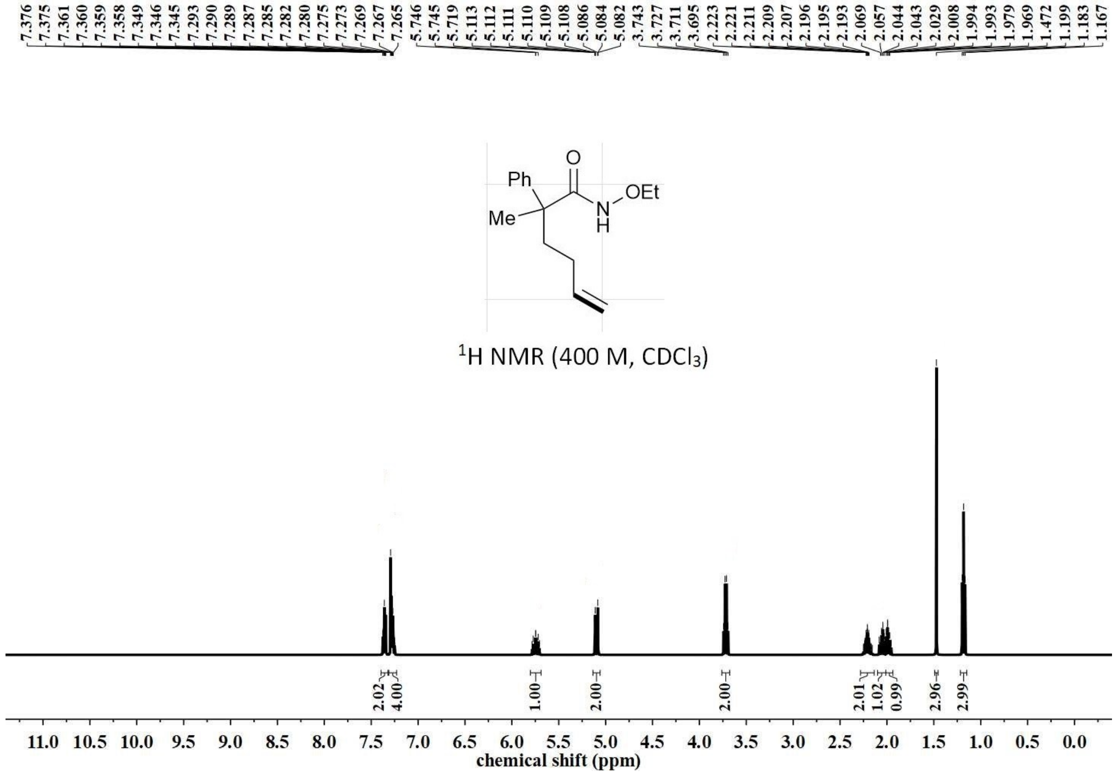

^{1}H NMR (400 M, $CDCl_3$)

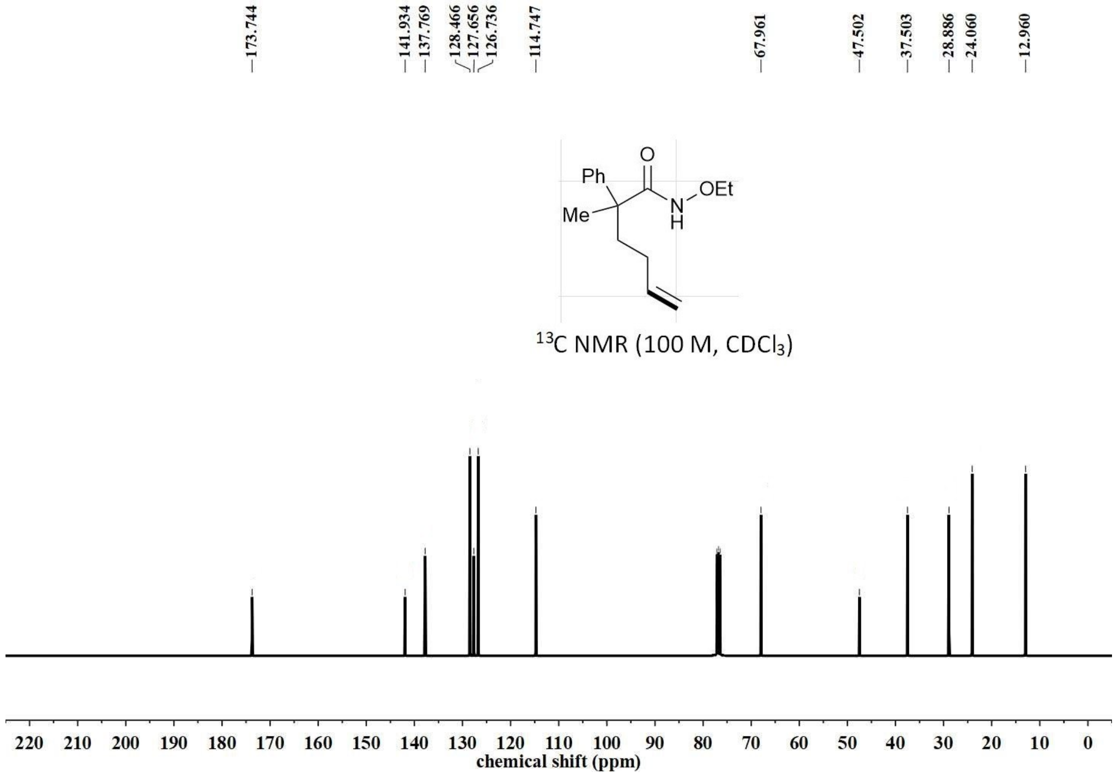

^{13}C NMR (100 M, $CDCl_3$)

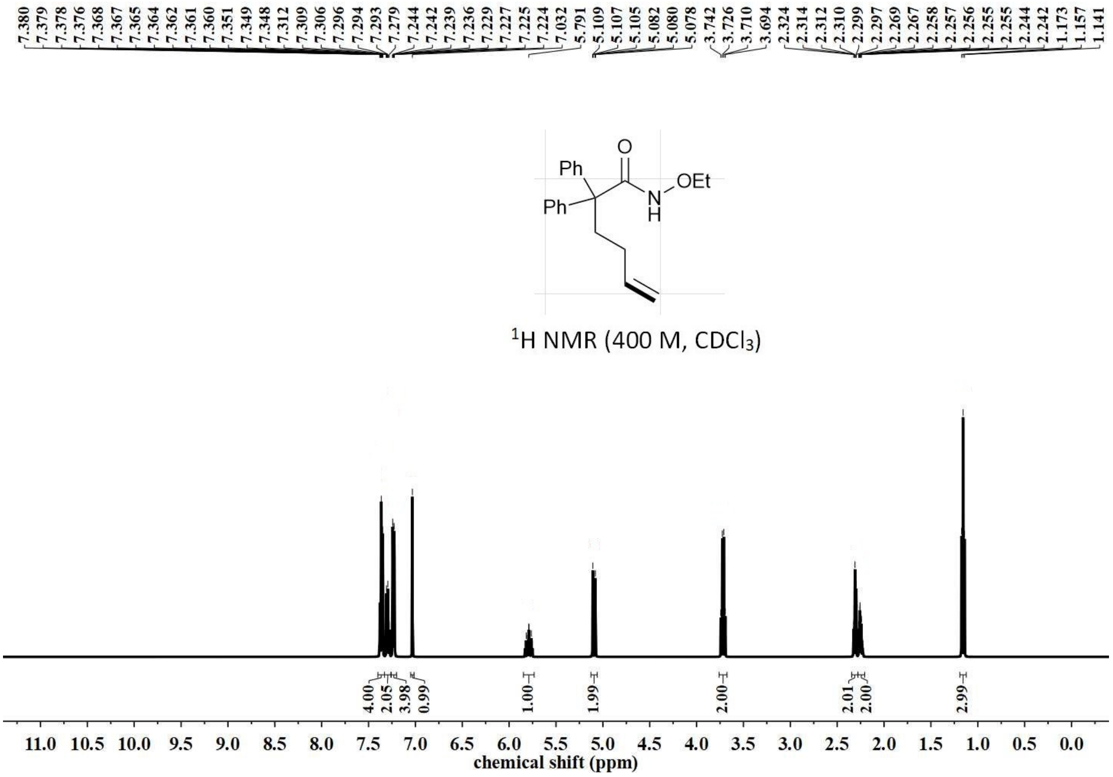
7.380 7.379 7.378 7.376 7.368 7.367 7.365 7.364 7.362 7.361 7.360 7.351 7.349 7.348 7.312 7.309 7.306 7.296 7.294 7.293 7.279 7.244 7.242 7.239 7.236 7.229 7.227 7.225 7.224 7.032 5.791 5.109 5.107 5.105 5.082 5.080 5.078 3.742 3.726 3.710 3.694 2.324 2.314 2.312 2.310 2.299 2.297 2.269 2.267 2.258 2.257 2.256 2.255 2.255 2.244 2.242 1.173 1.157 1.141
Ph
Ph
O
N
H
OEt
^{1}H NMR (400 M, CDCl$_3$)
4.00 2.05 3.98 0.99 1.00 1.99 2.00 2.01 2.00 2.99
chemical shift (ppm)

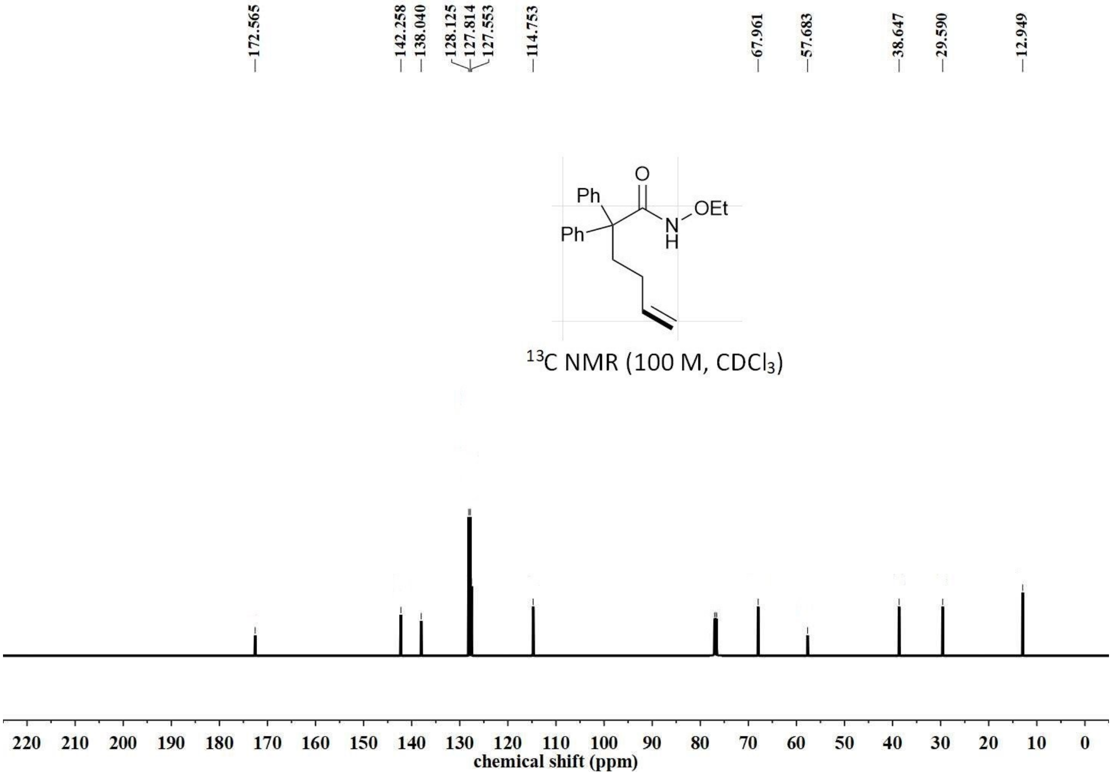
172.565 142.258 138.040 128.125 127.814 127.553 114.753 67.961 57.683 38.647 29.590 12.949
Ph
Ph
O
N
H
OEt
^{13}C NMR (100 M, CDCl$_3$)
chemical shift (ppm)

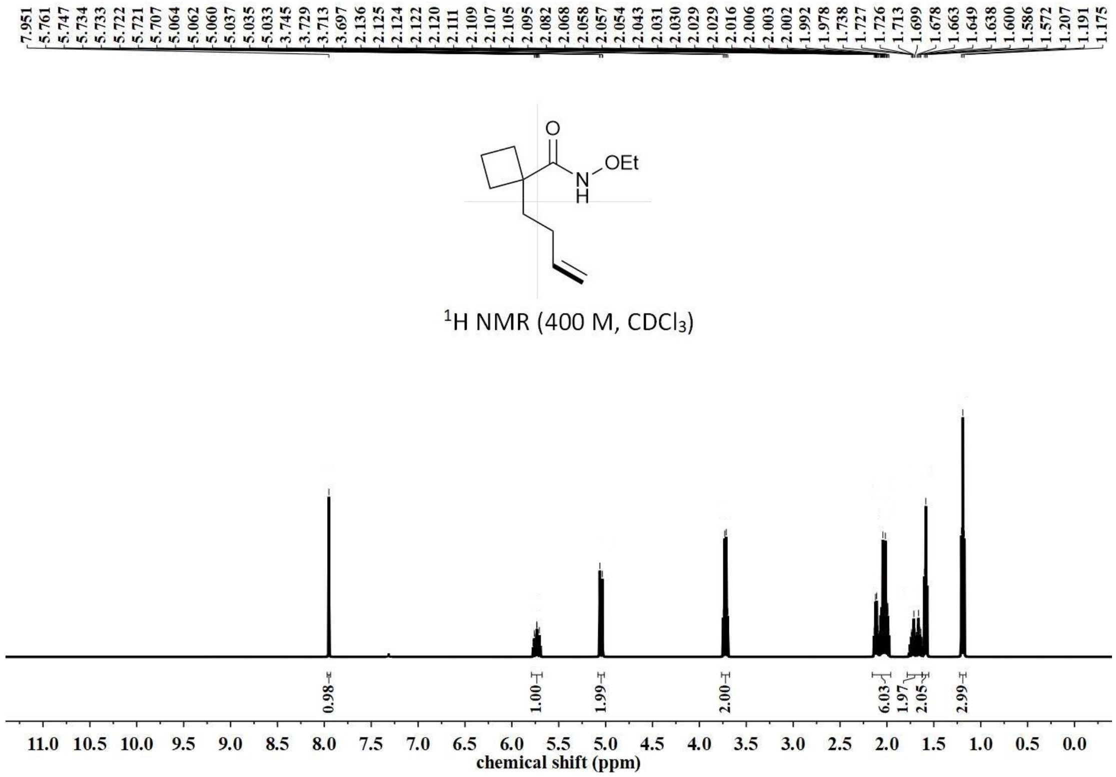

^{1}H NMR (400 M, $CDCl_3$)

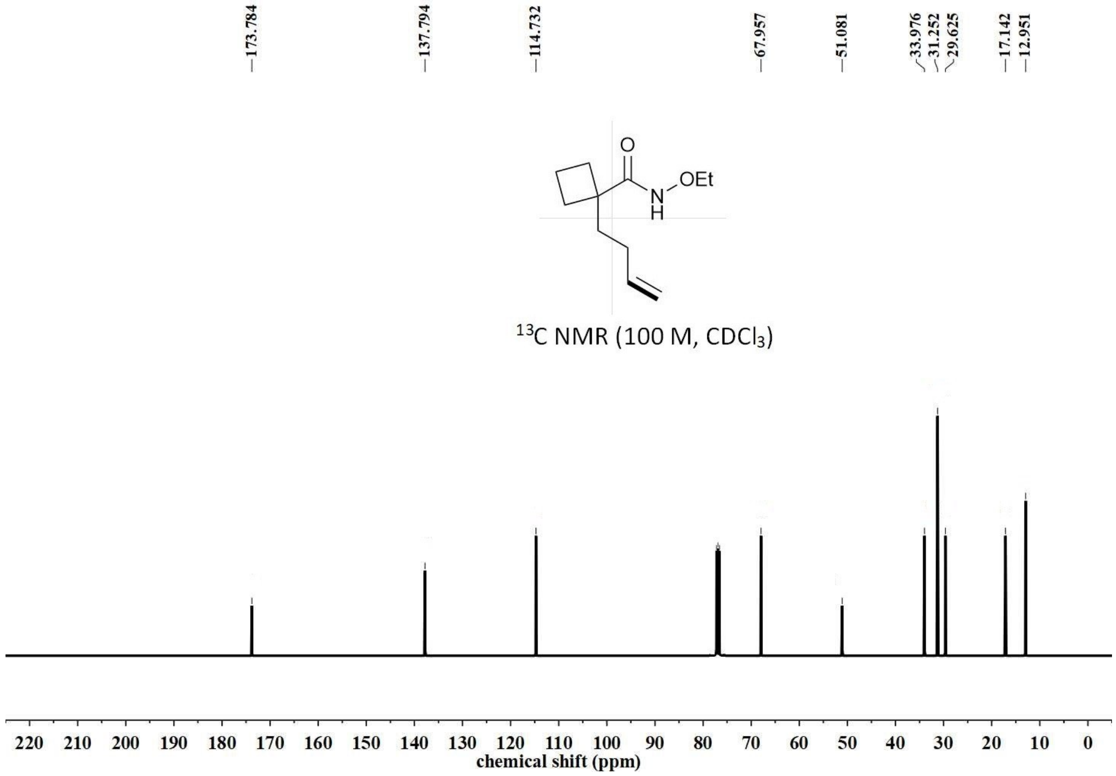

^{13}C NMR (100 M, $CDCl_3$)

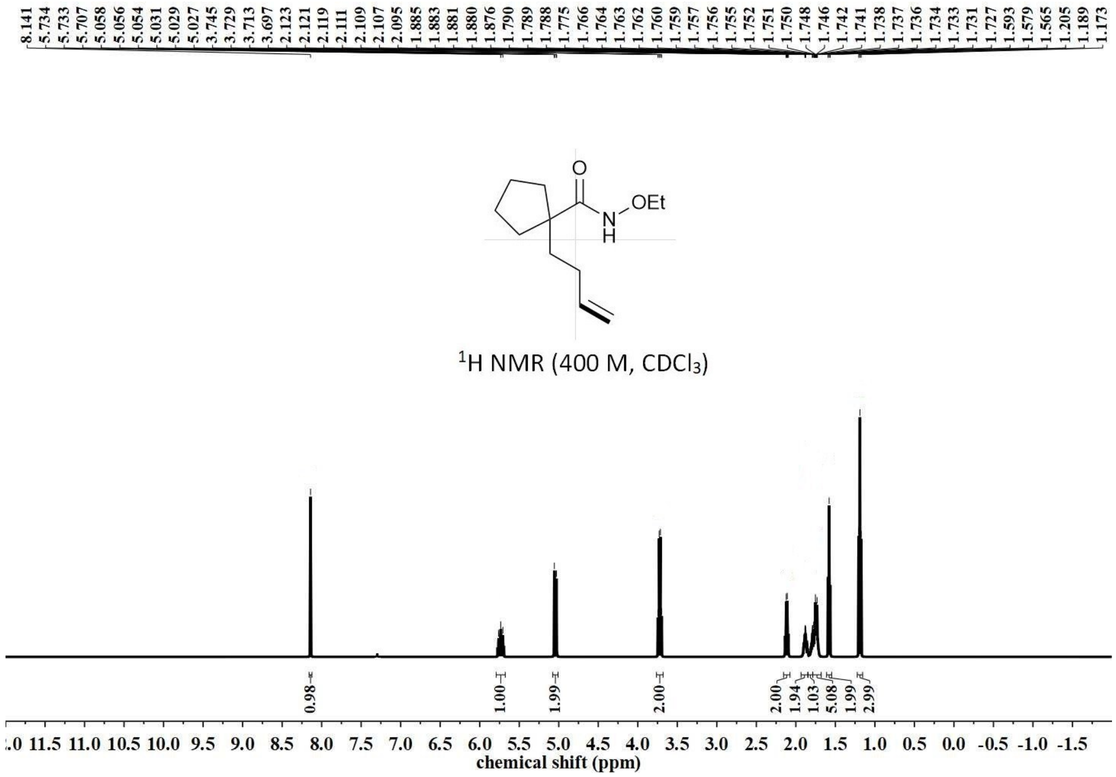
8.141 5.734 5.733 5.707 5.058 5.056 5.054 5.031 5.029 5.027 3.745 3.729 3.713 3.697 2.123 2.121 2.119 2.111 2.109 2.107 2.095 1.885 1.883 1.881 1.880 1.876 1.790 1.789 1.788 1.775 1.766 1.764 1.763 1.762 1.760 1.759 1.757 1.756 1.755 1.752 1.751 1.750 1.748 1.746 1.742 1.741 1.738 1.737 1.736 1.734 1.733 1.731 1.727 1.593 1.579 1.565 1.205 1.189 1.173
O
OEt
N
H
^{1}H NMR (400 M, CDCl$_3$)
0.98
1.00
1.99
2.00
2.00
1.94
1.03
5.08
1.99
2.99
chemical shift (ppm)

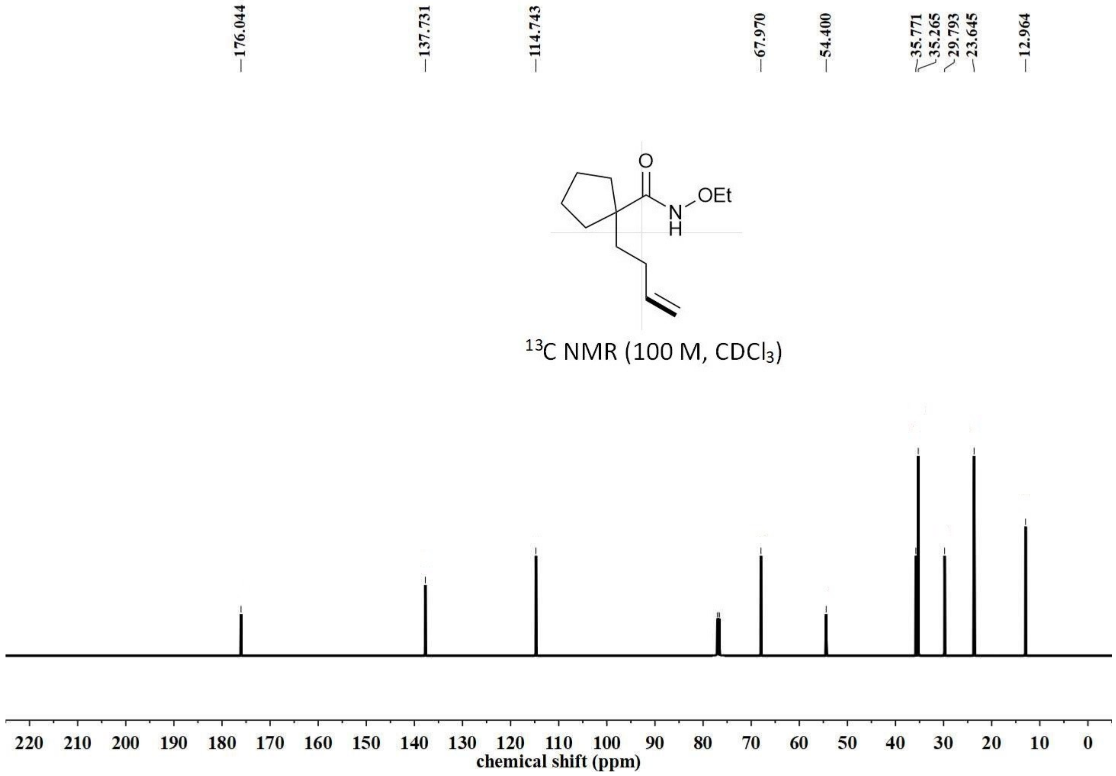
176.044
137.731
114.743
67.970
54.400
35.771
35.265
29.793
23.645
12.964
O
OEt
N
H
^{13}C NMR (100 M, CDCl$_3$)
220 210 200 190 180 170 160 150 140 130 120 110 100 90 80 70 60 50 40 30 20 10 0
chemical shift (ppm)

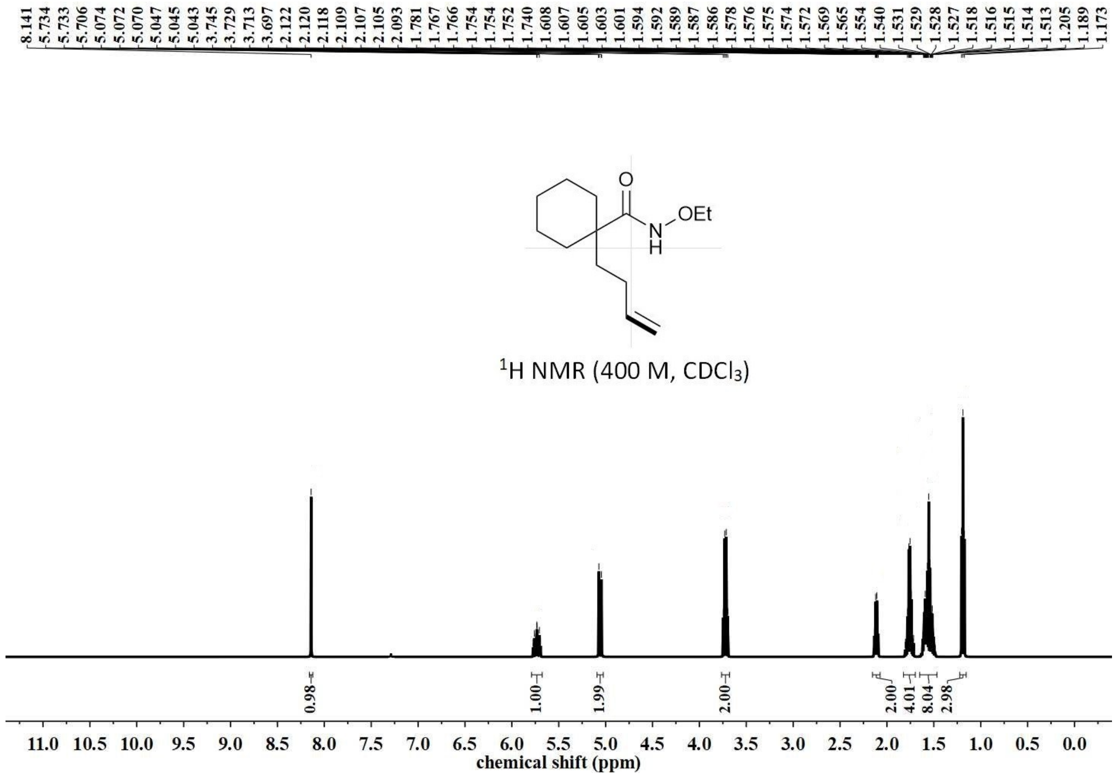

^{1}H NMR (400 M, $CDCl_3$)

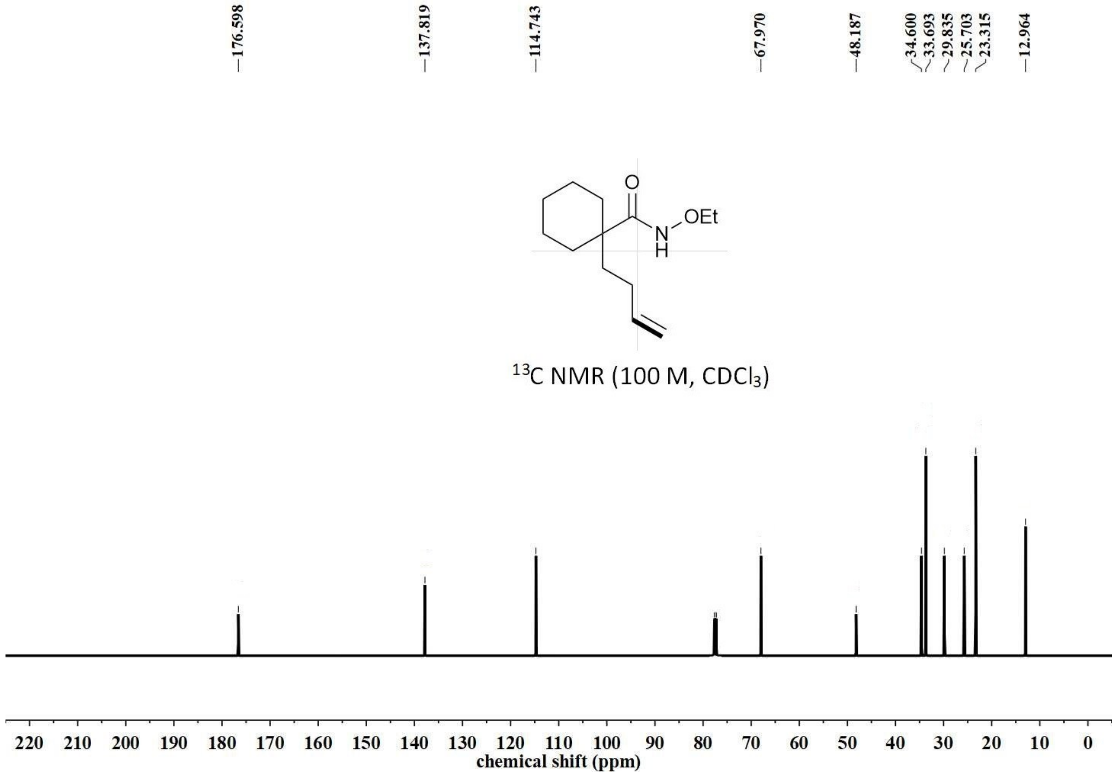

^{13}C NMR (100 M, $CDCl_3$)

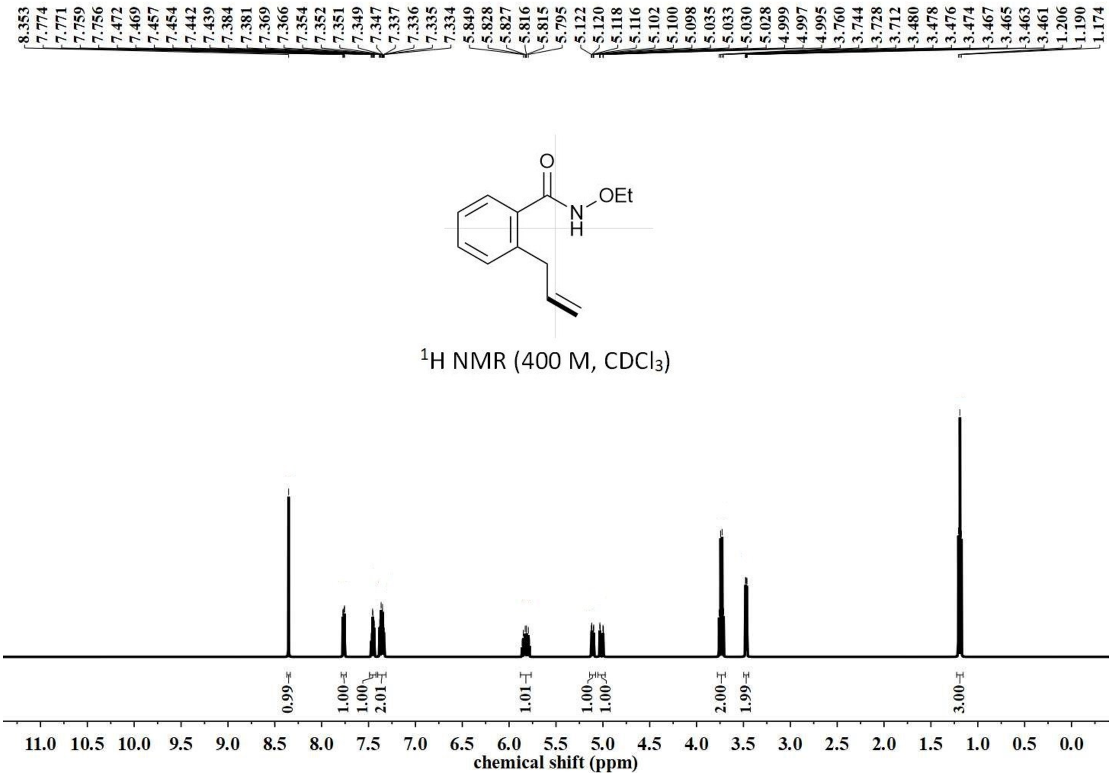

^{1}H NMR (400 M, $CDCl_3$)

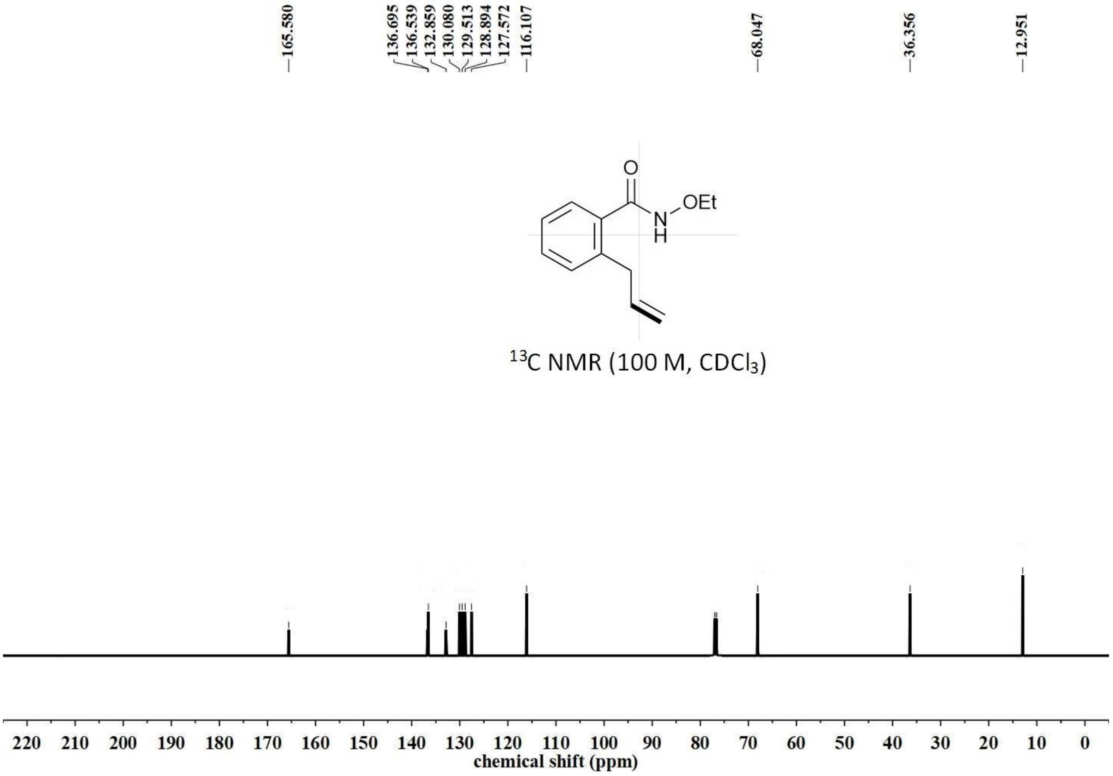

^{13}C NMR (100 M, $CDCl_3$)

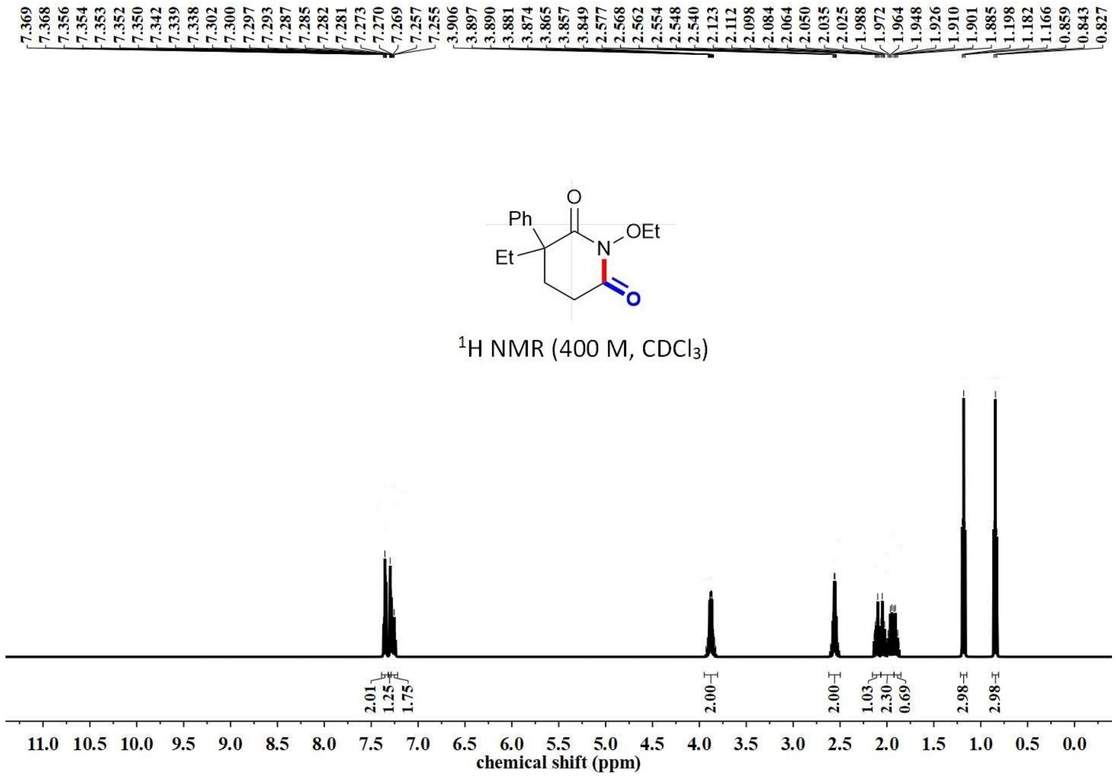
7.369 7.368 7.356 7.354 7.353 7.352 7.350 7.342 7.339 7.338 7.302 7.300 7.297 7.293 7.287 7.285 7.282 7.281 7.273 7.270 7.269 7.257 7.255
3.906 3.897 3.890 3.881 3.874 3.865 3.857 3.849
2.577 2.568 2.562 2.554 2.548 2.540
2.123 2.112 2.098 2.084 2.064 2.050 2.035 2.025 1.988 1.972 1.964 1.948 1.926 1.910 1.901 1.885
1.198 1.182 1.166 0.859 0.843 0.827
Ph
O
N
OEt
Et
O
^{1}H NMR (400 M, CDCl$_3$)
2.01 1.25 1.75 2.00 2.00 1.03 2.30 0.69 2.98 2.98
11.0 10.5 10.0 9.5 9.0 8.5 8.0 7.5 7.0 6.5 6.0 5.5 5.0 4.5 4.0 3.5 3.0 2.5 2.0 1.5 1.0 0.5 0.0
chemical shift (ppm)

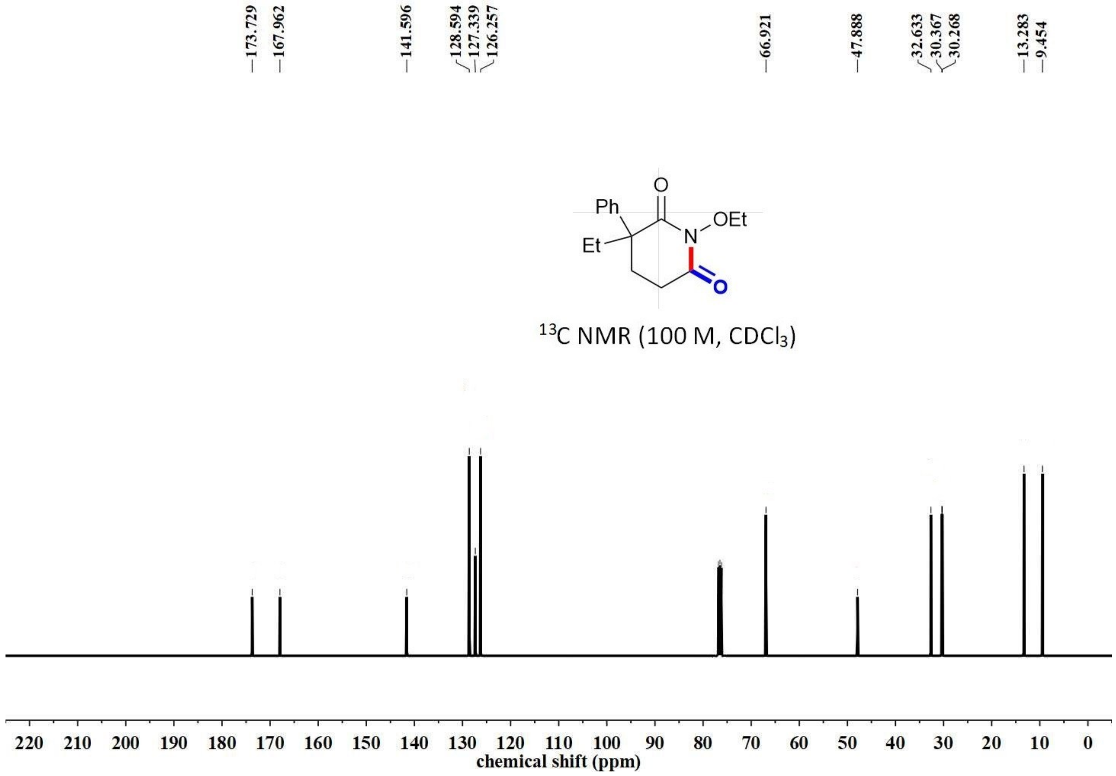
173.729 167.962 141.596 128.594 127.339 126.257 66.921 47.888 32.633 30.367 30.268 13.283 9.454
Ph
O
N
OEt
Et
O
^{13}C NMR (100 M, CDCl$_3$)
220 210 200 190 180 170 160 150 140 130 120 110 100 90 80 70 60 50 40 30 20 10 0
chemical shift (ppm)

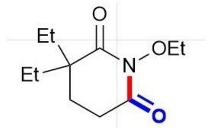

^{1}H NMR (400 M, $CDCl_3$)

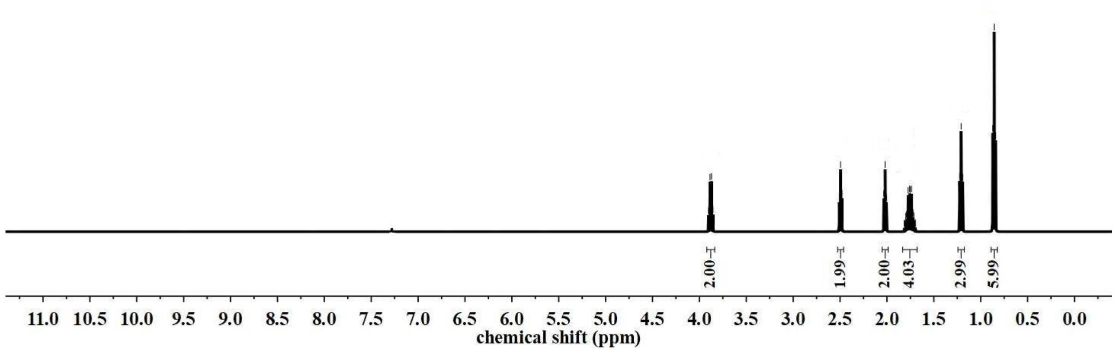

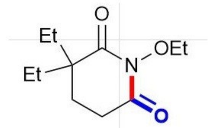

^{13}C NMR (100 M, $CDCl_3$)

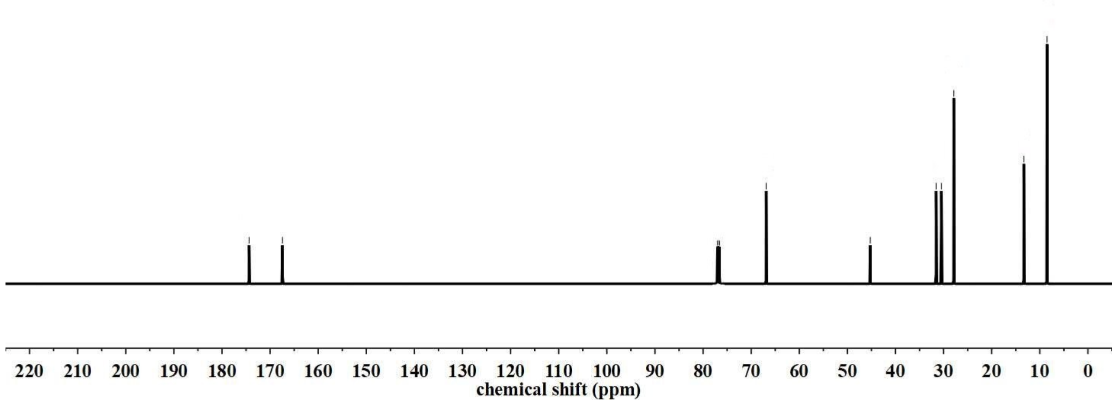

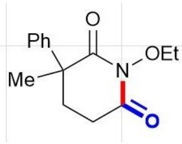

^{1}H NMR (400 M, $CDCl_3$)

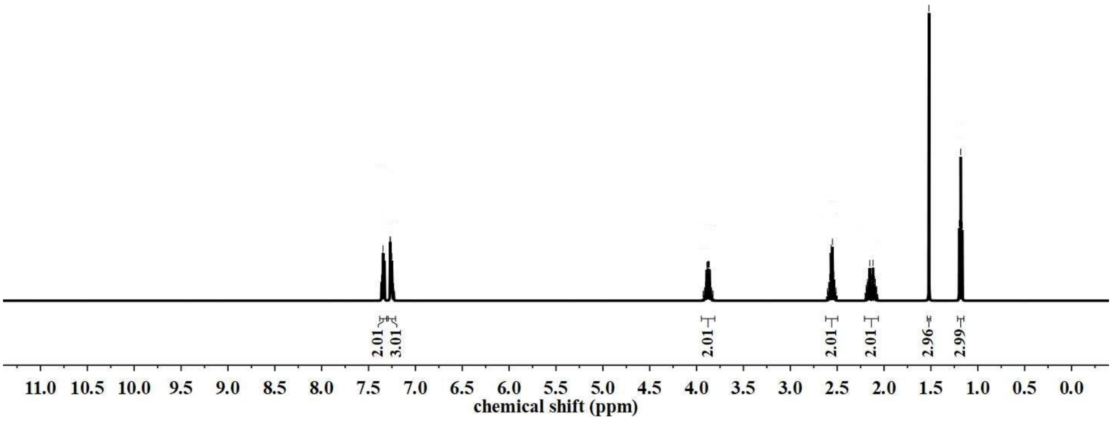

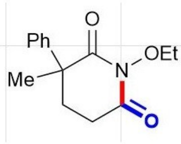

^{13}C NMR (100 M, $CDCl_3$)

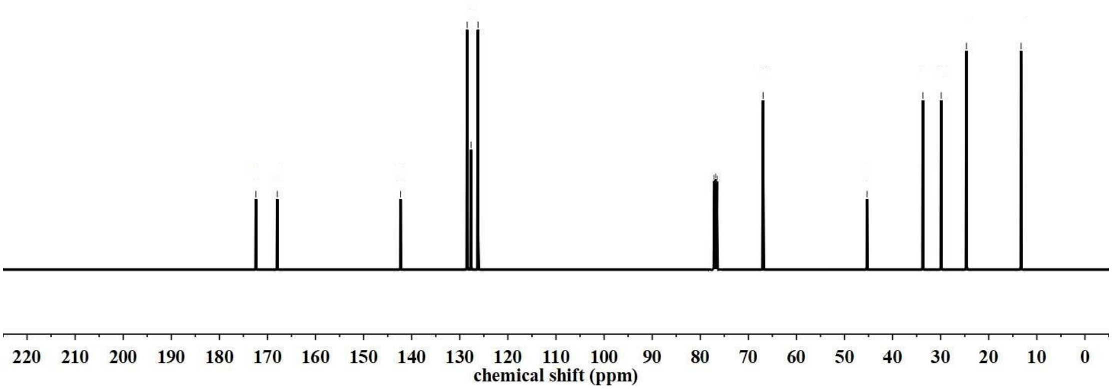

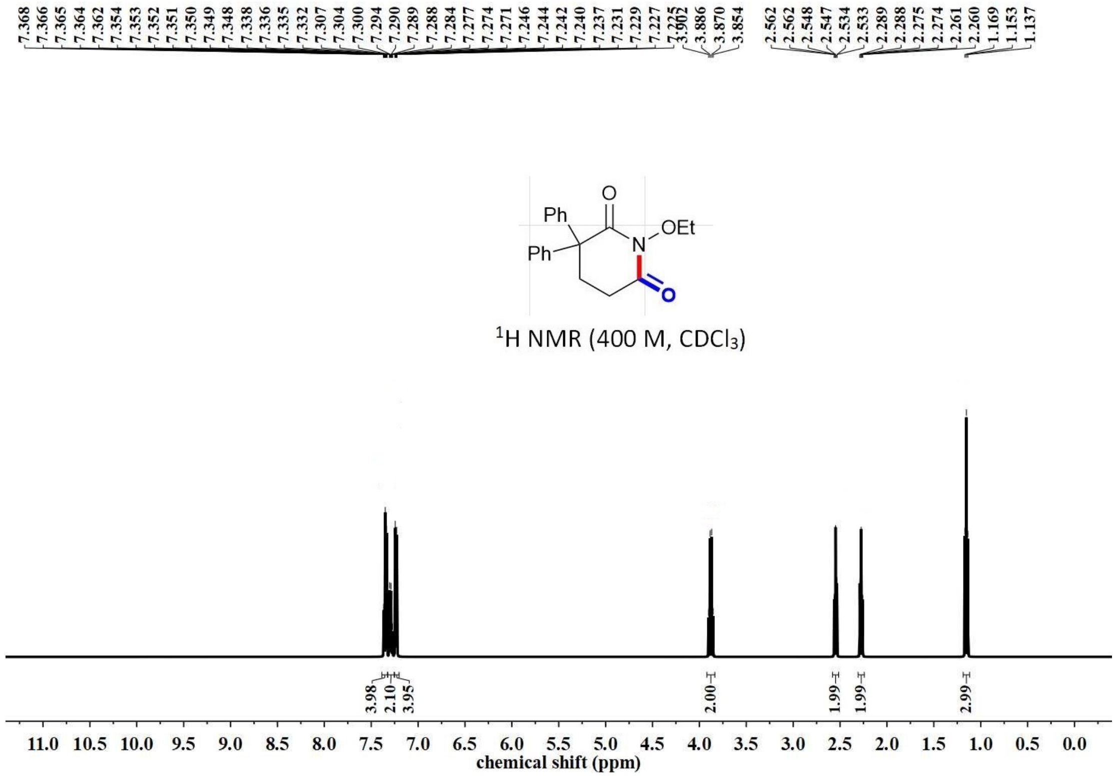
Ph
Ph
O
N
OEt
O
^{1}H NMR (400 M, CDCl$_3$)
chemical shift (ppm)

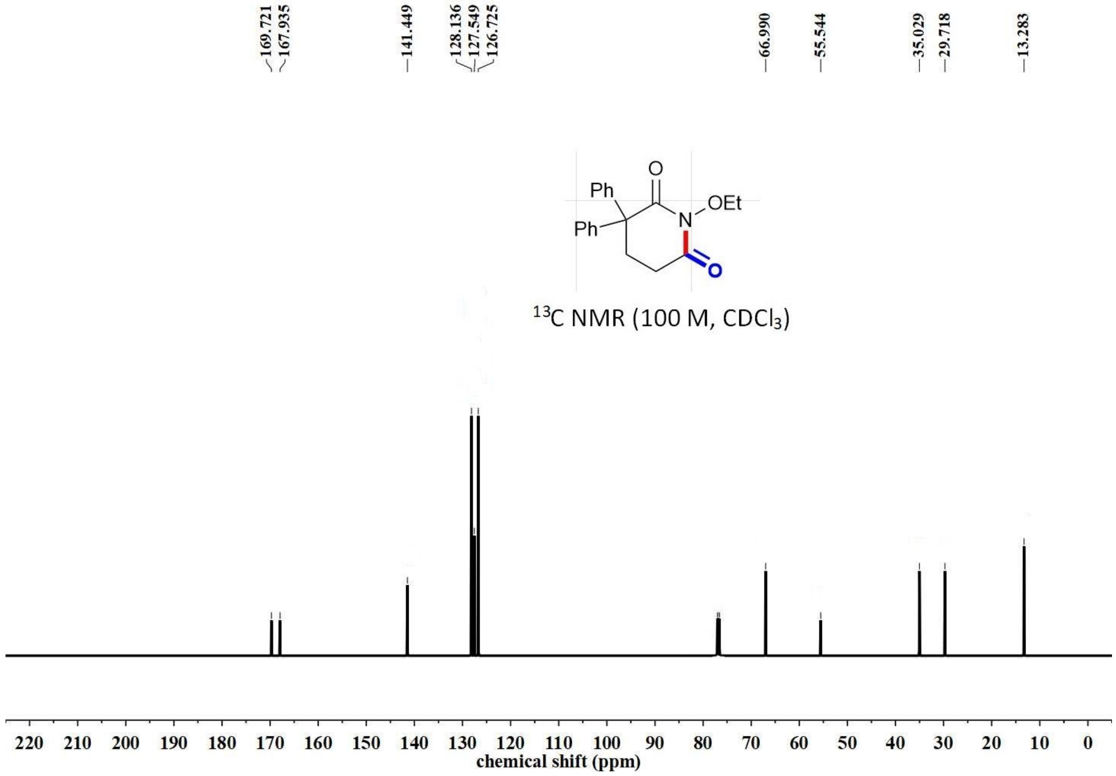
Ph
Ph
O
N
OEt
O
^{13}C NMR (100 M, CDCl$_3$)
chemical shift (ppm)

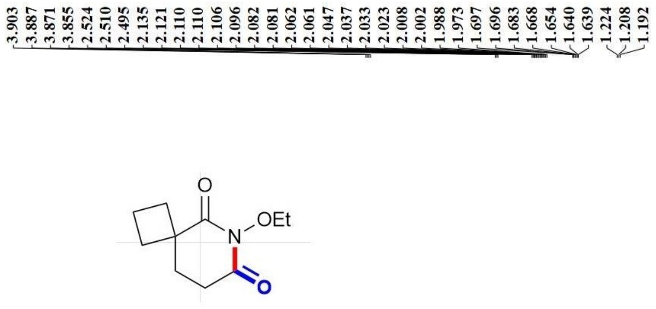

^1H NMR (400 M, $CDCl_3$)

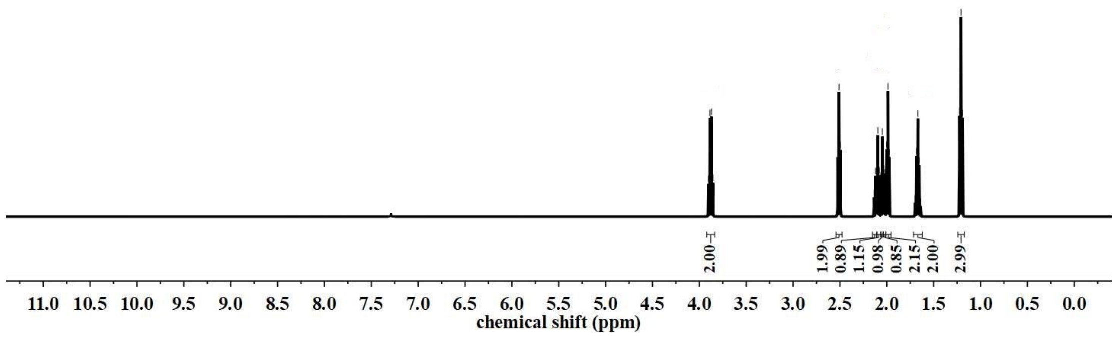

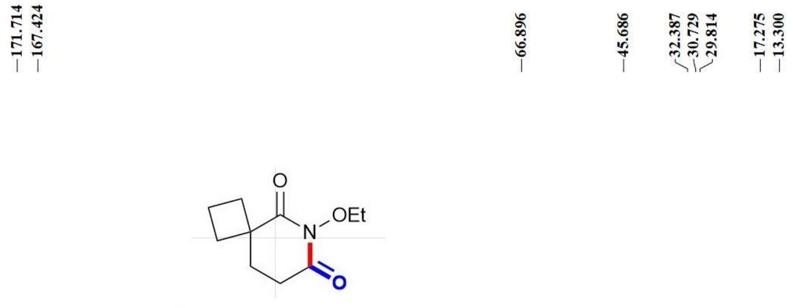

^{13}C NMR (100 M, $CDCl_3$)

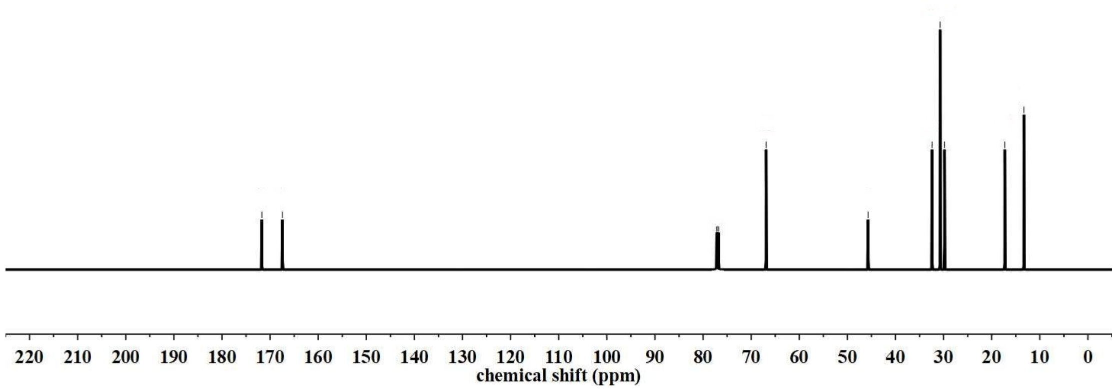

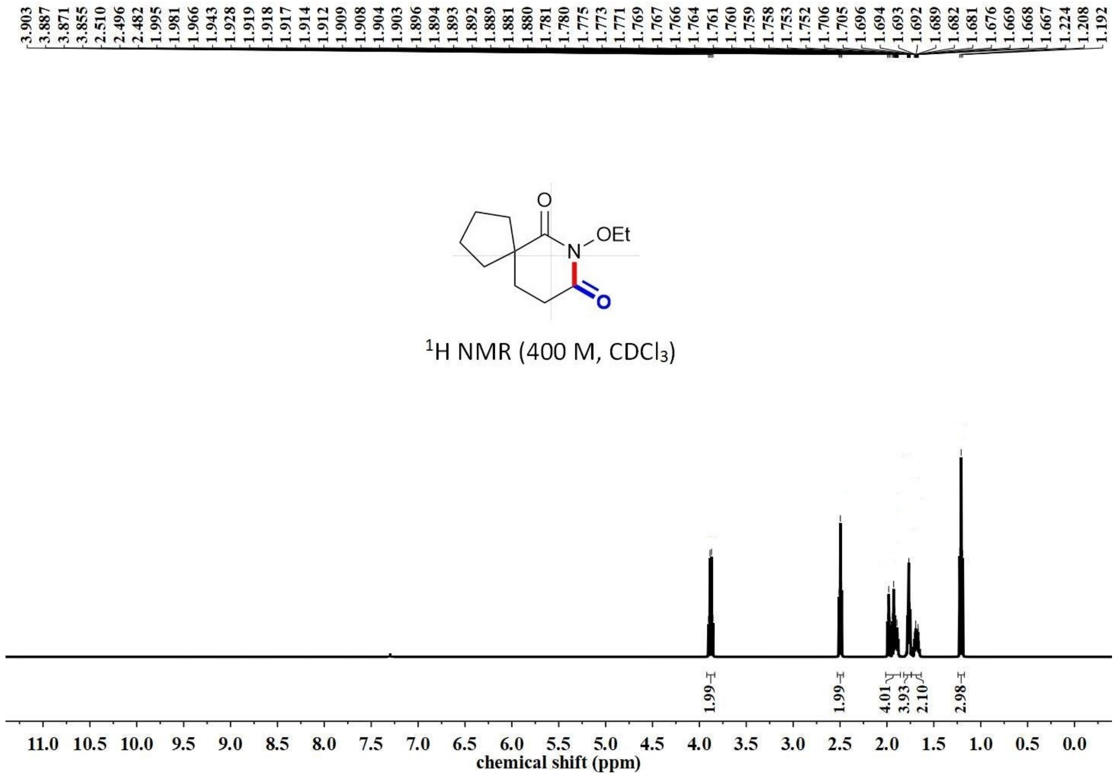

^1H NMR (400 M, $CDCl_3$)

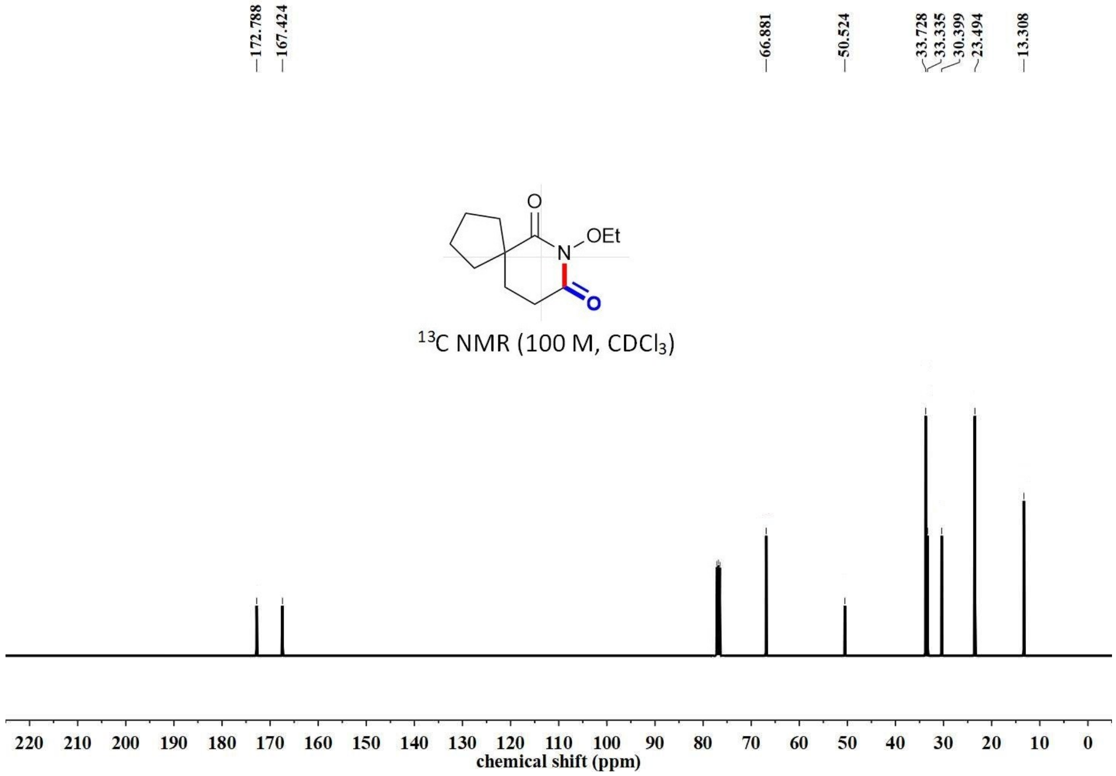

^{13}C NMR (100 M, $CDCl_3$)

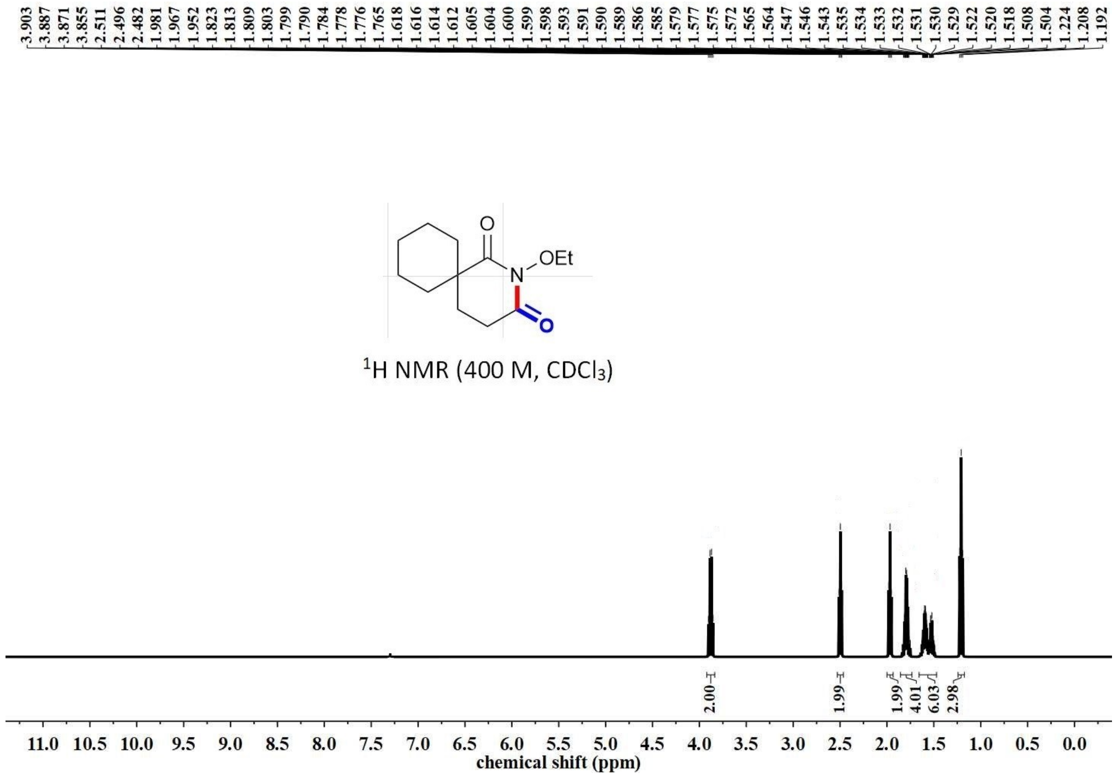

^{1}H NMR (400 M, $CDCl_3$)

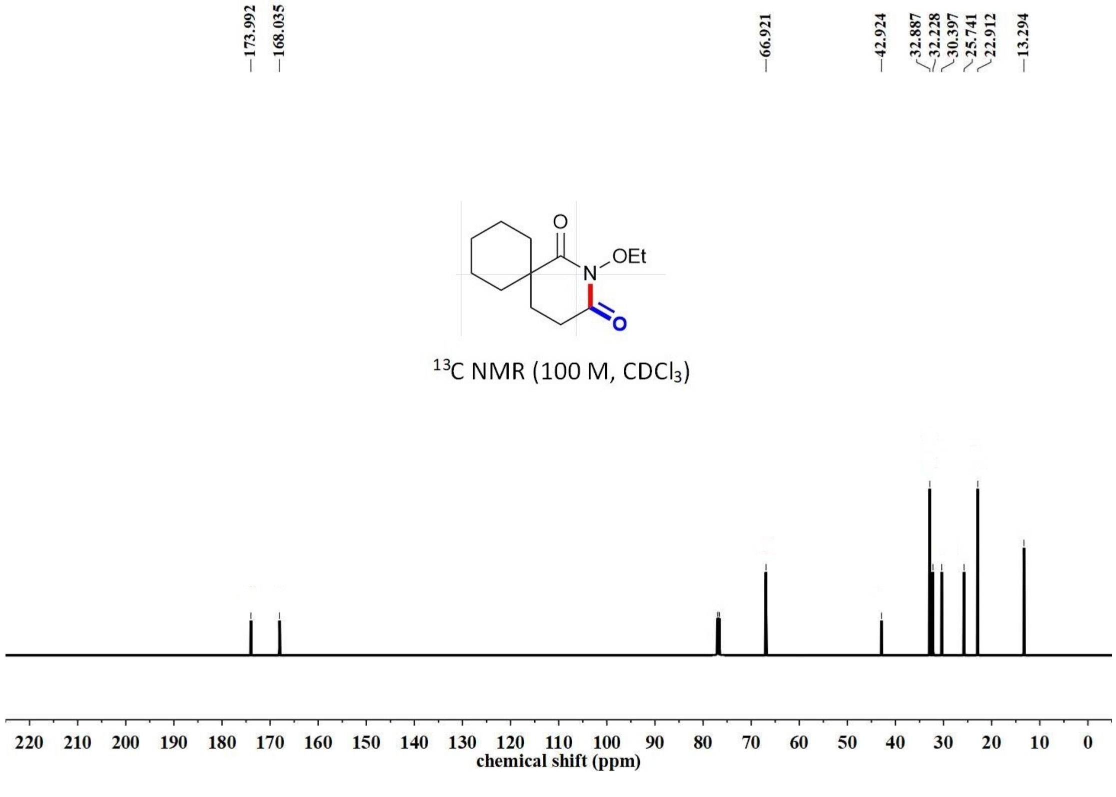

^{13}C NMR (100 M, $CDCl_3$)

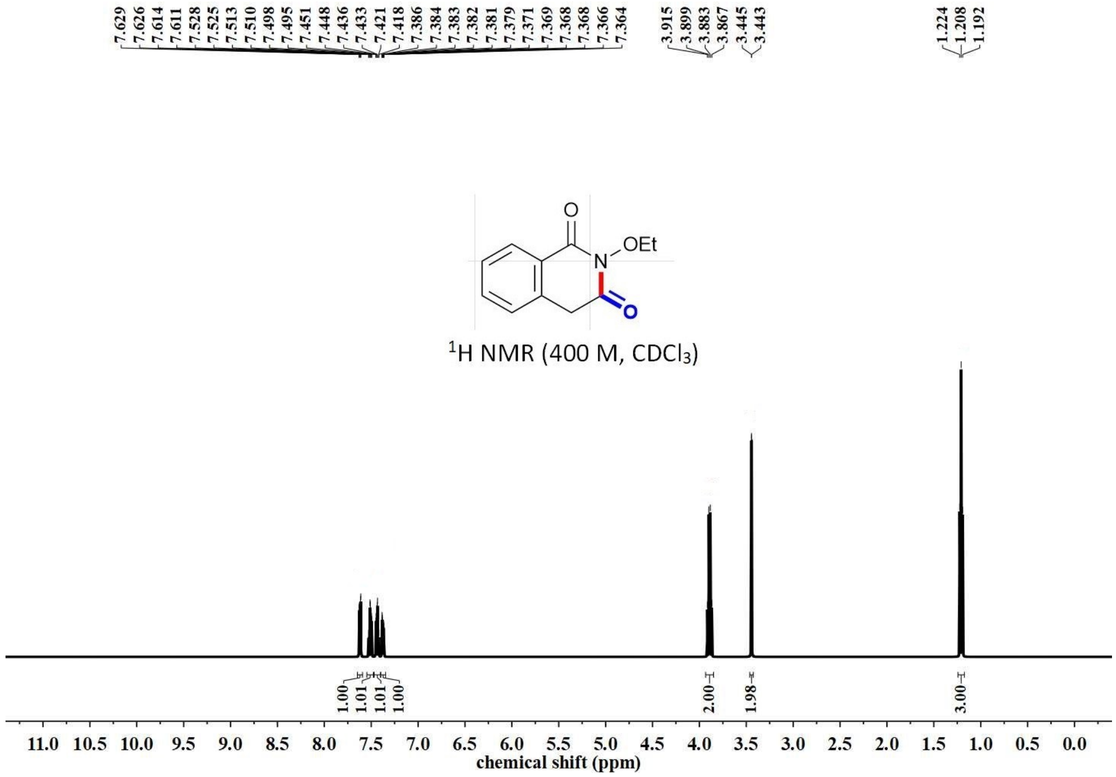
7.629 7.626 7.614 7.611 7.528 7.525 7.513 7.510 7.498 7.495 7.451 7.448 7.436 7.433 7.421 7.418 7.386 7.384 7.383 7.382 7.381 7.379 7.371 7.369 7.368 7.368 7.366 7.364
3.915 3.899 3.883 3.867 3.445 3.443
1.224 1.208 1.192
OEt
^{1}H NMR (400 M, $CDCl_3$)
1.00 1.01 1.01 1.00
2.00
1.98
3.00
11.0 10.5 10.0 9.5 9.0 8.5 8.0 7.5 7.0 6.5 6.0 5.5 5.0 4.5 4.0 3.5 3.0 2.5 2.0 1.5 1.0 0.5 0.0
chemical shift (ppm)

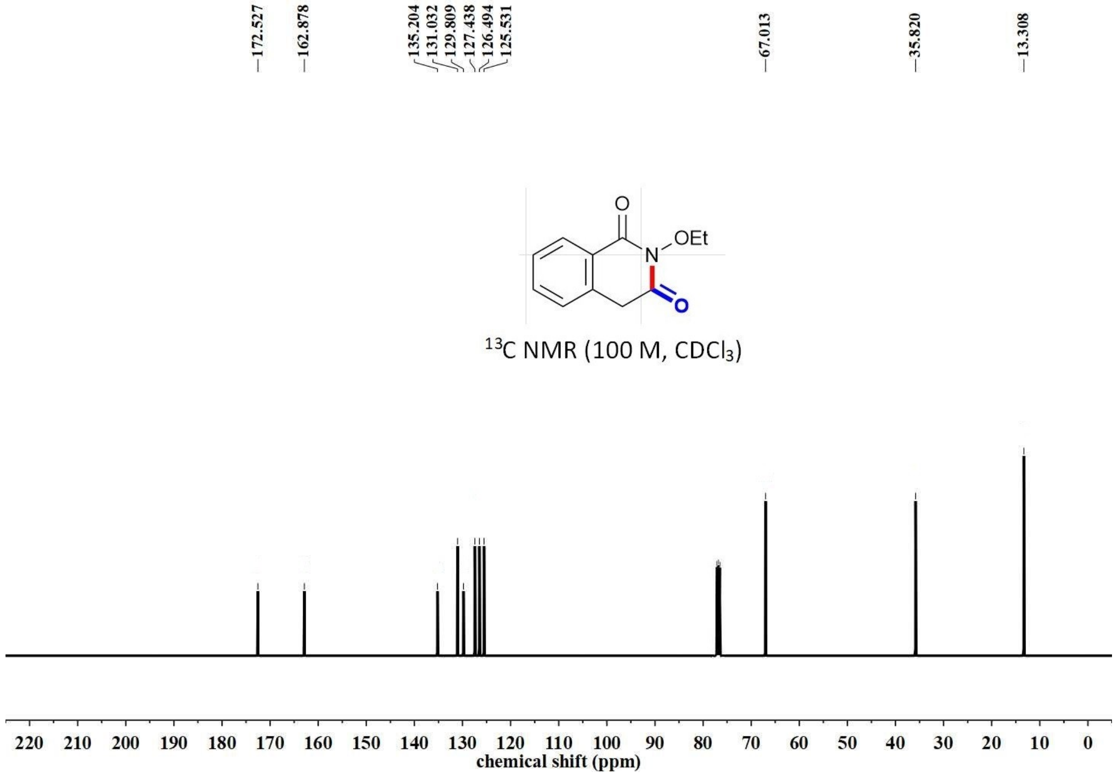
172.527
162.878
135.204 131.032 129.809 127.438 126.494 125.531
67.013
35.820
13.308
OEt
^{13}C NMR (100 M, $CDCl_3$)
220 210 200 190 180 170 160 150 140 130 120 110 100 90 80 70 60 50 40 30 20 10 0
chemical shift (ppm)

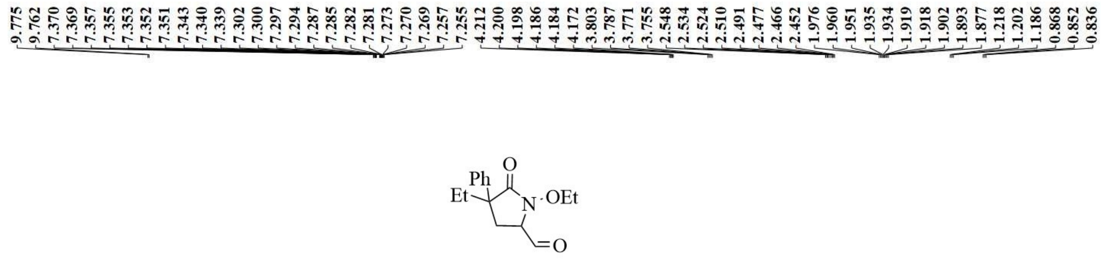

^{1}H NMR ($CDCl_3$, 400 MHz)

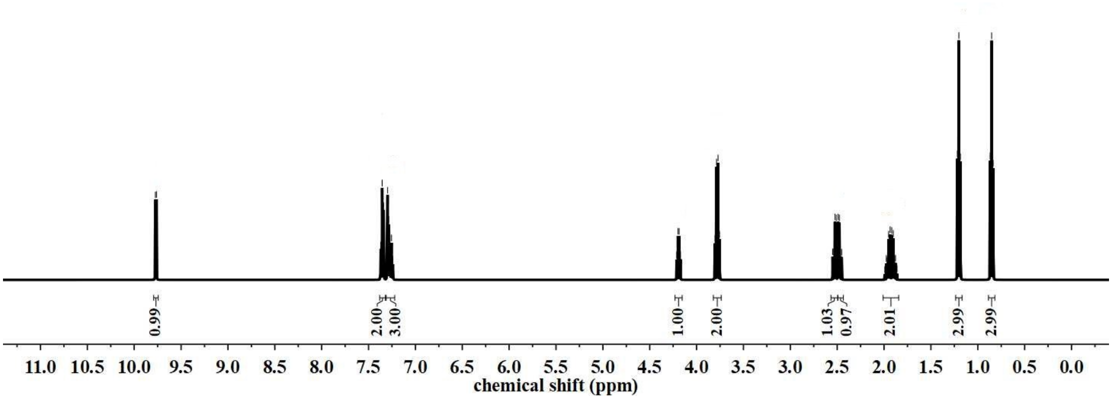

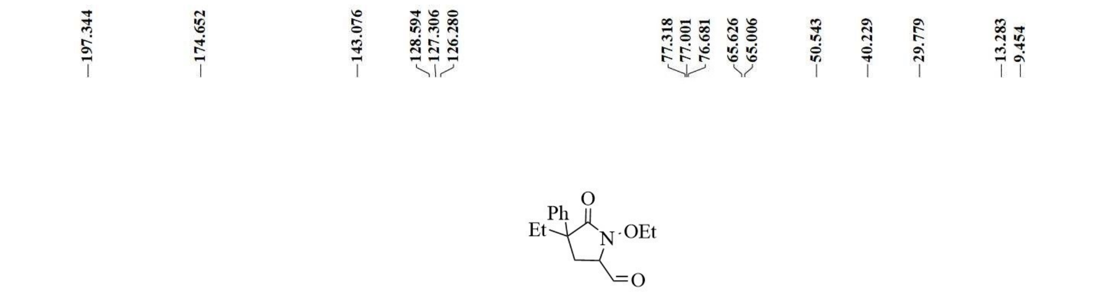

^{13}C NMR ($CDCl_3$, 101 MHz)

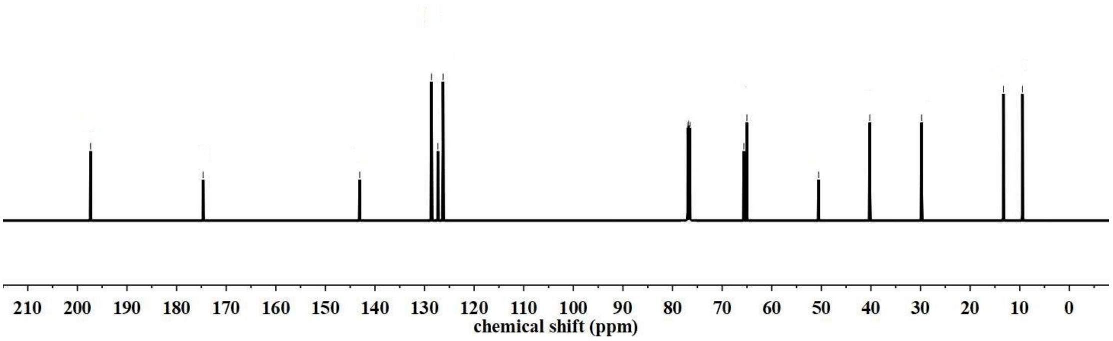

4. ESI-MS measurement: instrument parameters and results

Acquisition Mass Control	
Detection Mode	Broad band
TD (Acquisition)	1 M
Low Mass	57.75 m/z
High Mass	2000.00 m/z
Estimated R.P.	38000
Transient Length	0.2097 sec
Data transfer mode	standard

API Source		Source Gas Tune		Source Gas Control	
Source Type	ESI	Dry Gas	4.0 L/min	Drying Gas Flow	Yes
Capillary -	2600 V	Dry Temp	100 ^{o}C	Drying Gas Heater	Yes
End Plate Offset	-500 V	Nebulizer	1.0 bar	Nebulizer Gas Flow	Yes
Corona Needle	0.0 nA	Vaporizar Temp	10.0 ^{o}C	Nebulizer Gas Heater	No

Figure S1 ESI-MS spectra of mechanism research

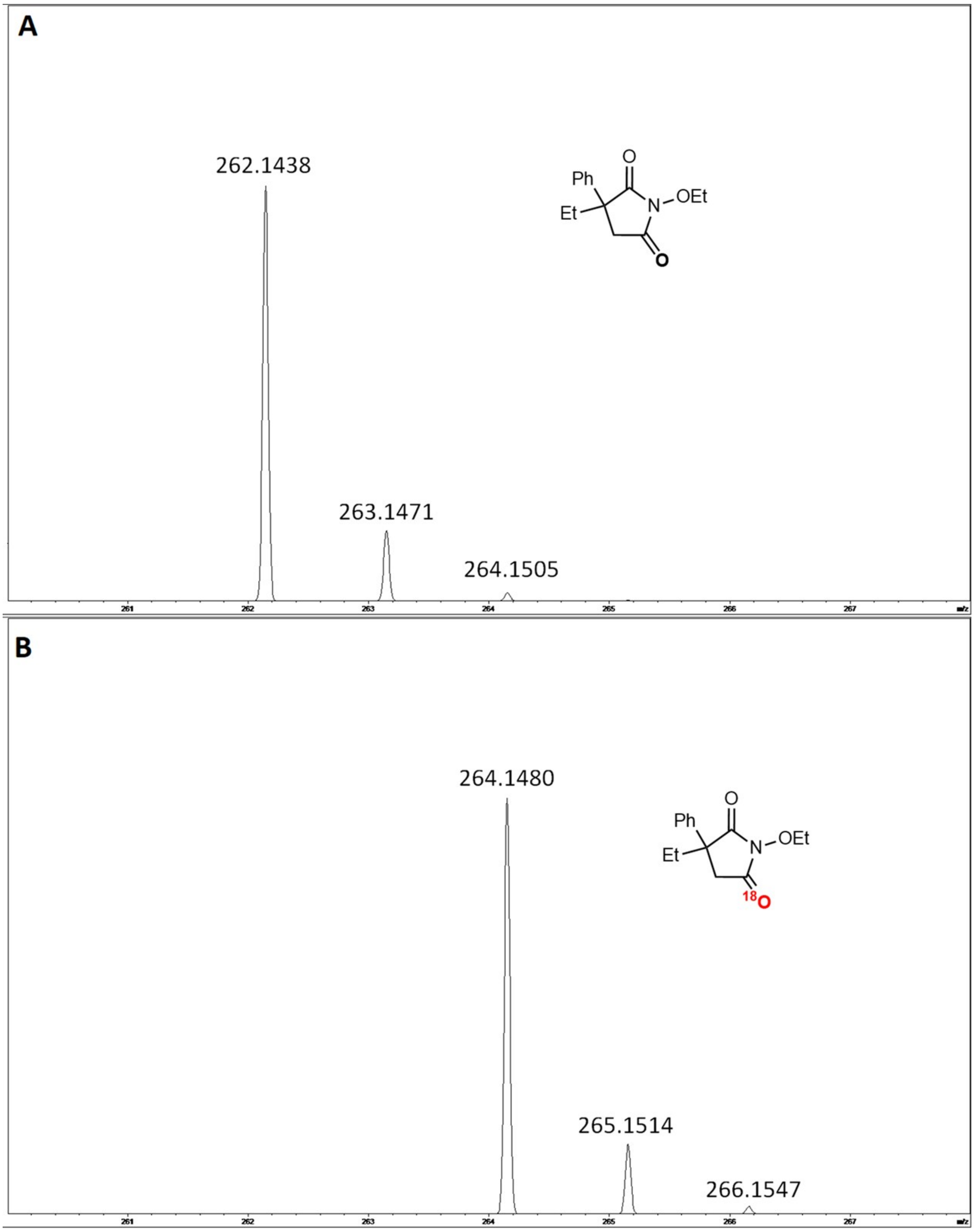

Supplement: RA-010-C9RA10422D-s001 [file RA-010-C9RA10422D-s001.pdf]
